# Successful Human Infection with *P. falciparum* Using Three Aseptic *Anopheles stephensi* Mosquitoes: A New Model for Controlled Human Malaria Infection

**DOI:** 10.1371/journal.pone.0068969

**Published:** 2013-07-16

**Authors:** Matthew B. Laurens, Peter Billingsley, Adam Richman, Abraham G. Eappen, Matthew Adams, Tao Li, Sumana Chakravarty, Anusha Gunasekera, Christopher G. Jacob, B. Kim Lee Sim, Robert Edelman, Christopher V. Plowe, Stephen L. Hoffman, Kirsten E. Lyke

**Affiliations:** 1 Howard Hughes Medical Institute/Center for Vaccine Development, University of Maryland School of Medicine, Baltimore, Maryland, United States of America; 2 Sanaria, Inc., Rockville, Maryland, United States of America; 3 Protein Potential LLC, Rockville, Maryland, United States of America; University of California Los Angeles, United States of America

## Abstract

Controlled human malaria infection (CHMI) is a powerful method for assessing the efficacy of anti-malaria vaccines and drugs targeting pre-erythrocytic and erythrocytic stages of the parasite. CHMI has heretofore required the bites of 5 *Plasmodium falciparum* (Pf) sporozoite (SPZ)-infected mosquitoes to reliably induce Pf malaria. We reported that CHMI using the bites of 3 PfSPZ-infected mosquitoes reared aseptically in compliance with current good manufacturing practices (cGMP) was successful in 6 participants. Here, we report results from a subsequent CHMI study using 3 PfSPZ-infected mosquitoes reared aseptically to validate the initial clinical trial. We also compare results of safety, tolerability, and transmission dynamics in participants undergoing CHMI using 3 PfSPZ-infected mosquitoes reared aseptically to published studies of CHMI using 5 mosquitoes. Nineteen adults aged 18–40 years were bitten by 3 *Anopheles stephensi* mosquitoes infected with the chloroquine-sensitive NF54 strain of Pf. All 19 participants developed malaria (100%); 12 of 19 (63%) on Day 11. The mean pre-patent period was 258.3 hours (range 210.5–333.8). The geometric mean parasitemia at first diagnosis by microscopy was 9.5 parasites/µL (range 2–44). Quantitative polymerase chain reaction (qPCR) detected parasites an average of 79.8 hours (range 43.8–116.7) before microscopy. The mosquitoes had a geometric mean of 37,894 PfSPZ/mosquito (range 3,500–152,200). Exposure to the bites of 3 aseptically-raised, PfSPZ-infected mosquitoes is a safe, effective procedure for CHMI in malaria-naïve adults. The aseptic model should be considered as a new standard for CHMI trials in non-endemic areas. Microscopy is the gold standard used for the diagnosis of Pf malaria after CHMI, but qPCR identifies parasites earlier. If qPCR continues to be shown to be highly specific, and can be made to be practical, rapid, and standardized, it should be considered as an alternative for diagnosis.

**Trial Registration:**

ClinicalTrials.gov NCT00744133 NCT00744133

## Introduction

Controlled human malaria infection (CHMI) using mosquitoes infected on cultures containing Pf gametocyte-infected erythrocytes has been shown to be safe and effective for testing the efficacy of anti-malarial drugs and malaria vaccine candidates for more than 25 years, including early studies of subunit malaria vaccines and atovaquone efficacy [1]–[7]. CHMI is increasingly recognized as essential for testing the efficacy of malaria vaccine candidates targeting pre-erythrocytic and erythrocytic stages of the parasite. The most advanced malaria vaccine candidate to date, RTS,S/AS01, relied on CHMI to optimize dosing, formulation, and the immunization regimen [8]–[11]. Recognizing the important role that CHMI plays in malaria vaccine development and the increased need for centers that conduct this type of specialized clinical trial [12], the World Health Organization (WHO) recently prioritized optimization of the current CHMI model [13], [14].

The exposure of malaria-naïve persons to the bites of infected mosquitoes has been performed for almost 100 years [15], [16]. The earliest recorded use of purposeful exposure to malaria, termed malariatherapy, resulted in a Nobel Prize to Julius Wagner-Juaregg for the treatment of general paresis of the insane, or neurosyphilis [16]. CHMI was pioneered for vaccine development in the early 1970s before Pf could be cultured *in vitro* [17], [18], and became an established method to test candidate malaria vaccine and drug efficacy during the 1980s [3], [4], [6], [7], [19]. Since that time, numerous malaria vaccine candidates have been tested using CHMI [6], [7].

The traditional CHMI model requires the bites of 5 infected, insectary-raised mosquitoes to reliably induce malaria. Disadvantages of this model include the requirement for an insectary and entomology expertise, secure transportation from the insectary to the clinical trial site, precise timing of mosquito rearing to coordinate with malaria candidate vaccine dosing, and the theoretical risk of participant exposure to microorganisms other than malaria that may be carried by standard laboratory-raised mosquitoes [20], [21]. A cGMP-produced, aseptic mosquito reduces this latter theoretical risk, and was conceived as a preliminary step towards the administration of malaria sporozoites parenterally by needle and syringe, which would overcome the practical disadvantages of CHMI using mosquitoes.

We previously reported on an improved CHMI system using the bites of 1, 3 or 5 aseptically-raised mosquitoes. This study demonstrated that the aseptic model is safe, associated with a precise pre-patent period, and transmitted malaria to all 6 participants (100%) bitten by 3 *Anopheles stephensi* mosquitoes [22]. In the current study, we evaluated the aseptic model using the bites of 3 mosquitoes in an additional 19 malaria-naïve adults, for a combined total of 25 adults, to confirm the efficiency of the 3 mosquito model and assessed the safety, tolerability, and transmission dynamics as compared to those reported in published CHMI studies.

## Methods

### Ethics Statement

The study was approved by the University of Maryland School of Medicine Institutional Review Board and was conducted in compliance with the Declaration of Helsinki. Independent site monitoring was conducted to ensure that human subject protection, study procedures, laboratory procedures, study intervention administration, and data collection processes met GCP/ICH and regulatory guidelines. An independent safety monitoring committee was convened to review study participant safety data and clinical results. The clinical trial was registered on clinicaltrials.gov, registry number NCT00744133.

### Objectives

The main objective of this study was to validate in a larger study our preliminary results on the minimum number of aseptic, PfSPZ-infected *A. stephensi* required to achieve 100% Pf parasitemia in malaria-naïve adults [22]. The study outcomes assessed included occurrence of a positive thick blood smear in the 56-day follow-up period following CHMI, occurrence and severity of solicited and unsolicited adverse events (AEs) after CHMI, and occurrence of serious AEs throughout the study. A secondary outcome was the occurrence of real time quantitative polymerase chain reaction (qPCR) positivity for Pf DNA conducted on samples retrospectively during the 56-day surveillance period. We compared results from the previous cohort of 6 participants to the current cohort of 19 participants bitten by 3 aseptic mosquitoes. Combined results from all 25 participants were then compared to results from 18 infectivity control participants [23] who underwent traditional CHMI using 5 mosquitoes and whose samples were evaluated for qPCR using identical methods in our laboratory. The protocol for this trial and supporting CONSORT checklist are available as supporting information (See Checklist S1 and Protocol S1).

### Study Population and Design

Healthy, malaria-naïve U.S. volunteers aged 18–40 years were recruited to be infected by the bites of 3 aseptically raised *A. stephensi* mosquitoes infected with the chloroquine-sensitive NF54 strain of Pf at the Center for Vaccine Development at the University of Maryland School of Medicine in Baltimore, Maryland. All participants provided written informed consent prior to study screening procedures, including documentation of medical history, assessment of cardiovascular risk [24], a physical examination, and laboratory analyses (urinalysis, hemoglobin, hemoglobin electrophoresis, white blood cell count, platelet count, serum aspartate aminotransferase (AST), alanine aminotransferase (ALT), glucose, and creatinine). Results were combined with previously collected data in 6 individuals who underwent CHMI by exposure to the bites of 3 Pf-infected mosquitoes [23].

### Dosage, Preparation and Administration of Study Product

Details of the administration of the study product including dosage, preparation, and quantification of salivary gland assessment; post-exposure assessment; evaluation of safety and tolerability; and malaria diagnostics were identical to those reported previously [22]. Briefly, participants who met eligibility criteria were exposed to 3 aseptically-reared, Pf*-*infected *A. stephensi* mosquitoes [23], [25] for 5 minutes. Mosquitoes were then euthanized and the midgut was examined to confirm a blood meal was taken. The paired salivary glands from each mosquito were removed by dissection to verify that at least 1000 PfSPZ per mosquito were present and to quantify PfSPZ load [22]. If necessary, additional mosquitoes were used until exactly 3 PfSPZ-infected mosquitoes had fed on each study participant.

### Assessment of Safety and Tolerability

All adverse events were graded for severity and relatedness and solicited symptoms and signs were assessed as previously described [22]. Due to an adverse cardiac event that occurred in the setting of malaria challenge at another challenge center, electrocardiograms (ECG) and troponin levels were done on day 3 and 10 after the malaria diagnosis for exploratory purposes [26].Safety labs including a complete blood count and serum creatinine, glucose, AST, and ALT were drawn on all days of positive blood smear.

### Malaria Diagnostics

Daily malaria diagnostic screening began 5 days after CHMI by direct examination of thick blood smears for presence of Pf parasites. All therapeutic decisions were based on thick blood smear results. If a participant developed symptoms or signs consistent with malaria, additional thick blood smears were evaluated at 8–12 hour intervals.

Blood smears were prepared as previously described [22] with 10 µL of blood and Giemsa-staining for Pf parasites. Two trained investigators each examined approximately 0.5 µL of blood using the 100x oil immersion lens of calibrated microscopes (1000x magnification). This was doubled to approximately 1 µL of blood for symptomatic individuals. Parasite density was quantified as number of asexual parasite forms/µL. A senior malaria microscopist (MBL) confirmed smear positivity, quantified parasite burden for all positive smears, and resolved discrepancies. The minimum criterion for a positive smear was identification of 2 unquestionable Pf parasites confirmed by at least 1 investigator and MBL. Participants with positive microscopy results were treated with 1500 mg of chloroquine base over 48 hours. Two participants missed follow-up visits on the day before patency by microscopy and were excluded from analyses of microscopy patency.

qPCR was performed on DNA extracted from 0.5 mL of venous blood collected contemporaneously with blood smears, using published methods with minor adaptation [27]. PCR primers were based on the published sequence of the highly conserved [28], stage-specific [6] Pf 18S ribosomal RNA gene. Primer sequences were identical to the corresponding sequence of the NF54 strain. Samples were blinded and assays were run daily. Each sample was run in duplicate along with a negative control. A standard curve consisting of 200 k, 20 k, 2 k, 200, and 20 parasites per mL diluted in whole blood, then leukocyte-depleted, were run in each plate. The data were analyzed using the Applied Biosystem 7300 Absolute Quantification Software. The assay limit of sensitivity was determined to be 40 parasites/mL. Study investigators and blood smear readers were blinded to qPCR results.

### Statistical Analysis

Sample size calculations factored in the 6 individuals from the previous study [22] added to an additional 20 volunteers (n = 26) anticipated in the current study. Assuming a 100% infectivity rate for 26 volunteers, the 95% confidence interval (CI) for infectivity would be 83–100%. The differences in total sporozoites present in dissected mosquitoes, parasite quantification by group, and days to positive patency were calculated using a two-tailed Mann Whitney test. Statistical calculations were performed using GraphPad Prism® version 5.01, GraphPad Software, San Diego, California, USA. The blood-stage parasite multiplication rate was calculated as described in the literature by Bejon et al. [29] and Roestenberg et al. [30] and represents the fold-replication of Pf parasites in 48 hours. A representation of the multiplication rate was evaluated graphically for each participant and those with a negative slope as well as those with fewer than 4 qPCR measurements were not included in the analysis.

## Results

### Study Population and Malaria Challenge Event

Thirty-six potential participants underwent screening, and 20 who met study inclusion criteria were scheduled for CHMI (Figure S1). One individual failed to return on the day of CHMI, and the remaining 19 participants (8 females) aged 20–39 (mean 29.8 years) were enrolled and underwent CHMI in October 2010. Results from all 19 participants were added to 6 previously challenged individuals for a combined total of 25.

A total of 142 mosquitoes were required on the day of CHMI (mean 7.5 per participant). Of these, 88 mosquitoes (62%) took a blood meal and were dissected to determine the presence and quantity of PfSPZ. Fifty-seven mosquitoes (65%) were found to have detectable PfSPZs with a geometric mean PfSPZ load of 37,894 per mosquito (range 3500–152,000). No mosquito had fewer than 1,000 PfSPZs per paired salivary glands. Participants were exposed to 3 mosquitoes with a combined total geometric mean PfSPZ load of 132,269 (range 48,000–240,000). This was a greater number of PfSPZ per 3 mosquitoes than the 6 participants exposed to 3 mosquitoes in the previous study [22], who were exposed a total geometric mean PfSPZ load of 57,187 during CHMI (range 18,000–112,000 (p = 0.003 by Mann Whitney test). Table 1 summarizes demographic information and study results for all participants infected with 3 mosquitoes, including the 6 subjects from the previous trial. Table S1 lists qPCR results for all 25 participants infected using 3 mosquitoes reared aseptically.

**Table 1:** Demographic information and study results for participants receiving *Plasmodium falciparum* sporozoites by the bites of 3 aseptic mosquitoes.

Number of subjects	Mean age in years (range)	Male n (%)	Total mosquitoes presented (mean per person)	Total mosquitoes taking blood meal (% of total presented)	Total infected mosquitoes (% of those taking a blood meal)	Geometric mean PfSPZ per mosquito (range)	Geometric mean total PfSPZ per participant (range)	Mean prepatent period by thick smear in days (range)	Mean day of initial qPCR detection (range)	Geometric mean parasite density/µL at first positive thick blood smear (range)	Geometric mean parasite density/µL at first positive qPCR (range)
6^a^	28.7 (20–34)	5 (83)	40 (6.7)	26 (65)	18 (69)	16,646 (2500–57,500)	57,187 (18,000–112,000)	10.8 (10–11)	7.5 (7–8)	15.5 (8–32)	0.2 (0.04–0.5)
19^b^	29.8 (20–39)	11 (58)	142 (7.5)	98 (69)	57 (58)	37,894 (3500–152,000)	132,269 (48,000–240,000)	10.9 (9–14)	7.5 (7–12)	9.5 (2–44)	1.7 (0.1–18.2)
**TOTAL** N = 25	29.5 (20–39)	16 (64)	182 (7.3)	124 (68)	75 (60)	31,105 (2500–152,000)	108,158 (18,000–240,000)	10.9 (9–14)	7.5 (7–12)	10.8 (2–44)	0.9 (0.04–18.2)

a Previous Study [22].

b Current Study.

### Safety Assessment

Local symptoms and signs were solicited from days 2–7 after CHMI and are presented in Table 2 for participants exposed to 3 mosquitoes from the combined studies. Reactogenicity data from days 0–1 after CHMI were not collected because mild reactogenicity to the mosquito exposure was expected in all volunteers. Severe solicited symptoms and signs were detected in only 1 individual and included local erythema and induration of 5.1 cm that resolved by day 3 after CHMI. Moderate solicited reactogenicity events included 2 instances of erythema and malaise and 1 headache, all reported in different participants. Mild reactogenicity was reported most commonly as erythema, induration, and malaise, occurring in a minority of participants. Two participants reported mild symptoms that continued until malaria patency: 1 experienced mild arthralgia starting on day 7 and another described malaise starting on days 6 and 7. These symptoms were attributed to malaria illness. The only unsolicited event reported during the 7 days after mosquito exposure and deemed associated with mosquito biting was pruritus at the bite site.

**Table 2:** Maximum intensity of solicited symptoms and signs during days 2–7 after receiving Plasmodium falciparum sporozoites by the bites of 3 aseptic mosquitoes.

	Study Group											
	Previous Study [22] (n = 6)				Current Study (n = 19)				Total (n = 25)			
Symptom	None (%)	Mild (%)	Moderate (%)	Severe (%)	None (%)	Mild (%)	Moderate (%)	Severe (%)	None (%)	Mild (%)	Moderate (%)	Severe (%)
**Local**												
Erythema	2 (33.3)	4 (66.7)	0 (0)	0 (0)	11 (57.9)	6 (31.6)	2 (10.5)	0 (0)	13 (52.0)	10 (40.0)	2 (8.0)	1 (4.0)
Induration	4 (66.7)	2 (33.3)	0 (0)	0 (0)	13 (68.4)	5 (26.3)	0(0)	1 (5.3)	17 (68.0)	7 (28.0)	0 (0)	1 (4.0)
Site Pain	6 (100)	0 (0)	0 (0)	0 (0)	17 (89.5)	1 (5.3)	0(0)	1 (5.3)	23 (92.0)	1 (4.0)	0 (0)	0 (0)
**Systemic**												
Malaise	4(66.7)	1 (16.7)	1 (16.7)	0 (0)	14 (73.7)	4 (21.1)	1 (5.3)	0 (0)	18 (72.0)	5 (20.0)	2 (8.0)	0 (0)
Myalgia	6 (100)	0 (0)	0 (0)	0 (0)	19 (100)	0 (0)	0 (0)	0 (0)	25 (100.0)	0 (0)	0 (0)	0 (0)
Arthralgia	6 (100)	0 (0)	0 (0)	0 (0)	18 (94.7)	1 (5.3)	0 (0)	0 (0)	24 (96.0)	1 (4.0)	0 (0)	0 (0)
Nausea	5(83.3)	1 (16.7)	0 (0)	0 (0)	18 (94.7)	1 (5.3)	0 (0)	0 (0)	23 (92.0)	2 (8.0)	0 (0)	0 (0)
Abdominal Pain	6 (100)	0 (0)	0 (0)	0 (0)	19 (100)	0 (0)	0 (0)	0 (0)	25 (100.0)	0 (0)	0 (0)	0 (0)
Diarrhea	4 (66.7)	2 (33.3)	0 (0)	0 (0)	19 (100)	0 (0)	0 (0)	0 (0)	23 (92.0)	2 (8.0)	0 (0)	0 (0)
Fever	6 (100)	0 (0)	0 (0)	0 (0)	16 (84.2)	3 (15.8)	0 (0)	0 (0)	22 (88.0)	3 (12.0)	0 (0)	0 (0)
Urticaria	6 (100)	0 (0)	0 (0)	0 (0)	18 (94.7)	1 (5.3)	0 (0)	0 (0)	24 (96.0)	1 (4.0)	0 (0)	0 (0)
Headache	4 (66.7)	2 (33.3)	0 (0)	0 (0)	18 (94.7)	0 (0)	1 (5.3)	0 (0)	22 (88.0)	2 (8.0)	1 (4.0)	0 (0)

### Malaria Diagnostics

All 19 participants (100%) developed patent parasitemia by microscopy, with 12/17 (71%) detected on Day 11 after mosquito exposure. Two participants missed study visits including Day 11 just before patency detection by microscopy and were not included in analyses of patency by microscopy. The geometric mean time to patency by microscopy was 258 hours (range 211–334) or 10.8 days (range 8.8–13.9). Geometric mean parasite density by microscopy at patency was 9.5 parasites/µL (range 2–44). Compiled microscopy data from 23 participants bitten by 3 mosquitoes, incorporating results from the previous study, shows similar results, with 17 (74%) developing patent parasitemia 11 days after exposure, a geometric mean time to patency of 257 hours (range 210.5–333.8) or 10.7 days (range 8.8–13.9), and geometric mean parasite density of 10.8 parasites/µL at diagnosis (range 2–44).

Parasitemia by qPCR was detected in all 19 participants (100%); 14 (74%) became positive on day 7 after exposure. The geometric mean time from exposure to detection of parasitemia by qPCR was 177 hours (range 162–286) or 7.4 days (range 6.7–11.9). Geometric mean parasite density by qPCR analysis on the first day of qPCR positivity was 1.66 parasites/µL (range 0.11–18.21). Combined qPCR data from all 25 participants bitten by 3 mosquitoes shows similar results with 17/25 (68%) turning positive on Day 7 post-exposure. For these same 25 participants, the geometric mean parasite density on the first day of qPCR positivity was 0.88 parasites/µL (range 0.42–18.21). Combined data from 23 participants of both studies exposed to 3 aseptic mosquitoes demonstrated that qPCR detected patency an average of 79.0 hours before microscopy (95% CI 70.0–88.0). Before smear detection, no false positive or false negative qPCR assays were detected. Geometric mean parasite density on the first day of qPCR positivity in the current study was greater than the 6 participants exposed to 3 mosquitoes in the initial study [22] (3.63 versus 0.16 parasites/µL, p = 0.002 by Mann Whitney test). Evaluation of the relationship of total mosquito PfSPZ load to parasite density by qPCR analysis on the first day of qPCR positivity for all 25 participants exposed to 3 mosquitoes showed no correlation (R^2^ = 0.05).

**Figure 1:**
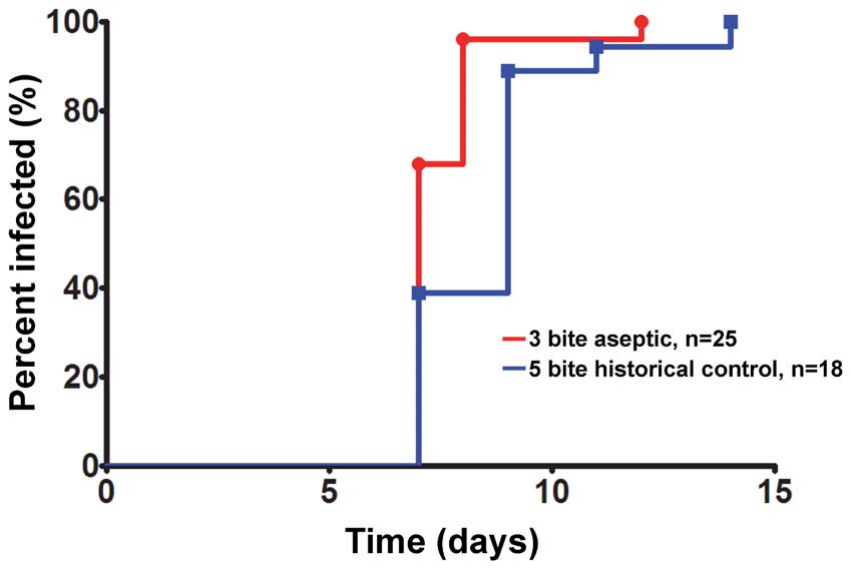
Percent of participants infected over time by quantitative PCR, 3 aseptic mosquito participants compared to 5 mosquito historical controls [23].

**Figure 2:**
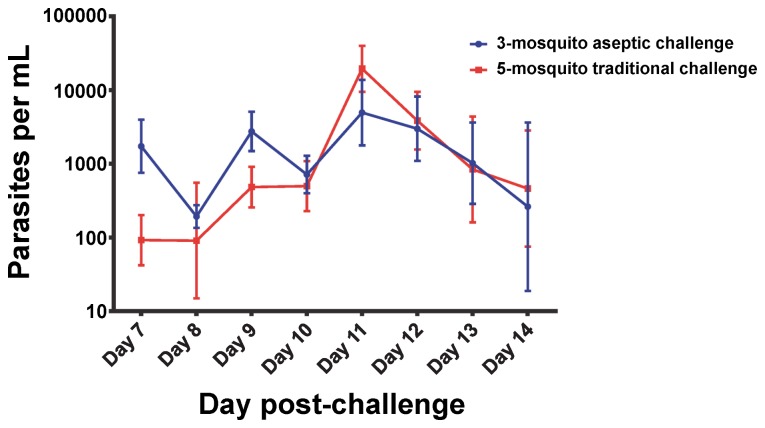
Comparison of geomean quantitative PCR diagnostics following challenge with 3 aseptic mosquitoes versus the traditional 5 mosquito challenge [23]. Error bars represent 95% confidence intervals.

We compared our malaria diagnostic results in the 25 study participants exposed to 3 mosquitoes in the current study and our previous study [22] to those of 18 unvaccinated infectivity controls from an unrelated study with NF54 parasites using the traditional 5 mosquito methodology and identical microscopy and qPCR diagnostic techniques [23]. The traditional 5 mosquito CHMI resulted in a longer time (in study days) to patent parasitemia by microscopy (10.9 vs. 11.5 days, p = 0.04 by Mann Whitney test). Geometric mean parasitemia levels at malaria diagnosis were higher in the traditional CHMI compared to the 3 mosquito aseptic challenge (49.3 vs. 15.2 parasites/µL; p = 0.03 by Mann Whitney test). Detection by qPCR measured by study day occurred an average of 1.1 days earlier (7.5 vs. 8.6 days, p = 0.006 by Mann Whitney test) in the 3 mosquito aseptic challenge compared to traditional CHMI (Figure 1). Moreover, the geometric mean parasite density at time of first qPCR positivity was higher in the 3 mosquito aseptic challenge compared to the traditional CHMI (2.80 vs. 0.38/µL, p = 0.003 by Mann Whitney test). The results of qPCR for both studies are shown in Figure 2.

While we performed the qPCR analysis primarily for diagnostic purposes, we calculated the parasite multiplication rate for all 25 participants exposed to 3 mosquitoes reared aseptically. Using the sine wave method described by Bejon et al. [29] and accounting for time in hours, the geometric mean parasite multiplication rate was 4.4. Using the model proposed by Roestenberg et al. [30], the geometric mean parasite multiplication rate was 5.2. Both results fall within the range of parasite multiplication rates reported at other centers conducting CHMI [30]. By convention, all participants with fewer than 4 qPCR positive measurements (n = 6), or demonstrating a negative slope (n = 3), were excluded from analysis for a total of 15 evaluable samples.

### Clinical Malaria

A total of 25 participants combined from the previous and current study were evaluated. We removed two individuals from diagnostic analysis due to a missed study visit, which would alter conclusions. Eight of the remaining 23 participants (35%) bitten by 3 mosquitoes developed clinical symptoms consistent with malaria before diagnosis by thick blood smear, while 11/23 (48%) developed symptoms on the day of diagnosis and 6/23 (17%) of the participants were symptom-free until the days after blood smear diagnosis. Among the 8 volunteers who were symptomatic before diagnosis, symptoms developed a mean of 2.8 days (range 1–5) before diagnosis by microscopy. All symptoms were reported as mild and did not interfere with daily activities. No individuals experienced temperature above 37.5°C (lower threshold for Grade 1 fever) on the days before malaria diagnosis. All of the remaining 6 volunteers developed symptoms or signs consistent with malaria within 24 hours after diagnosis and initiation of treatment. Fever ≥37.5°C was recorded in 19 participants (76%) on the day of diagnosis. The mean duration of fever was 2.3 days (range 1–4 days). Severe fever (>39.0°C) was the only solicited severe symptom recorded during the time of malaria illness and was present in 11/25 participants (44%). The most common solicited symptoms and signs during the malaria illness period were headache (88%), measured fever (76%), malaise (76%), chills (76%), and myalgia (72%) (Table 3). Solicited symptoms peaked in number and intensity during the 24-hour period after malaria diagnosis and treatment initiation.

**Table 3:** Solicited symptoms and signs during malaria illness days 8–18 after receiving Plasmodium falciparum sporozoites by the bites of 3 aseptic mosquitoes.

	Study Group												
	Initial Study [22] (n = 6)				Current Study (n = 19)				Total (n = 25)				Past Results [7] (N = 47)
Symptom	Mild (%)	Moderate (%)	Severe (%)	Any (%)	Mild (%)	Moderate (%)	Severe (%)	Any (%)	Mild (%)	Moderate (%)	Severe (%)	Any (%)	Any (%)
Fever	1 (17)	2 (33)	2 (33)	5 (83)	2 (11)	3 (16)	9 (47)	14 (74)	3 (12)	5 (15)	11 (44)	19 (76)	47 (100)
Headache	3 (50)	2 (33)	0 (0)	5 (83)	12 (63)	5 (26)	0 (0)	17 (89)	15 (60)	7 (28)	0 (0)	22 (88)	47 (100)
Malaise	2 (33)	3 (50)	0 (0)	5 (83)	8 (42)	6 (32)	0 (0)	14 (74)	10 (40)	9 (36)	0 (0)	19 (76)	44 (94)
Chills	2 (33)	2 (33)	0 (0)	3 (50)	9 (47)	7 (37)	0 (0)	16 (79)	11 (44)	9 (36)	0 (0)	19 (76)	40 (85)
Myalgia	2 (33)	2 (33)	0 (0)	4 (67)	9 (47)	5 (26)	0 (0)	14 (74)	11 (44)	7 (28)	0 (0)	18 (72)	38 (81)
Nausea	1 (17)	1 (17)	0 (0)	2 (33)	10 (53)	0 (0)	0 (0)	10 (53)	11 (44)	1 (4)	0 (0)	12 (48)	29 (62)
Dizziness	3 (50)	1 (17)	0 (0)	4 (67)	6 (32)	2 (11)	0 (0)	8 (42)	9 (36)	3 (12)	0 (0)	12 (48)	24 (51)
Arthralgia	0 (0)	3 (50)	0 (0)	4 (67)	4 (21)	2 (11)	0 (0)	6 (32)	4 (16)	5 (20)	0 (0)	10 (40)	17 (36)
Abdominal Pain	0 (0)	1 (17)	0 (0)	3 (50)	0 (0)	0 (0)	0 (0)	0 (0)	0 (0)	1 (4)	0 (0)	1 (4)	17(36)
Diarrhea	2 (33)	0 (0)	0 (0)	2 (33)	0 (0)	0 (0)	0 (0)	0 (0)	2 (8)	0 (0)	0 (0)	2 (8)	12 (26)
Vomiting	0 (0)	0 (0)	0 (0)	0 (0)	4 (21)	0 (0)	0 (0)	4 (21)	4 (16)	0 (0)	0 (0)	4 (16)	6 (13)
Shortness of Breath	1 (17)	0 (0)	0 (0)	1 (17)	2 (11)	0 (0)	0 (0)	2 (11)	3 (12)	0 (0)	0 (0)	3 (12)	ND
Change in Exercise Tolerance	2 (33)	0 (0)	0 (0)	2 (33)	2 (11)	1 (5)	0 (0)	3 (16)	4 (16)	1 (4)	0 (0)	5 (20)	ND
Chest Pain	0 (0)	0 (0)	0 (0)	0 (0)	3 (16)	0 (0)	0 (0)	3 (21)	3 (12)	0 (0)	0 (0)	3 (12)	ND
Urticaria	0 (0)	0 (0)	0 (0)	0 (0)	0 (0)	0 (0)	0 (0)	0 (0)	0 (0)	0 (0)	0 (0)	0 (0)	ND

ND = not determined.

Four of 25 participants (16%) bitten by 3 mosquitoes developed clinical symptoms at least possibly related to malaria exposure before or at the time of qPCR positivity. A total of 7 solicited symptoms were recorded with the most common being headache. In the time between positive malaria diagnosis by qPCR and microscopy (mean 79 hours), an additional 64 solicited symptoms at least possibly related to malaria exposure were recorded.

Laboratory abnormalities were noted in 20 of 25 (80%) participants challenged by the bite of 3 aseptically-reared mosquitoes, with mild aspartate aminotransferase (AST) and alanine aminotransferase (ALT) elevations being the most common (44% and 36%, respectively) (Table 4). Twelve (48%) participants experienced moderate or severe laboratory abnormalities following CHMI, and low platelet count was the only severe laboratory abnormality detected (16%). The lowest platelet counts in the 4 participants who experienced Grade 3 thrombocytopenia were 97, 89, 89, and 73×10^3^/µL. No hypoglycemic events were recorded. All laboratory abnormalities resolved after malaria treatment. All results of ECG testing and serum troponin levels were normal.

**Table 4:** Laboratory abnormalities recorded during malaria illness after receiving Plasmodium falciparum sporozoites by the bites of 3 aseptic mosquitoes.

		Study Group		
	Normal Range	Initial Study [22] (%)	Current Study (%)	Total (%)
**AST (IU/L)**				
None	0–40	4 (67)	9 (47)	13 (52)
Mild	41–99	2 (33)	9 (47)	11 (44)
Moderate	100–199	0 (0)	1 (5)	1 (4)
Severe	≥200	0 (0)	0 (0)	0 (0)
**ALT (IU/L)**				
None	0–55 ♂; 0–40 ♀	4 (67)	11 (58)	15 (60)
Mild	56–137 ♂; 41–99 ♀	2 (33)	7 (37)	9 (36)
Moderate	138–274 ♂; 100–199 ♀	0 (0)	1(5)	1 (4)
Severe	≥275 ♂; ≥200 ♀	0 (0)	0 (0)	0 (0)
**Hemoglobin (g/dL)**				
None	12.5–17.0 ♂; 11.5–15.0 ♀	6 (100)	19 (100)	25 (100)
Mild	10.6–12.4 ♂; 11.1–11.4 ♀	0 (0)	0 (0)	0 (0)
Moderate	10,0–10.5 ♂; 9.6–10.0 ♀	0 (0)	0 (0)	0 (0)
Severe	<10.0 ♂; ≤9.5 ♀	0 (0)	0 (0)	0 (0)
**WBC (× 10^3^/mm^3^)**				
None	4.0–10.5	3 (50)	12 (63)	15 (60)
Mild	2.5–3.9	2 (33)	5 (26)	7 (28)
Moderate	1.5–2.4	1 (17)	2 (11)	3 (12)
Severe	<1.5	0 (0)	0 (0)	0 (0)
**Platelets (× 10^3^/mm^3^)**				
None	≥140	2 (33)	10 (53)	12 (48)
Mild	125–139	0 (0)	3 (16)	3 (12)
Moderate	100–124	3 (50)	3 (16)	6 (24)
Severe*	20–99	1 (17)*	3 (16)*	4 (16)*

AST = aspartate aminotransferase; ALT = alanine aminotransferase; ♂ = male; ♀ = female.

* Thrombocytopenia was the only severe laboratory abnormality noted during the study.

## Discussion

The use of 3 Pf-infected, aseptically-reared *A. stephensi* mosquitoes bites safely and effectively transmitted malaria to all 25 participants (100%) tested in this and a previous study. Preliminary results from a previous smaller study had suggested that the 3 mosquito regimen was reliable and safe in 6 participants [22], and this has been confirmed in the current study with an additional 19 participants. Both groups experienced similar reactogenicity, developed parasitemia at similar levels and intervals, and had similar clinical symptoms, signs and laboratory abnormalities associated with malaria infection.

The use of 3 aseptically-raised mosquitoes bites for CHMI studies appears to be safe and well-tolerated. Symptoms and signs experienced by participants were generally mild or moderate, and the only severe symptom or sign experienced in the post-CHMI was fever >39.0°C (maximum 39.9°C) in 44% of participants. This is comparable to severe fever and other signs and symptoms seen in traditional 5 mosquito CHMI [30]. Symptoms associated with malaria illness seemed to peak on the day of microscopy diagnosis and the following day, likely the result of an inflammatory response triggered by patent blood stage infection and corresponding to the pyrogenic threshold described by Ross and Thomson [31] and/or the killing of parasites by chloroquine. Laboratory abnormalities were mostly mild and a few were moderate, with the exception of severe thrombocytopenia that was transient and not clinically apparent. All laboratory abnormalities are similar to those described in traditional 5 mosquito CHMI [30]. None of the 25 participants experienced severe malaria as defined by the WHO [32].

There has been controversy in the field as to whether the intensity of salivary gland infections with PfSPZ affects the infectivity and/or pre-patent period after CHMI. In our earlier study, 6 participants were exposed to a geometric mean total of 57,187 PfSPZ (range 18,000–112,000) corresponding to 16,645 PfSPZ/mosquito (range 2500–57,500). In the current study, 19 volunteers were exposed to more than 2 times as many total PfSPZ (geometric mean 132,269; range 48,000–240,000) and PfSPZ/mosquito (geometric mean 37,894; range 3500–152,000). Yet both studies achieved 100% infectivity and the pre-patent periods, incubation periods, and parasite density in blood by microscopy at time of diagnosis were similar. Thus, despite the presence of 2–3 times as many PfSPZ in the mosquito salivary glands, we did not see measureable differences in patency parameters, suggesting that similar numbers of PfSPZ were inoculated effectively into the volunteers. This may be because volunteers were exposed for a defined period of time (5 minutes) and based on the anatomy and diameter of the salivary duct, the PfSPZ generally move in single file down the duct and into the skin [33], thus limiting the numbers of PfSPZ inoculated. Even during our earlier study, the mosquitoes may have had significantly more PfSPZ/mosquitoes than in other studies [22]. Although there are limited data for comparison, since most other groups only report on PfSPZ/mosquito using a semi-quantitative glandular scale at which 4+ (>1000 PfSPZ/mosquito) is the most intensely infected, in many other studies mosquitoes had 3+ (101–1000 PfSPZ/mosquito), which is far below the density of PfSPZ/mosquito in our studies.

Compared to infectivity controls in a recent study of CHMI utilizing the traditional 5 mosquito methodology in malaria-naïve adults, the 3 mosquito aseptic CHMI resulted in detectable malaria patency by qPCR an average of 1.1 days earlier (Figure 1) with qPCR parasite densities measured as approximately 4 times higher. Increased sporozoite inoculation and liver burden could result in reduced pre-patent periods [34] or prolonged parasitemias as merozoites presumably continue to be released from the liver [35]. *A. stephensi* mosquitoes reared in standard insectaries are contaminated with bacteria and fungi, which may reduce mosquito fitness and infection by the malaria parasite [36]–[38]. The high sporozoite load carried by the aseptically-reared mosquitoes may partially explain the reduced pre-patent period compared to the traditional challenge model. However, sporozoite injection in *P. yoelii* has proven to be highly variable, ranging from 0–1,297 per bite (mean 123) and is only weakly correlated to sporozoite gland quantity [39]. Moreover, the total sporozoite salivary gland load measured in the 3 mosquito population was less in our prior study compared to the current study (57,187 vs. 132,269) with no discernible difference in pre-patent periods. The goal of CHMI is to expose volunteers to the optimal quantity of mosquito bites necessary to reliably induce infection without overwhelming the immune system or the vaccine-induced protection mediated by a study intervention. These findings suggest an advantage to the use of the 3 mosquito aseptic CHMI model in place of the traditional 5 mosquito bite model.

If these earlier results are borne out in further studies, the predictable pre-patent periods, earlier diagnosis of malaria and earlier antimalarial treatment administration of aseptic 3 mosquito CHMI will allow termination of the infection sooner than in the traditional model. As more experience is gained in conducting CHMI trials the use of qPCR to achieve earlier diagnoses is attractive, but it also carries risks. A false negative or false positive result in a small challenge trial of a malaria vaccine could profoundly alter study results. Moreover, the presence of Pf DNA does not necessarily prove the presence of live, blood-stage asexual parasites. Although DNA fragments are quickly eliminated from the bloodstream, making it less likely that PCR would detect dead parasite forms [40], qPCR Pf DNA methods also identify the post-treatment appearance of gametocytes [41]. Gametocyte development is delayed relative to asexual Pf forms [42]–[44] and gametocytes are resistant to most of the antimalarial drugs. This suggests that while pre-patency qPCR is highly likely to represent incipient microscopy-positive blood-stage infection, post-treatment qPCR may be of questionable value in the evaluation of patent asexual parasitemia, and should be interpreted with caution. Mounting experience with qPCR methods during CHMI may allow for this technique to be considered as an alternative method for malaria diagnosis under controlled conditions [45]–[47]. However, this will require rigorous measures to standardize and eventually validate the assay. Additionally, the use of qPCR testing as a basis for diagnosis and treatment would preclude analysis of parasite multiplication rates and estimation of liver loads, but the benefit of early treatment of participants in abrogating clinical symptoms should be weighed against the need for data collection beyond protection against patent parasitemia.

The accuracy of microscopy-confirmed diagnosed malaria in parallel with qPCR diagnosis has been studied in the context of CHMI by mosquito bite [27], [48]–[54]. Among these 8 reports, a total of 63 non-vaccinated “controls” were followed for development of parasitemia by microscopy before treatment. For these 63 controls, the average time to positive parasitemia by microscopy was 9.9 days compared to 7.2 days by qPCR. None of these studies reported a positive qPCR that did not later turn microscopy-positive, and none reported a false negative result. These observations are consistent with our finding that qPCR-confirmed malaria patency occurred ∼3 days before microscopy-confirmed malaria patency. If CHMI participants were to be treated based on patency by qPCR rather than microscopy, this would presumably mitigate the risks of malaria symptoms while maintaining a high diagnostic standard of high sensitivity and 100% specificity in CHMI by mosquito challenge studies to date.

We report, herein, successful application of an aseptic CHMI technique and 100% malaria infectivity using 3, rather than 5, mosquitoes in a CHMI study. Although tested in only 25 participants to date, the 5 mosquito CHMI was introduced as the standard after assessment in only 6 volunteers [1]. Fewer mosquitoes required would streamline the CHMI process by reducing resources needed to produce, infect, transport and store more mosquitoes; the potential local reactogenicity experienced by CHMI participants at mosquito bite sites; and the theoretical risk of infective mosquito escape to the environment. Future studies could easily confirm this model by including both the 5 mosquito traditional CHMI model and the 3 aseptic mosquito model for infectivity controls in parallel. More importantly, this is the next step toward developing and evaluating the infectivity of aseptic, purified, cryopreserved Pf sporozoites administered by needle and syringe to infect volunteers rather than relying on the bite of a mosquito [55]. This would allow institutions without the capability of rearing infectious mosquitoes to safely, and reliably conduct malaria challenge trials, and such studies are currently underway.

### Conclusions

The novel use of 3 aseptically reared mosquitoes is a safe and effective means to transmit malaria infection to malaria-naïve adults in a controlled setting. Confirmatory studies would potentially set a new standard for evaluation of malaria vaccines and antimalarial drugs in non-endemic areas using this model. Microscopy continues to be the gold standard used for the diagnosis of malaria in centers conducting CHMI, but qPCR diagnoses parasitemia several days earlier than thick blood smear. Further work to establish the specificity, practicality, rapidity, and standardization of qPCR should be considered with the intent of eventually transitioning from thick blood smear to qPCR for diagnosis in CHMI.

## Supporting Information

Figure S1 **Participant Flow Diagram.**
(TIFF)

Table S1 **Quantitative Polymerase Chain Reaction Results.**
(DOCX)

Checklist S1 **CONSORT checklist.**
(DOC)

Protocol S1 **Study Protocol.**
(DOC)
