# Supplementary figures and images for: Successful Human Infection with P. falciparum Using Three Aseptic Anopheles stephensi Mosquitoes: A New Model for Controlled Human Malaria Infection
Source: PLoS One. 2013 Jul 16;8(7):e68969. doi: 10.1371/journal.pone.0068969 (PMC3712927; doi:10.1371/journal.pone.0068969)

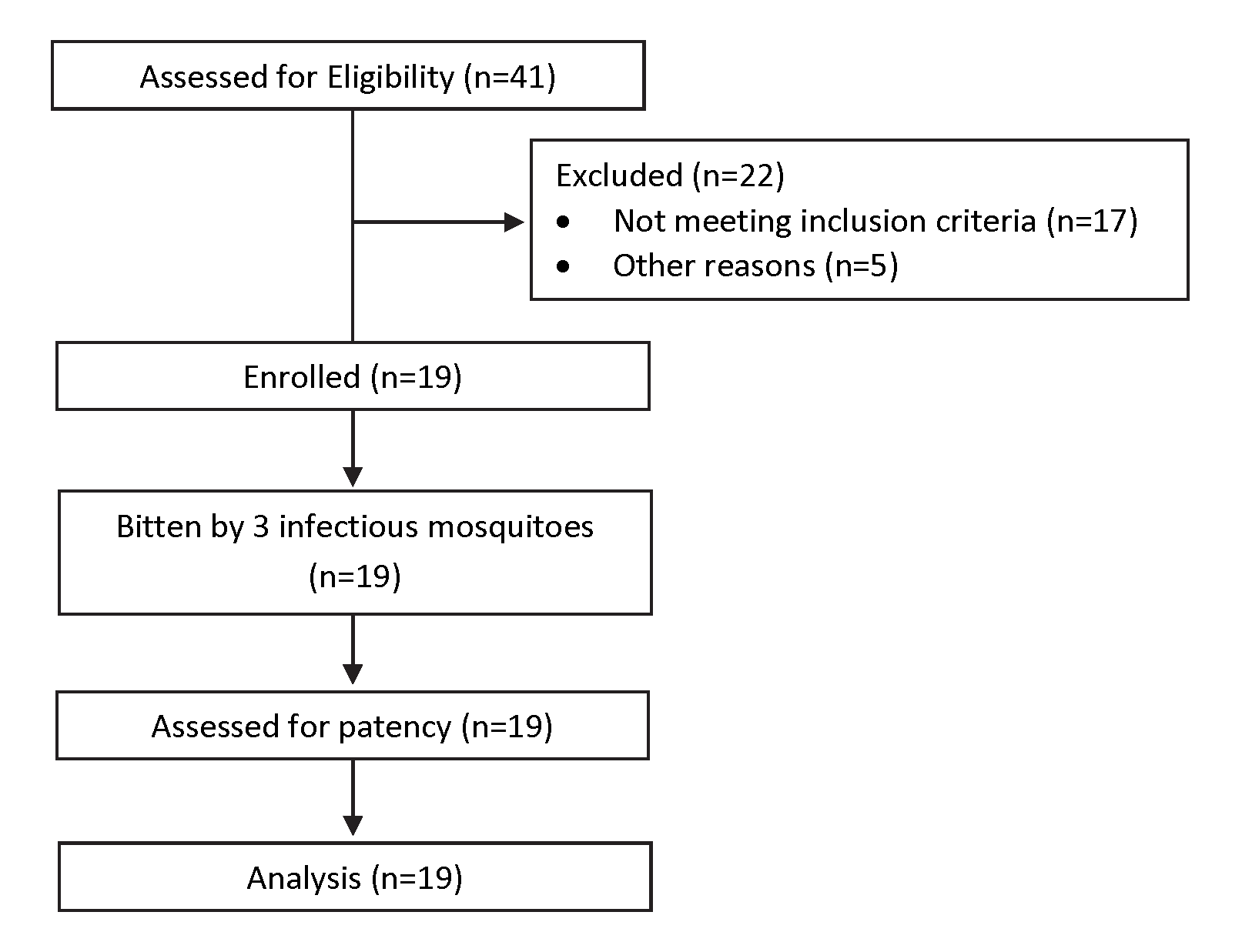

Supplement: Figure S1 — Participant Flow Diagram. (TIFF) [file pone.0068969.s001.tiff]
