# Supplementary material for: Successful Human Infection with P. falciparum Using Three Aseptic Anopheles stephensi Mosquitoes: A New Model for Controlled Human Malaria Infection
Source: PLoS One. 2013 Jul 16;8(7):e68969. doi: 10.1371/journal.pone.0068969 (PMC3712927; doi:10.1371/journal.pone.0068969)
Supplement: Table S1 — Quantitative Polymerase Chain Reaction Results. (DOCX) [file pone.0068969.s002.docx]

**Table S1.** Quantitative Polymerase Chain Reaction (PCR) Results. All quantitative PCR data measured in parasites per milliliter obtained in participants to date receiving *Plasmodium falciparum* sporozoites by the bites of 3 aseptic mosquitoes at the University of Maryland School of Medicine.

| **Study** | **Participant** | **Day 7** | **Day 8** | **Day 9** | **Day 10** | **Day 11** | **Day 12** | **Day 13** | **Day 14** | **Day 15** | **Day 16** |
| --- | --- | --- | --- | --- | --- | --- | --- | --- | --- | --- | --- |
| Previous [22] |  |  |  |  |  |  |  |  |  |  |  |
|  | 8 | 37 | 122 | 334 | 271 | **13,619** | 423 | 0 | 0 | 0 | not done |
|  | 9 | 0 | 42 | 245 | 0 | **2,379** | 49 | 0 | 0 | 0 | not done |
|  | 13 | 4 | 529 | 675 | **461** | 4,472 | 166 | 0 | 0 | 0 | not done |
|  | 14 | 83 | 109 | 381 | 170 | **7,748** | 226 | 0 | 0 | 0 | not done |
|  | 17 | 91 | 158 | 347 | 324 | **24,860** | 370 | 0 | 0 | 0 | not done |
|  | 29 | 116 | 0 | 928 | 53 | **11,940** | 3,581 | 367 | 55 | 0 | not done |
| Current |  |  |  |  |  |  |  |  |  |  |  |
|  | 39 | 2,974 | 221 | not done | not done | not done | **370,254** | 32,127 | 531 | not done | 60 |
|  | 44 | 0 | 726 | 20,301 | 3,934 | **31,045** | 8,703 | 20 | 0 | 0 | not done |
|  | 45 | 10,654 | 396 | 6,736 | 1,626 | **8** | 161,169 | 0 | 19 | 23 | not done |
|  | 47 | 1,841 | 126 | 2,740 | 606 | **3,473** | 1,633 | 148 | 0 | not done | not done |
|  | 48 | 1,930 | 298 | **16,100** | 307 | 0 | 0 | not done | not done | not done | not done |
|  | 49 | 0 | 136 | 1,458 | 338 | **7** | 3,778 | 3 | 32 | not done | not done |
|  | 50 | 1,488 | 47 | 1,991 | 1,056 | **5,667** | 30,857 | 745 | 24 | not done | not done |
|  | 53 | 3,946 | 289 | 2,894 | 406 | **3,844** | 4,928 | 295 | 0 | not done | not done |
|  | 54 | 0 | 185 | 4,270 | 1,459 | **64** | 2,982 | 0 | 0 | not done | not done |
|  | 55 | 826 | 396 | 6,502 | 1,724 | **9,715** | 103,961 | 4,425 | 108 | not done | not done |
|  | 57 | 0 | 112 | 2,282 | 365 | **6,031** | 1,344 | 47 | 0 | not done | not done |
|  | 59 | 1,802 | not done | **26,489** | 1,809 | 22 | 0 | not done | 0 | not done | not done |
|  | 61 | 1,005 | 821 | 17,981 | **46,839** | 33,992 | 2,664 | 4,182 | 50 | not done | not done |
|  | 64 | 18,213 | 592 | **11,045** | 750 | 0 | 0 | 0 | 64 | not done | not done |
|  | 66 | 2,316 | 61 | 877 | 550 | 11,073 | **819** | 65,730 | not done | 27 | not done |
|  | 68 | 5,341 | 257 | 2,976 | 843 | **20,732** | 19,864 | 311 | 0 | not done | not done |
|  | 69 | 6,371 | 101 | 7,680 | 1,082 | **62,044** | 2,244 | 388 | 0 | not done | not done |
|  | 71 | 8,489 | 172 | 5,334 | 334 | **33,204** | 2,226 | 332 | 0 | not done | not done |
|  | 74 | 0 | 0 | 32 | 0 | 1 | 614 | 1,414 | **31,590** | 566 | 33 |

**Bolded and underlined** values denote the day of diagnosis by microscopy when treatment was initiated.
