# Supplementary material for: Successful Human Infection with P. falciparum Using Three Aseptic Anopheles stephensi Mosquitoes: A New Model for Controlled Human Malaria Infection
Source: PLoS One. 2013 Jul 16;8(7):e68969. doi: 10.1371/journal.pone.0068969 (PMC3712927; doi:10.1371/journal.pone.0068969)
Supplement: Protocol S1 — Study Protocol. (DOC) [file pone.0068969.s004.doc]

# **An Interventional *Plasmodium falciparum* Malaria Challenge Model Utilizing the NF54 Strain of Parasite Transmitted by Aseptic *A. stephensi* Mosquitoes to Healthy Malaria-Naïve Adult Volunteers**

# **DMID Protocol Number: 05-0053**

# **DMID Funding Mechanism:** VTEU Contract support N01-AI-25461

# **Pharmaceutical Support Provided by:** Sanaria, Inc.

# **Other Identifying Numbers:** H:29924; Malaria CVD 17000

# **IND Sponsor:** DMID

# **Principal Investigator:** Kirsten E. Lyke, MD

# **DMID Protocol Champion:** Steven R. Rosenthal, MD, MPH

# **DMID Medical Monitor:** Mirjana Nesin, M.D.

# **Draft or Version Number:** 4.0

# **Day Month Year** 12 May 2010

Statement of Compliance

The study will be carried out in accordance with Good Clinical Practice (GCP) as required by the following:

- United States (US) Code of Federal Regulations (CFR) applicable to clinical studies (45 CFR Part 46; 21 CFR Part 50, 21 CFR Part 56, and 21 CFR Part 312)
- ICH E6; 62 Federal Register 25691 (1997)
- NIH Clinical Terms of Award

All key personnel (all individuals responsible for the design and conduct of this study) have completed Human Subjects Protection Training.

*Refer to: [http://www.hhs.gov/ohrp/humansubjects/guidance/45cfr46.htm#46.](http://www.hhs.gov/ohrp/humansubjects/guidance/45cfr46.htm#46)*

[*http://www.fda.gov/cder/guidance/959fnl.pdf*](http://www.fda.gov/cder/guidance/959fnl.pdf)

[*http://grants.nih.gov/grants/guide/notice-files/NOT-OD-01-061.html*](http://grants.nih.gov/grants/guide/notice-files/NOT-OD-01-061.html)

[*http://www.cancer.gov/clinicaltrials/learning/page3*](http://www.cancer.gov/clinicaltrials/learning/page3)

Signature Page

The signature below constitutes the approval of this protocol and the attachments, and provides the necessary assurances that this trial will be conducted according to all stipulations of the protocol, including all statements regarding confidentiality, and according to local legal and regulatory requirements and applicable US federal regulations and ICH guidelines.

| Principal Investigator: | | | |
| --- | --- | --- | --- |
| Signed: |  | Date: |  |
|  | *Name* Kirsten E. Lyke, M.D.  *Title* Assistant Professor of Medicine |  |  |

Table of Contents

Page

[Statement of Compliance ii](#__RefHeading___Toc223229433)

[Signature Page iii](#__RefHeading___Toc223229434)

[Table of Contents iv](#__RefHeading___Toc223229435)

[List of Abbreviations vii](#__RefHeading___Toc223229436)

[Protocol Summary ix](#__RefHeading___Toc223229437)

[1 Key Roles 12](#__RefHeading___Toc223229438)

[2 Background Information and Scientific Rationale 16](#__RefHeading___Toc223229439)

[2.1 Background Information 16](#__RefHeading___Toc223229440)

[2.1.1 Background and Rationale of Malaria Challenge Studies 16](#__RefHeading___Toc223229441)

[2.1.2 Validation of Malaria Challenge Model Using Aseptic Mosquitoes 18](#__RefHeading___Toc223229442)

[2.1.3 Development and Validation of Molecular Diagnostic Methods 19](#__RefHeading___Toc223229443)

[2.2 Rationale 19](#__RefHeading___Toc223229444)

[2.3 Potential Risks and Benefits 20](#__RefHeading___Toc223229445)

[2.3.1 Potential Risks 20](#__RefHeading___Toc223229446)

[2.3.2 Known Potential Benefits 24](#__RefHeading___Toc223229447)

[3 Objectives 25](#__RefHeading___Toc223229448)

[3.1 Study Objectives 25](#__RefHeading___Toc223229449)

[3.1.1 Overall study objective 25](#__RefHeading___Toc223229450)

[3.1.2 Primary objective 25](#__RefHeading___Toc223229451)

[3.1.3 Secondary objectives 25](#__RefHeading___Toc223229452)

[3.2 Study Outcome Measures 26](#__RefHeading___Toc223229453)

[3.2.1 Primary Outcome Measures 26](#__RefHeading___Toc223229454)

[3.2.2 Secondary Outcome Measures 26](#__RefHeading___Toc223229455)

[4 Study Design 27](#__RefHeading___Toc223229456)

[4.1 Substudies (Real time PCR analysis) 29](#__RefHeading___Toc223229457)

[5 Study Enrollment and Withdrawal 30](#__RefHeading___Toc223229458)

[5.1 Subject Inclusion Criteria 31](#__RefHeading___Toc223229459)

[5.2 Subject Exclusion Criteria 31](#__RefHeading___Toc223229460)

[5.3 Treatment Assignment Procedures 33](#__RefHeading___Toc223229461)

[5.3.1 Randomization Procedures 33](#__RefHeading___Toc223229462)

[5.3.2 Masking Procedures 34](#__RefHeading___Toc223229463)

[5.3.3 Reasons for Withdrawal/Discontinuation 35](#__RefHeading___Toc223229464)

[5.3.4 Handling of Withdrawals 36](#__RefHeading___Toc223229465)

[5.3.5 Termination of Study 37](#__RefHeading___Toc223229466)

[6 Study Intervention/Investigational Product 38](#__RefHeading___Toc223229467)

[6.1 Study Product Description 38](#__RefHeading___Toc223229468)

[6.1.1 Acquisition 38](#__RefHeading___Toc223229469)

[6.1.2 Formulation, Packaging, and Labeling 38](#__RefHeading___Toc223229470)

[6.1.3 Product Storage and Stability 39](#__RefHeading___Toc223229471)

[6.2 Dosage, Preparation and Administration of Study Intervention/Investigational Product 39](#__RefHeading___Toc223229472)

[6.3 Modification of Study Intervention/Investigational Product for a Participant 40](#__RefHeading___Toc223229473)

[6.4 Accountability Procedures for the Study Intervention/Investigational Product 41](#__RefHeading___Toc223229474)

[6.5 Assessment of Subject Compliance with Study Intervention/Investigational Product 41](#__RefHeading___Toc223229475)

[6.6 Concomitant Medications/Treatments 41](#__RefHeading___Toc223229476)

[7 Study Schedule 43](#__RefHeading___Toc223229477)

[7.1 Screening 43](#__RefHeading___Toc223229478)

[7.2 Enrollment/Baseline 44](#__RefHeading___Toc223229479)

[7.2.1 Malaria Challenge 45](#__RefHeading___Toc223229480)

[7.3 Follow-up and Surveillance 45](#__RefHeading___Toc223229481)

[7.3.1 Malaria Infection 47](#__RefHeading___Toc223229482)

[7.4 Final Study Visit 48](#__RefHeading___Toc223229483)

[7.5 Early Termination Visit 49](#__RefHeading___Toc223229484)

[7.6 Unscheduled Visit 49](#__RefHeading___Toc223229485)

[8 Study Procedures/Evaluations 51](#__RefHeading___Toc223229486)

[8.1 Clinical Evaluations 51](#__RefHeading___Toc223229487)

[8.1.1 Screening and enrollment: 51](#__RefHeading___Toc223229488)

[8.1.2 Subject follow-up: 53](#__RefHeading___Toc223229489)

[8.2 Laboratory Evaluations 57](#__RefHeading___Toc223229490)

[8.2.1 Clinical Laboratory Evaluations 57](#__RefHeading___Toc223229491)

[8.2.2 Special Assays or Procedures 60](#__RefHeading___Toc223229492)

[8.2.3 Specimen Preparation, Handling, and Shipping 61](#__RefHeading___Toc223229493)

[9 Assessment of Safety 63](#__RefHeading___Toc223229494)

[9.1 Specification of Safety Parameters 63](#__RefHeading___Toc223229495)

[9.2 Methods and Timing for Assessing, Recording, and Analyzing Safety Parameters 63](#__RefHeading___Toc223229496)

[9.2.1 Adverse Events 63](#__RefHeading___Toc223229497)

[9.2.2 Intensity of Response to the Malaria Challenge and to a Malaria Event 65](#__RefHeading___Toc223229498)

[9.2.3 Serious Adverse Events 70](#__RefHeading___Toc223229499)

[9.2.4 Procedures to be Followed in the Event of Abnormal Laboratory Test Values or Abnormal Clinical Findings 71](#__RefHeading___Toc223229500)

[9.3 Adverse Event Reporting Procedures 73](#__RefHeading___Toc223229501)

[9.3.1 Serious Adverse Events 73](#__RefHeading___Toc223229502)

[9.3.2 Regulatory Reporting for Studies Conducted Under DMID‑Sponsored IND 74](#__RefHeading___Toc223229503)

[9.3.3 Regulatory Reporting for Studies Not Conducted Under DMID‑Sponsored IND 74](#__RefHeading___Toc223229504)

[9.3.4 Other Adverse Events (if applicable) 74](#__RefHeading___Toc223229505)

[9.3.5 Reporting of Pregnancy 74](#__RefHeading___Toc223229506)

[9.4 Halting Rules 74](#__RefHeading___Toc223229507)

[9.5 Safety Oversight (ISM plus SMC) 76](#__RefHeading___Toc223229508)

[9.5.1 Independent Safety Monitor 76](#__RefHeading___Toc223229509)

[9.5.2 Safety Monitoring Committee 77](#__RefHeading___Toc223229510)

[10 Clinical Monitoring 78](#__RefHeading___Toc223229511)

[10.1 Site Monitoring Plan 78](#__RefHeading___Toc223229512)

[11 Statistical Considerations 79](#__RefHeading___Toc223229513)

[11.1 Overview of Study Hypotheses 79](#__RefHeading___Toc223229514)

[11.2 Sample Size Considerations 80](#__RefHeading___Toc223229515)

[11.3 Statistical Considerations 81](#__RefHeading___Toc223229516)

[11.4 Planned Interim Analyses 81](#__RefHeading___Toc223229517)

[11.4.1 Safety Review 82](#__RefHeading___Toc223229518)

[11.4.2 Efficacy Review 82](#__RefHeading___Toc223229519)

[11.5 Final Analysis Plan 83](#__RefHeading___Toc223229520)

[12 Source Documents and Access to Source Data/Documents 84](#__RefHeading___Toc223229521)

[13 Quality Control and Quality Assurance 85](#__RefHeading___Toc223229522)

[14 Ethics/Protection of Human Subjects 86](#__RefHeading___Toc223229523)

[14.1 Ethical Standard 86](#__RefHeading___Toc223229524)

[14.2 Institutional Review Board 86](#__RefHeading___Toc223229525)

[14.3 Informed Consent Process 86](#__RefHeading___Toc223229526)

[14.3.1 Informed Consent/Assent Process (in Case of a Minor) 87](#__RefHeading___Toc223229527)

[14.4 Exclusion of Women, Minorities, and Children (Special Populations) 87](#__RefHeading___Toc223229528)

[14.5 Subject Confidentiality 87](#__RefHeading___Toc223229529)

[14.6 Study Discontinuation 88](#__RefHeading___Toc223229530)

[14.7 Future Use of Stored Specimens 88](#__RefHeading___Toc223229531)

[14.8 Compensation 89](#__RefHeading___Toc223229532)

[15 Data Handling and Record Keeping 90](#__RefHeading___Toc223229533)

[15.1 Data Management Responsibilities 90](#__RefHeading___Toc223229534)

[15.2 Data Capture Methods 90](#__RefHeading___Toc223229535)

[15.3 Types of Data 91](#__RefHeading___Toc223229536)

[15.4 Timing/Reports 91](#__RefHeading___Toc223229537)

[15.5 Study Records Retention 91](#__RefHeading___Toc223229538)

[15.6 Protocol Deviations 91](#__RefHeading___Toc223229539)

[16 Publication Policy 93](#__RefHeading___Toc223229540)

List of Abbreviations

| °C | Degrees Celsius |
| --- | --- |
| *A. stephensi* | *Anopheles stephensi* |
| AE | Adverse Event/Adverse Experience |
| ALT | Alanine Aminotransferase (same as SGPT) |
| AST | Aspartate aminotransferase (same as SGOT) |
| CBC | Complete Blood Count |
| CFR | Code of Federal Regulations |
| cGMPs | current Good Manufacturing Practices |
| CRF | Case Report Form |
| CVD | Center for Vaccine Development |
| DCC | Data Coordinating Center |
| DMID | Division of Microbiology and Infectious Diseases, NIAID, NIH, DHHS |
| DSMB | Data and Safety Monitoring Board |
| eCRF | Electronic Case Report Form |
| FDA | Food and Drug Administration |
| GCRC | General Clinical Research Center |
| GCP | Good Clinical Practice |
| HIPAA | Health Insurance Portability and Accountability Act |
| HIV | Human Immunodeficiency Virus |
| IB | Investigator’s Brochure |
| ICH | International Conference for Harmonization |
| ID_100_ | 100% Infective Dose |
| IND | Investigational New Drug Application |
| IRB | Institutional Review Board |
| ISM | Independent Safety Monitor |
| MedDRA® | Medical Dictionary for Regulatory Affairs |
| MOP | Manual of Procedures |
| μL | Microliter |
| mL | Milliliter |
| N | Number (typically refers to subjects) |
| NMRC | Naval Medical Research Center |
| NIAID | National Institute of Allergy and Infectious Diseases, NIH, DHHS |
| NIH | National Institutes of Health |
| PCR | Polymerase Chain Reaction |
| PHI | Protected Health Information |
| *Pf* | *Plasmodium falciparum* |
| PI | Principal Investigator |
| PBMC | Peripheral Blood Mononuclear Cell |
| QA | Quality Assurance |
| QC | Quality Control |
| RBCs | Red blood cells/Red blood cell count |
| RNA | Ribosomal nucleic acid |
| RTQ-PCR | Real-time Quantitative PCR |
| RUNMC | Radboud University Nijmegen Medical Center |
| SAE | Serious Adverse Event/Serious Adverse Experience |
| SMC | Safety Monitoring Committee |
| SNBL | Shin Nippon Biomedical Laboratories |
| SOP | Standard Operating Procedure |
| UMMS | University of Maryland Medical Systems |
| US | United States |
| USAMRIID | The United States Army Medical Research Institute for Infectious Diseases |
| USP | United States Pharmacopeia |
| VTEU | Vaccine and Treatment Evaluation Unit |
| WBC | White blood cells (leukocytes)/White blood cell count |
| WHO | World Health Association |
| WRAIR | Walter Reed Army Institute of Research |

Protocol Summary

| **Title:** | An Interventional *Plasmodium falciparum* Malaria Challenge Model Utilizing the NF54 Strain of Parasite Transmitted by Aseptic *A. stephensi* Mosquitoes to Healthy Malaria-Naïve Adult Volunteers |
| --- | --- |
| **Phase:** | Phase 1 |
| **Population:** | 38 healthy male and female subjects aged 18 to 40 years, inclusive, from the greater Baltimore community of the United States (US) |
| **Number of Sites:** | 1 site: The University of Maryland, Center for Vaccine Development |
| **Study Duration:** | 16 months |
| **Subject Participation Duration:** | 56 days – active participation  10 months – passive surveillance |
| **Description of Agent or Intervention:** | Aseptic *Anopheles stephensi* mosquitoes infected with *P. falciparum* NF54 strain sporozoites by membrane feeding upon erythrocytes infected with *P. falciparum* gametocytes. |
| **Objectives:** | Primary:   1. Develop and evaluate the safety and tolerability of a new human malaria challenge model using aseptically-raised anopheles mosquitoes infected with the NF54 isolate of *P. falciparum* and reared under current Good Manufacturing Practices (cGMPs) conditions   Secondary:   1. Obtain information on the minimum number of *A. stephensi* bites required to safely achieve 100% adult human volunteer infectivity (Malaria challenge, Part A). 2. Obtain information on the minimum quantity of *A. stephensi* bites in a second challenge study to achieve 100% adult human volunteer infectivity (Malaria challenge, Part B). 3. Develop molecular diagnostic techniques for rapid and accurate real-time diagnosis of *P. falciparum* infection, to assess the role as a new diagnostic standard for *P. falciparum* challenge studies. |
| **Description of Study Design:** | This is a randomized Phase 1 trial of the aseptically raised anopheles mosquito malaria challenge. Adults aged 18-40 years will be randomized to one group of 18 subjects (Part A) or a second group of 20 subjects (Part B). Adults will be randomized to receive 1, 3 or 5 bites* of *A. stephensi* mosquitoes infected with the NF54 strain of chloroquine-sensitive *P. falciparum*. Part B will be informed by the results of Part A. Thus a total of ~38 adults will receive a malaria challenge. The challenge for Part A will be given on day 0 with the subsequent group (Part B) commencing after a 56 day safety review. Solicited adverse events will be recorded on the days of malaria challenge, outpatient days 5-7, 19-28, 35, 42, 49, and 56 post-challenge event. Inpatient analysis will occur from Days 8-18 or until three-day directly observed therapy for *P. falciparum* infection is complete. Additional outpatient, post-malaria infection follow-up will occur weekly for 4 weeks. Unsolicited adverse events will be recorded for 56 days after each malaria challenge event. Participants will receive a telephone follow-up six and twelve months after enrollment. *In the event that less than 18 volunteers are recruited for Part A, the 1 bite experimental cohort arm will be dropped. If less than 12 volunteers are recruited, the volunteers will not proceed with challenge. |
| **Estimated Time to Complete Enrollment:** | 12 weeks (8 weeks for Part A and 4 weeks for Part B, performed sequentially) |

**Schematic of Study Design**:

Prior to

Total 38: Obtain informed consent. Screen subjects by criteria; obtain history document.

Enrollment

Randomize

**Malaria**

**Challenge**

**Part A**

Perform pregnancy test; collect blood for assays;

**Perform Malaria Challenge**

*The 1 bite cohort

will be dropped if

recruitment results

in 12-17 volunteers

Clinical and AE assessment. Outpatient evaluation Days 0-7 and Days 19-28

Inpatient evaluation Days 8-18

for Part A.

Clinical and AE assessment, Outpatient evaluation Days 29-56

SMC Review

Day 56 Safety

Data. Part A.

**Assessment of Study Outcome Measure (ID_100_)**

**Malaria**

**Challenge**

Perform pregnancy test; collect blood for assays;

**Perform Malaria Challenge**

**Part B**

Clinical and AE assessment: Outpatient evaluation Days 0-7 and Days 19-28

Inpatient evaluation Days 8-18

**Reassess Study Outcome Measure (ID_100_)**

Clinical and AE assessment, Outpatient evaluation Days 29-56

SMC Review

Day 56 Safety

Data. Part B.

# Key Roles

| **Individuals:** |  |
| --- | --- |
|  | **Protocol Champion:**  Steven R. Rosenthal, MD, MPH  Medical Officer, Malaria Vaccine Development Section  Parasitology & International Programs Branch (HNM53)  Division of Microbiology & Infectious Diseases  National Institute of Allergy & Infectious Diseases  National Institutes of Health  6610 Rockledge Drive, Rm. 5067  Bethesda, MD 20892, USA  Tel: 301-496-2544  Fax: 301-402-0659  E-mail: rosenthals@niaid.nih.gov |
|  | **Principal Investigator:**  Kirsten E. Lyke, MD  Assistant Professor of Medicine  University of Maryland School of Medicine  Center for Vaccine Development  Division of Geographic Medicine  Health Sciences Facility Room 480  685 West Baltimore Street  Baltimore, Maryland 21201-1509  Tel: 410 706 7376  Fax: 410-706-6205  E-mail: [Klyke@medicine.umaryland.edu](mailto:Klyke@medicine.umaryland.edu) |
|  | **Associate Investigators:**  ***University of Maryland***  Robert Edelman, MD, FACP, Professor of Medicine and Pediatrics, Associate Director for Clinical Research, Phone: 410-706-8443 E-mail: [Redelman@medicine.umaryland.edu](mailto:Redelman@medicine.umaryland.edu)  Christopher V. Plowe, MD, MPH, Professor of Medicine, Chief, Malaria Division, Phone: 410-706-2491, E-mail: [Cplowe@medicine.umaryland.edu](mailto:Cplowe@medicine.umaryland.edu)  Matthew Laurens, MD, MPH, Research Associate, Pediatric Infectious Diseases  Phone: 410-706-7376, E-mail: [MLaurens@medicine.umaryland.edu](mailto:MLaurens@medicine.umaryland.edu)  Andrea Berry, MD, Fellow, Pediatric Infectious Diseases  Phone: 410-706-7376, E-mail: [ABerry@medicine.umaryland.edu](mailto:ABerry@medicine.umaryland.edu)  Miriam Laufer, MD, MPH, Assistant Professor of Pediatrics,  Phone: 410-706-7376, E-mail :  [MLaufer@medicine.umaryland.edu](mailto:MLaufer@medicine.umaryland.edu)  Mark Travassos, MD, Fellow, Pediatric Infectious Diseases  Phone: 410-706-7376, E-mail:  MTravass@medicine.umaryland.edu  William Blackwelder, MD, Professor, Biostatistical consultant, Phone: 410-706-7376, (301) 564-6137, E-mail: [wcb@boo.net](mailto:wcb@boo.net)  Marcelo B. Sztein, MD, Professor of Pediatrics, Medicine  and Microbiology and Immunology, Chief, Cellular Immunology Division, Phone: 410-706-2345, E-mail: [MSztein@medicine.umaryland.edu](mailto:MSztein@medicine.umaryland.edu)  Christopher Defilippi, MD, Associate Professor of Medicine, Division of Cardiology  Phone: 410-328-6072 , E-mail:  CDefilip@medicine.umaryland.edu  ***Sanaria, Inc.***  Stephen L. Hoffman, MD, Chief Executive and Scientific Officer, 9800 Medical Center Drive, Ste A209  Rockville, MD 20850  Tel: 301-770-3222  Fax : 301-770-5554  E-mail: slhoffman@sanaria.com  Kim Lee Sim, PhD, Vice President, Process Development and Manufacturing,  9800 Medical Center Drive, Ste A209  Rockville, MD 20850  Tel: 301-770-3222  E-mail: [ksim@sanaria.com](mailto:ksim@sanaria.com)  Adam Richman, PhD, Senior Scientist, Entomology,  9800 Medical Center Drive, Ste A209  Rockville, MD 20850  Tel: 301-770-3222  E-mail: [arichman@sanaria.com](mailto:arichman@sanaria.com)  **DMID Regulatory Affairs Specialist**  Blossom Smith  Office of Regulatory Affairs  National Institute of Allergy & Infectious Diseases  National Institutes of Health  Tel: 301-402-9564  E-mail: smithbl@niaid.nih.gov  **DMID Medical Monitor**  Mirjana Nesin, MD  Office of Clinical Research Affairs (OCRA)  Division of Microbiology & Infectious Diseases  National Institute of Allergy & Infectious Diseases  National Institutes of Health  6610 Rockledge Drive  Bethesda, MD 20892, USA  Tel: (301) 496 7067  E-mail: [nesinm@niaid.nih.gov](mailto:nesinm@niaid.nih.gov)  **UMD Independent Safety Monitor:**  Alan S. Cross, MD  Professor of Medicine  University of Maryland School of Medicine  Center for Vaccine Development  Division of Geographic Medicine  Health Sciences Facility Room 480  685 West Baltimore Street  Baltimore, Maryland 21201-1509  Tel: 410 706 7376  Fax: 410-706-6205  E-mail: [ACross@medicine.umaryland.edu](mailto:ACross@medicine.umaryland.edu) |
| **Institutions:** |  |
|  | **University of Maryland School of Medicine**  Center for Vaccine Development Outpatient Facility  685 West Baltimore Street, HSF I, 4^th^ floor  Baltimore, Maryland 21201-1509  Contact: ***Kirsten E. Lyke, MD,*** *Assistant Professor of Medicine*  Tel: 410 706 7376  Fax: 410-706-6205  E-mail: [Klyke@medicine.umaryland.edu](mailto:Klyke@medicine.umaryland.edu)  **Sanaria, Inc.**  9800 Medical Center Drive, Ste A209  Rockville, MD 20850  *Contact :* Stephen L. Hoffman, MD, *Chief Executive and Scientific Officer*  Tel: 301-770-3222  Fax : 301-770-5554  E-mail: slhoffman@sanaria.com  **The EMMES Corporation**  (NIH contractor for data management and statistical support)  401 N. Washington St., Suite 700  Rockville, MD 20850  Tel: 301-251-1161, Fax: 301-251-1355  Email: [malaria@emmes.com](mailto:malaria@emmes.com) |
| **Optional:** | **Inpatient Facility**  General Clinical Research Center  University of Maryland Medical Center  22 South Greene Street, 10th floor  Baltimore, MD 21201-1730  Tel: 410-328-7369  Fax: 410-328-8749  E-mail: [gcrc@medicine.umaryland.edu](mailto:gcrc@medicine.umaryland.edu)  Or as back-up facility:  Shin Nippon Biomedical Laboratories Clinical Pharmacology Laboratory (SNBL-CPC) at the University of Maryland BioPark Facility  800 West Baltimore St., 5th Floor  Baltimore, MD 21201  Tel: 410-706-8799 Contact: Toni Tiburzi  <http://www.snbl-cpc.com/pdf/101805Press.pdf> |

# Background Information and Scientific Rationale

## Background Information

### Background and Rationale of Malaria Challenge Studies

*Plasmodium falciparum* remains a leading cause of maternal and childhood mortality for populations in much of the tropical world, in particular sub-Saharan Africa, and a significant cause of morbidity in military personnel^1, 2^ and in travelers to these endemic areas.^3, 4^ Major impediments to the development of effective malaria vaccines include the lack of accurate *in vitro* correlates of protection, the paucity of laboratory-raised *P. falciparum* isolates against which to test candidate vaccines and a shortage of experienced vaccine testing centers and researchers. Our present understanding of the course of *P. falciparum* malaria in non-immune, experimentally infected humans has been derived from observations made during the course of using *P. falciparum* malaria as therapy for neurosyphilis and malaria drug prophylaxis trials. The goal of malaria infection in the treatment of neurosyphilis was to maintain a body temperature of > 40° C for as long as possible. This required that illness be maintained for as long as was tolerated, if possible until spontaneous resolution of the neurosyphilis. Peak parasitemias were greater than 100,000 parasites/mL^5^, and fatality rates of 4% were considered acceptable.^6^ Because persistent infection was a goal, spontaneous recrudescence was the rule. This is not the case with the present use of malaria challenge, whereby treatment fully eradicates the parasite and recrudescence has not been observed.^7, 8^

Drug efficacy studies from the 1940s through the 1970s provided further observations on the course of *P. falciparum* malaria in volunteers. In these studies, volunteers were allowed to remain parasitemic for several days prior to the initiation of effective therapy.^9-17^ Mean parasitemias were approximately 1000-fold greater than in these early historical malaria challenge studies, reaching 88,713 parasites/mL as compared to 46.9 parasites/mL in more recent studies.^18^

The clinical methods used to assess malaria vaccine efficacy in humans have been established through trials evaluating sporozoite-based, or *pre-erythrocytic stage*, malaria vaccines.^19-22^ During the *pre-patent period* (time before parasites are detectable in peripheral blood smears), volunteers are monitored closely for parasitemia and clinical sequelae. At *patency* (detectable parasites in peripheral blood smears), volunteers are treated promptly with a curative course of an oral anti-malarial drug. Immunized volunteers not developing parasitemia (that is not developing patent malarial infection) are considered protected, provided the non-immunized volunteers (controls), bitten at the same time and with the same group of infective-mosquitoes, experience a 100% attack rate.

The CVD has extensive human clinical trials experience with irradiated sporozoite immunization and malaria challenge studies from the late 1980s and 1990s.^7, 8, 23, 24^ The Walter Reed Army Institute of Research (WRAIR), Naval Medical Research Center (NMRC), Oxford University and Nijmegen University have continued to use the human malaria challenge model to evaluate antimalarial drugs and vaccines.^18, 25-27^ WRAIR has successfully used a similar malaria challenge model in over 400 volunteers, with > 300 patent malaria infections developing in the volunteers. They have experienced no deaths, episodes of severe malaria, anaphylactic emergencies, serious or unexpected complications or recrudescent malaria infections in any of these individuals (Kent Kester, MD, personal communication). The NMRC has a similarly robust experience with malaria challenge studies and an equivalent safety record.^8, 28^ One recent SAE, described as a coronary event, was recorded at the Radboud University Nijmegen Medical Center (RUNMC) and is described in detail in **Section** **2.3.1**. The etiology of this event remains unclear and work-up is ongoing. A second cardiovascular event occurred at this site in a 39 year old male and was felt to be coincidental with the volunteer’s participation in the trial.^29^ The collective experience of ~1400 challenge events has clearly established that the *in vivo* malaria challenge model can be safely employed to test the efficacy of drugs, subunit vaccines and attenuated sporozoite immunization.

No animal models of *P. falciparum* infection have convincingly predicted subunit vaccine efficacy in humans. *In vitro* immunological assays have not reproducibly correlated with vaccine efficacy in humans, with the possible exception of the development of vaccine peptide-specific CD4^+^ and CD8^+^ T cells producing interferon (IFN)-γ.^30, 31^ Furthermore, we do not know to what degree anti-sporozoite antibodies are functional *in vivo* or the protection conveyed, although opsonization by circumsporozoite protein-specific antibody might be one important protective mechanism.^32^ There were no clear correlations of immune responses in volunteers immunized with attenuated sporozoites who were protected or not from challenge^25, 33^, nor with the most effective pre-erythrocytic stage malaria vaccine tested to date.^34^

Sterile immunity to *Plasmodium spp.* has been achieved in mice, monkeys and humans after irradiated sporozoite injection.^23, 35-38^ Irradiated sporozoite development is arrested at the level of the hepatocyte, abrogating processing to the erythrocytic stage of parasite development. Evidence points to hepatic stage, MHC-restricted CD8^+^ T-cell-mediated responses as being crucial in mediating immunity to malaria, though CD4^+^ T-cells, cytokines and antibodies may act in concert to prevent disease.^39, 40^ Previously, the impracticalities of immunizing humans with irradiated sporozoites and the difficulties of including whole length malaria proteins in current vaccines prompted exploration of the use of epitope-based, pre-erythrocytic stage and other subunit malaria vaccines. HLA polymorphisms, allelic and antigenic diversity complicate this approach. The ability to aseptically raise mosquitoes and the advances in the processing and yield of sporozoite retrieval has made the use of irradiated sporozoite vaccines a viable vaccine option. Phase 2 sporozoite vaccine efficacy studies in humans are necessary to determine: 1) if the vaccine confers protection against heterologous strains, 2) which immunological assays, if any, correlate with protective immunity, and 3) if the candidate vaccine should be tested in endemic populations, a process that requires allocation of considerable financial and human resources.

The malaria challenge studies presented in this proposal are part of groundwork designed to create rigorous methodology to ensure critical evaluation of vaccine efficacy and antimalarial compounds in malaria-naïve volunteers. The model will ensure that each pre-challenge and challenge phase of work is able to proceed on schedule, and that challenge outcomes can be interpreted accurately and that volunteer safety is assured. The challenge model effectively predicted clinical efficacy for the RTS,S malaria vaccine prior to clinical field trials.^25^ As new candidate malaria vaccines reach clinical trial, the practice of these methods allows the protective efficacy of different vaccines and vaccine trials to be directly compared.

### Validation of Malaria Challenge Model Using Aseptic Mosquitoes

The goal of developing and validating the use of aseptic anopheles mosquitoes is to create a new standard for malaria challenge trials that will 1) reduce the variability of sporozoite load among mosquitoes both within and between mosquito lots, thereby reducing the variability of sporozoite loads introduced into human volunteers, and 2) decrease the already low risk to human volunteers of infection by microorganisms other than malaria, which may be carried by standard, laboratory-raised mosquitoes. Aseptic anopheles mosquitoes may contain greater sporozoite loads than traditional laboratory-raised mosquitoes, due to the fact that they have no co-infections. Co-infections may reduce the fitness of the mosquito and its ability to be parasitized by the malaria parasite. Elevated sporozoite loads may result in decreased numbers of mosquitoes needed to bite to achieve a 100% infective dose (ID_100_) which is the number of malaria-infected mosquitoes needed to ensure 100% of volunteers develop patent parasitemias. Co-infections, such as mycoplasma, fungal organisms and certain viral agents, could also place the human volunteer at risk of infection. This risk is currently theoretical, because such transmission has not been observed in the many hundreds of individuals challenged by the bites of *P. falciparum*-infected mosquitoes reared under “septic” conditions.

Persons are rarely bitten by more than one infected mosquito at a time in the natural setting. Malaria sporozoite challenge studies have traditionally relied upon the bites of five infected mosquitoes to induce malaria.^8, 19, 22, 41^ Fewer bites using mosquitoes raised in non-sterile conditions, do not reliably induce malaria in volunteers^42^, and increased bites may provide an unrealistic and overwhelming challenge in which even irradiated-sporozoite immunized volunteers fail to be protected from malaria challenge.^37^ While it is known that laboratory-reared anopheles, on average, have higher sporozoite loads in their salivary glands than do wild-caught anopheles mosquitoes^43^, it is not clear that the increased sporozoite loads in mosquitoes translates into greater sporozoite loads injected into volunteers.^44^ Sporozoite load is highly variable among both wild-caught^45^ and laboratory mosquitoes^46^, and may result in varying numbers of sporozoites being injected with each mosquito while biting.^44^ Greater numbers of sporozoites inoculated during malaria challenge studies has however, been suggested as the cause of prolonged parasitemia and poor antimalarial response in at least one trial.^47^ Furthermore, an increase in sporozoite and liver stage parasite burden may decrease the prepatent period.^15^ These factors are critically important during malaria challenge trials evaluating candidate malaria vaccine efficacy. A predictable consistency of the prepatent period must exist between challenge trials and among volunteers within a trial so that investigators are accurate in the interpretation of malaria events. Careful validation of the aseptic mosquito model with attention to prepatent periods, parasitemia and antimalarial response is critical in the validation of the aseptic challenge model. Based on historical data in 532 volunteers^18^, there is no reason to believe that factors such as age, race or gender affect infectivity in a malaria challenge so we would not expect these factors to play a role in the infectivity utilizing the aseptic challenge model. The use of standardized, aseptic mosquitoes with limited variability of sporozoite load could improve reproducibility of the prepatent period (currently noted to be between 7 and 21 days) and/or efficacy of the challenge event (estimated to be approximately 97% infective in the setting of a 5-bite challenge) between *P. falciparum* malaria challenge trials.

### Development and Validation of Molecular Diagnostic Methods

Polymerase chain reaction (PCR) techniques are beginning to be utilized in malaria challenge trials.^27, 48-50^ We will evaluate and optimize methods for early molecular diagnosis of *P. falciparum* malaria in the context of *P. falciparum* challenge trials. The primers reported in the literature are 100% homologous with the sequence from NF54, the isolate to be used in the trial proposed herein. The 18S (small subunit) ribosomal RNA gene is highly conserved^51^ and stage-specific^52^, thus likely to be a useful target across multiple clones. Real-time quantitative polymerase chain reaction (PCR) techniques can, at the very least, supplement direct microscopy as a diagnostic method in malaria challenge trials. Potential additional benefits include the ability to test blood stage (erythrocytic-stage) candidate vaccines, treat 100% of volunteers before they develop any symptoms of malaria and quantify the effect of a drug or vaccine on reducing liver stage development (by use of liver stage-specific real-time quantitative PCR (RTQ-PCR)). Refinement of such techniques could be extended even further to measure strain specific effects of vaccine and gametocyte specific effects of vaccines and drugs before endemic field trials. Field applications of these techniques could be possible albeit in a research setting.^53^

## Rationale

Challenge studies have been the preferred means to test the protective effect of vaccine constructs in Phase I studies in immunized, malaria-naive, U.S. volunteers. The ability to aseptically raise mosquitoes and advances in the processing and yield of sporozoite retrieval has made the use of irradiated sporozoite vaccines a viable vaccine option. The goal of this study is to evaluate the efficiency of aseptically raised Anopheles mosquitoes in transmitting *P. falciparum* infection to naïve; U.S. volunteers aged 18-40 years and establish a new standard operating procedure (SOP) and ID_100_ for challenge studies. Malaria challenge studies are useful to test the efficacy of pre-erythrocytic-stage malaria vaccines in malaria-naïve, volunteers. This first challenge study will be carried out as a “proof of concept” establishing the viability of aseptically-raised Anopheles mosquitoes as a vector in the challenge model and to bridge newer methodology such as PCR diagnosis to challenge study procedures for the NF54 chloroquine-sensitive *P. falciparum* isolate. The second goal of this protocol is to refine the number of mosquito bites required to induce 100% infectivity of falciparum malaria in this population. Refining the minimum number of mosquito bites required to induce 100% infectivity of falciparum malaria will also prevent the transmission of too many sporozoites that may have the effect of unnaturally “overwhelming” the immune response induced by the study vaccine and effectively eliminating the investigator’s ability to discern accurate protective efficacy. Once validity has been established in the NF54 strain of falciparum malaria (the subject of this protocol), which has historically been utilized in prior malaria challenge studies, long-range plans include studying additional Plasmodium strains developed from master cell lines and tested in challenge models. The diversity of falciparum malaria strains found worldwide argues for further development of alternate strain models for use in challenge studies. Ultimately, the goal will be to have multiple strains of falciparum parasite for challenge that are cryopreserved and packaged for transport to be used by needle and syringe inoculation by other research facilities and academic institutions testing malaria vaccines, thereby avoiding the need for a mosquito-bite challenge altogether.

## Potential Risks and Benefits

### Potential Risks

The risks of a malaria challenge study to U.S., malaria-naïve volunteers include the discomfort sustained by mosquito bites, the discomfort associated with periodic blood draws and the risk of acquiring clinical falciparum malaria. Mosquito bites are known to cause mild discomfort associated with mosquito feeding. A small amount of inflammation and pruritis typically accompanies the bite of the insect. While parenteral methods of parasite challenge have been attempted, newer modes of transmission have not been validated necessitating the traditional method of transmitting a parasite challenge via the bite of infected Anopheles mosquitoes. Anaphylaxis to the bite of a mosquito is extremely rare. A small amount of inflammation as a result of the mosquito bite is to be expected. The salivary components of the mosquito include anticoagulant, antiplatelet, vasodilatory, anti-inflammatory, and immunosuppressive proteins.^54^ Host tissue reaction can vary from small raised papules to large, pruritic wheals of inflammation and is a result of cutaneous mast cell response to salivary proteins released by the mosquito.^55^ The host response is due to both Type I (immediate, IgE-dependent) and Type IV (delayed hypersensitivity) reactions. The host response is variable depending on individual factors, sensitization and mosquito species. While significant allergic reactions are extremely rare, epinephrine, anti-histamines and ACLS equipment are available on site. Additional systemic signs that have been reported post-malaria challenge include mild malaise, headache and low-grade fevers. The symptoms resolved spontaneously. All clinical laboratory measurements remained within normal limits. These symptoms may relate to components within the salivary glands of mosquitoes that were injected via the bite of the insect and may or may not occur with the use of aseptically reared mosquitoes. A mild degree of discomfort may be sustained by the use of containment cages which are strapped onto the arm of a volunteer. No significant injury or discomfort has been reported utilizing this technique.

Two cardiovascular events have been described at a single site (RUNMC). In 2002, a 39 year old male was diagnosed with an inferoposterior myocardial infarction (MI) day 9 post-challenge.^29^ He was initially diagnosed with malaria via a blood smear on Day 8 but PCRs performed between days 5-9 (n = 10) were consistently negative and the blood smear was inferred to be a false positive. The event was felt to be coincidental with the volunteer’s participation in the trial. PCR analysis of the blood smear itself has been requested, but has not yet been done.

The second event (details confidential and unpublished), occurred in a 20 year old female who had received three doses at monthly intervals of an experimental malaria vaccine (recombinant LSA-3 formulated in alum) with severe headache noted after dose 3. She underwent NF54 strain sporozoite challenge on Day 140 post-vaccination dose 1 (~day 75 post-dose 3). The volunteer became parasitemic on Day 11 after challenge (44 parasites/µl) and was treated with artemether-lumefantrine (Riamet®; not currently FDA-approved for use in the U.S.), and was cleared by day 14 when treatment was terminated. On day 16 she awoke with sub-sternal, non-radiating chest pain and was medically evaluated with pain dissipating within 30 minutes coincident to sublingual nitroglycerin administration. Electrocardiographic tracings revealed < 1mm ST elevation inferiorly. Cardio Phophokinase levels were normal but the troponin level was elevated at 10.0 ng/mL. An echo revealed minimal inferior hypokinesis and an MRI was negative for cardiovascular disease or tissue damage. Subsequent evaluation revealed low hemoglobin on the day of challenge (6.9 mmol/L (normal range 8-12 mmol/L), and an abnormally elevated lumefantrine metabolite (desbutyl-lumefantrine) levels. Cholesterol, coagulopathy and autoantibody studies were normal or negative. No toxin screen was performed at the time of ER presentation but analysis of hair samples revealed no presence of illicit drugs. No reports of cardiovascular pathology or events have been reported with natural malaria and autopsy studies (n = 99) reveal sequestration of parasites in all organs but no evidence of myocardial damage. One episode of coronary spasm in a 17 year old male has been reported by Novartis after treatment of falciparum malaria with Coartem® (artemether-lumefantrine). A cannibis positive urine test was reported concomitantly. Extensive discussions of this second SAE at RUNMC have been conducted by the malaria challenge community worldwide and the collective opinion of this community (WRAIR, NMRC, Oxford, RUNMC) is that this case is not related to the malaria challenge event.

A review of safety and clinical outcome of 47 volunteers that underwent experimental challenge with NF54 strain at NMRC, revealed that one subject experienced pleuritic chest pain lasting 2 days (onset of symptoms occurred one day before onset of parasitemia), and another subject reported shortness of breath lasting 1 day (onset 2 days after parasitemia and initiation of chloroquine). These 2 events were reported as unsolicited symptoms, and no further clinical laboratory information was obtained.^18^

Periodic blood draws will be necessary to closely monitor the volunteers and to perform malaria smears and PCR analysis for early detection of *P. falciparum* parasitemia. Universal precautions will be maintained for the protection of the volunteer and the study personnel. Alcohol swabs will be used to minimize the potential of a cutaneous bacterial infection being introduced by needle sticks. Throughout this study, the amount of blood collected will be much less than 35 tablespoons (525 mL) in any 8-week period (which is the amount of blood allowed to be drawn under the American Association of Blood Banks standards).

The goal of this study to is determine the ID_100_ of the NF54 strain of *P. falciparum* transmission by the bites of a number of aseptically raised *A. stephensi* mosquitoes that is to be compared to the historical norm of 5 bites. Subclinical or mild malaria infections are expected. Vigilant analysis of the malaria smears (threshold of detection is approximately 10-50 parasites/mm^3^) will detect the presence of malaria infection in many participants before significant symptoms develop in the volunteer. PCR results (threshold of detection is approximately 10-20 parasites/mm^3^) will be compared to malaria smears retrospectively. Because therapy will be initiated with the detection of the first parasite by blood smear, dangerously high levels or prolonged duration of parasitemia will not occur. Mild malaria symptoms include headache, myalgia, fever, chills, sweats, nausea, vomiting, and diarrhea. Severe malaria evidenced by neurological manifestations such as coma and/or seizure and obtundation, profound anemia due to hemolysis and bone marrow suppression, acidosis, respiratory distress, acute renal failure, splenic rupture and vascular collapse with shock and death is highly unlikely and has never been described in an experimental malaria challenge. Manifestations of severe malaria are well described ^56^ and researchers at the University of Maryland have extensive expertise in the diagnosis and treatment of severe malaria.The pathogenesis of these conditions is multifactorial but a major factor is sequestration of parasite-infected red blood cells, which is proportional to the degree of parasitemia.^56^

Researchers at the CVD have extensive experience with malaria challenge as well as the care of clinical malaria and have shown conclusively that although volunteers often become symptomatic with mild malaria, rapid diagnosis and treatment quickly attenuates the illness so that the challenge is safe and does not place the volunteer at undue risk.^7^ The University of Maryland Medical Center is across the street from the SNBL facility and volunteers can be transported to this facility in the event that more intensive medical care is required. At the doses of drug used to treat experimental malaria, toxic manifestations of chloroquine are rare. Minor side effects include nausea, vomiting, dizziness, headache, diarrhea and pruritis. Atovaquone/proguanil (Malarone®) is a very well tolerated pharmaceutical agent that is relatively new to the market and is the second-line agent for chloroquine sensitive parasites. Malarone® will be used in the event of chloroquine intolerance or allergy. Mild side effects including nausea and diarrhea are noted with Malarone®.

**Table 1:** Number of volunteers undergoing sporozoite challenge

| **Institute** | **Timing** | **Strain** | **Volunteers (n)** | **SAE** |
| --- | --- | --- | --- | --- |
| **Army (WRAIR)** | Since 1985 | NF54 | 126 | None |
|  |  | 3D7 | 768 | None |
|  |  | 7G8 | 42 | None |
| **Navy (NMRC)** | Since 1998 | NF54 | 94 challenge, 72 infected | None |
|  |  | other | Unknown | None |
|  | Before 1998 |  | Unknown | None |
| **RUNMC**^a^ | Since 2001 | NF54 | 92 challenged, 87 infected | 2 |
| **Oxford** |  | 3D7 | 240 | None |
| **UMB-CVD**^a^ | Before 1991 | NF54 | 40 | None |

^a^ RUNMC refers to Radboud University Nijmegen Medical Center and UMMS-CVD refers to the University of Maryland at Baltimore-Center for Vaccine Development

**Table 2:** *P. falciparum* challenges in vaccinees and controls: CVD experience through 1991.

|  | Number of (%) Volunteers with | | |  |
| --- | --- | --- | --- | --- |
| Number of Volunteers^a^ | Fever Only^b^ | > 2 Symptoms^c^ | No Symptoms | Days to Patency (Range)^d^ |
| 40 | 18 (45) | 27 (68) | 4 (10) | 10.3 (7-14) |

^a^ Includes vaccinees and controls with the exception of the five vaccinees who were completely protected against parasitemia and illness. One vaccinee was challenged and protected twice. ^b^ Fever defined as temperature > 100.0 °F  ^c^ Symptoms include headache, backache, myalgias, chills, sweats, nausea, vomiting, diarrhea. ^d^ Maximum parasitemia = 1670 parasites/mL, 0.033% parasitemia

There is a risk to study personnel of being exposed to infectious mosquitoes during the manipulation of the infected *A. stephensi* and proper precautions will be taken to minimize exposure. Mosquitoes will be transported by Sanaria personnel in a specialized transport container designed to prevent mosquito escape. Additionally, each mosquito that is removed from the transport containers will be accounted for and tabulated under a biosafety laboratory hood. The challenge will occur at the CVD insectary which has a double door barrier system along with a double blower (negative pressure wind blockade) to prevent flight across entryways. In the event that a staff member is bitten inadvertently by a mosquito, prophylactic anti-malarial medication will be provided (Chloroquine or Atovaquone-proguanil (Malarone ®)).

### Known Potential Benefits

There are no known health benefits to be gained by the volunteer participating in this experiment. Free medical treatment related to the malaria challenge will be provided to enrolled participants during the active malaria challenge phase and the surveillance period. Medical care for ailments not related to challenge will not extend beyond the active study period (Day 56). Medical care for ailments related to challenge will extend, at minimum, until the condition has resolved or stabilized. No surgical treatments will be provided unless required for ailments related to the challenge. Trauma related injuries will be excluded from the free medical treatment provided under this study.

# Objectives

## Study Objectives

This study is designed to improve current methodology for malaria challenge studies to test the efficacy of malaria vaccine constructs and drugs after volunteer immunization. The use of aseptically-raised mosquitoes should reduce variability of sporozoite quantity between mosquito production lots, reduce variability in sporozoite transmission to human volunteers, and theoretically reduce the chance of co-infection that may be transmitted through a mosquito bite at the time of malaria challenge. The use of molecular techniques for parasite detection should enhance early detection of sub-clinical falciparum malaria infection and reduce the risk to human volunteers. These applications are critical to improving the methodology of malaria challenge studies and for standardization of the challenge model to all vaccine constructs.

### Overall study objective

Identification of an optimal, aseptically-raised *A. stephensi* mosquito bite challenge to safely transmit subclinical falciparum strain NF54 parasitemia to 100% (ID_100_) of malaria-naïve, U.S. volunteers for use in challenge models designed to test the efficacy of malaria vaccine constructs and drugs.

### Primary objective

1. Develop and evaluate the safety and tolerability of a new human malaria challenge model using aseptically-raised anopheles mosquitoes infected with the NF54 isolate of *P. falciparum* and reared under conditions similar to current Good Manufacturing Practice (cGMP)

### Secondary objectives

1. Obtain information on the minimum number of *A. stephensi* bites required to safely achieve 100% adult human volunteer infectivity (Malaria challenge, Part A).
2. Obtain information on the minimum quantity of *A. stephensi* bites in a second challenge study to achieve 100% adult human volunteer infectivity (Malaria challenge, Part B).
3. Develop molecular diagnostic techniques for rapid and accurate real-time diagnosis of *P. falciparum* infection, to assess a role as a new diagnostic standard for *P. falciparum* challenge studies.

## Study Outcome Measures

### Primary Outcome Measures

1. Occurrence of a positive malaria smear after the malaria challenge during a 56-day surveillance period (day of challenge and days 5-28, 35, 42, 49, and 56 after challenge)
2. Occurrence and severity of solicited symptoms for 7 days after the malaria challenge and for the duration of follow-up after a malaria event (treatment days x 3 and smear-negative x 2 plus weekly follow-up x 4).
3. Occurrence and severity of unsolicited adverse events after the malaria challenge during a 56-day surveillance period (day of challenge and 55 subsequent days. Volunteers will be questioned at Visits 2-29)
4. Occurrence of serious adverse events throughout the duration of the study.

### Secondary Outcome Measures

1. Occurrence of a positive real-time quantitative PCR after the malaria challenge during a 56-day surveillance period (day of challenge and days 5-28, 35, 42, 49, and 56 after challenge)

# Study Design

- An unmasked, dose-ranging, prospective Phase I cohort study (Malaria challenge, Part A), assessing whether the bite of one (if volunteer recruitment allows), three or five NF54-infected, aseptic anopheles mosquitoes results in an ID_100_ similar to that obtained with non-aseptic, insectary-raised mosquitoes. This is a “proof of concept” that will be used to compare to historical norms.
- A second challenge trial (Malaria challenge, Part B), dependent upon the results of the first will further discriminate whether a new lower bound of the ID_100_ is valid (i.e., if the bite of one, three or five anopheles mosquitoes successfully transmits NF54 parasites to all six volunteers, a second challenge study will explore the potential of a new lower bound for the ID_100_). This is a study designed to refine the dosage model as determined by the results of Part A. If the bite of five anopheles mosquitoes fails to successfully transmit parasites to all six volunteers, the study will not progress to Part B.
- Healthy, malaria-naïve, U.S. volunteers aged 18-40 years, inclusive
- Single center
- The test agent will be aseptic *Anopheles stephensi* mosquitoes infected with *P. falciparum* NF54 strain sporozoites by membrane feeding upon erythrocytes infected with *P. falciparum* gametocytes.
- The vector will be aseptically-raised, female, *A. stephensi* mosquitoes
- Malaria challenge, Part A will involve 18 volunteers randomized to six volunteers receiving 1 bite, six volunteers receiving 3 bites and six volunteers receiving 5 bites each. If less than 18 (12-17) volunteers are enrolled, the 1 bite cohort will be dropped in favor of continuing with randomization to the 3 and 5 bite cohorts.
- Malaria challenge, Part B will involve 20 volunteers challenged at the former threshold of 5 infected bites or the potential new lower bound (i.e., one or three infected mosquitoes) for the ID_100_
- Screening will be done within 60 days prior to the *Pf* NF54 malaria challenge
- Safety labs will be considered valid if obtained within 60 days of the *Pf* NF54 malaria challenge and will be repeated if the 60 day window elapses.
- Route of malaria challenge will be Anopheles bites to the forearm of volunteers allowing the mosquitoes to completely engorge
- Entomologists and research staff will be present to ensure that each mosquito completes only one bite. Mosquitoes will be removed for salivary gland dissection upon completing a blood meal (defined as ~5 minutes of feeding). Each bite is defined as a single blood meal accompanied by the presence of parasites within the salivary glands of the individual Anopheles mosquitoes. A blood meal by a mosquito that lacks *P. falciparum* parasites within the salivary gland will not be quantified as a bite and a replacement mosquito will be added to the challenge (until randomized bite quantity administered). Cohorts will be quantified as 5 or 3 or 1 bite based on the completion of the number of defined bites.
- Study duration will be approximately 2 months (56 days) of active follow-up per participant followed by 10 months of passive surveillance for a total of one year of follow-up.
- 56-day surveillance (day of challenge and days 5-28, 35, 42, 49, and 56 after challenge) for the development of a positive malaria smear
- “Real-time” PCR results will be compared retrospectively to the current “gold standard” malaria blood smear 56-day surveillance data to determine if superior detection capability exists with this technology. This will be a sub-study (See **Section 4.1**) for use in validating PCR as a diagnostic test in future challenge studies and will not be used as the primary mode of diagnosing blood-stage malaria infection during this study.
- Chloroquine will be used to treat all blood-stage malaria infections with clinical and laboratory surveillance weekly for 4 weeks after completion of antimalarial therapy. Malarone® will serve as second line therapy in the event of intolerance or allergy to chloroquine.
- Malarone® will be offered as first line therapy in the event of volunteer withdrawal from the study prior to onset of a clinical malaria episode (Refer to **Section 7.5**, Early Termination). This volunteer will be strongly urged to consent to malaria therapy for safety reasons.
- Follow-up of serious adverse events (SAEs) until resolution
- Laboratory analysis (CBC (WBC, hemoglobin and platelets) and biochemistries (creatinine, glucose, AST and ALT)) will be performed on the day of challenge, each day of a positive blood smear, for the duration of antimalarial treatment and at each weekly follow-up thereafter.
- Analysis of cardiovascular risk and safety evaluation will be performed at screening and at days of follow-up. Cardiovascular-related symptoms (chest pain or discomfort, shortness of breath, change in exercise tolerance, and palpitations) will be solicited. Subjects with abnormal cardiovascular symptoms or findings will be referred to a cardiologist for further evaluation.

1. Screening: Cardiovascular risk will be screened for in all volunteers. A 12-lead ECG will be performed along with the banking of sera for troponin levels which will be frozen at -80° C and run as a baseline in the event that a follow-up troponin level in an asymptomatic individual is positive.
2. Follow-up: A 12-lead ECG and sera troponin level will be performed on day three post-malaria patency and the one week follow-up post-inpatient discharge (e.g. if malaria patency occurs on day 10 post-challenge, day 12 post-challenge would represent the third day post-malaria patency. The one week follow-up will occur 7 days (+/- 2 days) after inpatient discharge (~ day 20)).

- The primary analysis will be conducted for all primary and secondary endpoints at a data-lock-point one-month post malaria challenge A study conclusion (study day 90), after which the Primary Study Report will be produced.
- The study will continue to Malaria Challenge, Part B based on the results of malaria challenge, Part A (Refer to Primary Objective 1).
- Safety oversight per DMID guidelines will be provided by an independent Safety Monitoring Committee (SMC) (Refer to <http://www.niaid.nih.gov/dmid/clinresearch/>). The committee will be composed of individuals who are not directly involved in the study along with a local independent safety monitor (ISM) from the University of Maryland Medical System who will provide local medical consultation for the study. The committee will meet to review cumulative safety data, adherence to the protocol and data relevant to proceeding to the Part B of the study. (Refer to **Section 9.5.2**)

## Substudies (Real time PCR analysis)

Real time polymerase chain reaction (RTQ-PCR) offers the advantage of providing an alternate mechanism of diagnosis to the gold standard method of examining blood smears. The utility of the RTQ-PCR has been the sensitivity of the assay but this can also be a detriment in that false-positive results have been reported. In concert with the ongoing challenge study and diagnostic malaria blood smears, we intend to evaluate the utility of the RTQ-PCR technique with the results of the malaria blood smear. This will be used as a complementary test but will not be used for diagnosis.

- A quantity of blood will be drawn concurrently (once daily) with the malaria blood smear for the purposes of conducting RTQ-PCR analysis.
- RTQ-PCR samples will be batched and run on the following day after collection utilizing the Applied Biosystems 7300 RT PCR apparatus. Samples drawn on the weekends will be performed on the following Monday. Samples drawn on holidays will be run on the next business day. Batched samples that cannot be completed within the next business day (due to excess sample quantity or machinery failure) will be performed as soon as reasonably possible to adhere to real-time simulation.
- The results of the RTQ-PCR will not be utilized in the diagnosis/confirmation of malaria for clinical volunteers but will be compared retrospectively to the results of the blood smear.
- RTQ-PCR results will remain blinded to clinical personnel until after the study at which time the sensitivity, specificity, negative and positive predictive values will be generated in an unbiased fashion.
- RTQ-PCR results will be compared to the intensity of signs and symptoms of malaria events.

# Study Enrollment and Withdrawal

The study population will be enrolled from a CVD database of volunteers from the greater Baltimore-Washington metropolitan area and will include ~38 U.S. malaria-naïve, volunteers (18 volunteers for malaria challenge, Part A and 20 volunteers for malaria challenge, Part B) aged 18-40 years. Enrollment will occur over a 1-2 month period and the target population will reflect the community at large at the study site. Recognizing the inherent difficulties of recruitment for this study, a contingency plan has been designed. If aggressive recruitment fails to enroll 18 volunteers, the 1 bite cohort arm will be dropped. The study will only proceed if 12-17 volunteers have been enrolled and can be randomized to either the 3 or 5 bite cohorts. Remaining volunteers will be encouraged to participate in Part B. The 1 bite cohort will establish a lower bound for the aseptic challenge model but we do not plan on proceeding with a 1 bite challenge model in future malaria challenge designs due to unreliability of a single bite as an dependable means of challenge. Information regarding study materials will be mailed to potential subjects who have previously participated in vaccine trials or expressed willingness to participate at the enrollment sites and have signed an authorization form that they are willing to be contacted for future studies. This has been done with Institutional Review Board (IRB) approval. Educational meetings will be offered to the public both within and outside the university system for the purposes of informing individuals of the study. Every effort will be made to achieve a balanced volunteer population while avoiding the enrollment of vulnerable populations. Careful screening will be conducted to avoid enrollment of individuals with substance abuse or psychiatric difficulties.

Due to the occurrence of a cardiovascular event (described in **Section 2.3.1**), and at the request of DMID, screening for cardiovascular disease risks and sub-clinical cardiovascular disease will be performed. Post-challenge 12-lead ECG and troponin levels will be performed regardless of symptomatology on day three post-malaria patency and the one week follow-up post-inpatient discharge. Volunteers will be closely examined and questioned at all follow-ups for presence of cardiovascular-related signs or symptoms. Of note, following the two cardiovascular SAEs at RUNBC, local IRBs have approved challenge studies at several experienced centers throughout the world including WRAIR (no protocol alterations), NMRC (reference to SAE included in informed consent), and Oxford, U.K., (12-ECG and cardiovascular disease risks screened in volunteers prior to enrollment).

Of note, FDA/CBER informed the NMRC on 13 November 2008 that cardiac screening, a baseline EKG and monitoring would be needed prior to initiation of a scheduled malaria challenge on 17 November 2008. Expedited IRB approval then needed to be obtained in order for the challenge trial to proceed as scheduled (personal communication, Tom Richie and Chris Ockenhouse). Malaria challenge commenced on schedule.

The Center for Vaccine Development (CVD) will be the site for volunteer enrollment and malaria challenge events. Malaria challenge events will only occur in the protected insectary facility at the Center for Vaccine Development. The inpatient evaluation and outpatient follow-up will be performed at both the SNBL (or the back-up facility, GCRC) and the CVD outpatient facilities.

## Subject Inclusion Criteria

Subjects meeting all of the following inclusion criteria will be eligible to participate in this study: Refer to **Section 9.2.4** for reference ranges for normal laboratory values.

1. Male or nonpregnant female between the ages of 18 and 40 years, inclusive.
2. Women who are not surgically sterile (no history of bilateral tubal ligation, bilateral salpingo-oophorectomy, or hysterectomy), post-menopausal (1 year without menses) or determined otherwise by medical evaluation to be sterile must agree to practice adequate contraception (such as double barrier methods (condoms plus foam or spermicide, diaphragm plus foam or spermicide), some intrauterine devices (IUDs), intravaginal or transdermal hormonal methods initiated at least 1 month prior to inoculation, or a vasectomized partner) for the entire study period (56 days). Serologic pregnancy tests will be conducted upon screening. Urine testing will be done on the day of malaria challenge, on the day of the first positive malaria smear and at the conclusion of active surveillance (Day 56).
3. Is in good health, as determined by vital signs (heart rate, blood pressure, oral temperature), medical history, screening 12-lead ECG and a physical examination.
4. Has normal laboratory values (Urinalysis (assessing blood, and protein presence as greater than trace by dipstick), hemoglobin, WBC, platelet count, AST, ALT, glucose, and creatinine) prior to challenge study.
5. Able to understand and comply with planned study procedures including an inpatient stay from Day 8-18 after malaria challenge.
6. Provides informed consent prior to any study procedures, correctly answers > 70% on the post consent quiz and is available for all study visits.
7. Willing to avoid non-study related blood donation for 3 years^57^ following *P. falciparum* challenge

## Subject Exclusion Criteria

Subjects meeting any of the following exclusion criteria will be excluded from study participation:

1. Has any known history of malaria infection, is a long-term resident (> 5 years) of a malaria-endemic area, was born and resided in a malaria-endemic area, or has traveled to a malaria-endemic area within the previous 6 months.
2. Has a positive urine pregnancy test prior to malaria challenge (if female of childbearing potential), is lactating, or has the intention to become pregnant within 2 months after enrollment in this study.
3. Use of any antibiotic or antimalarial drug beginning 28 days prior to the screening and extending to Day 56 of study surveillance.
4. Has evidence of increased cardiovascular disease risk (defined as > 10%, 5 year risk) as determined by the method of Gaziano.^58^ Risk factors include sex, age (years), systolic blood pressure (mm Hg), smoking staus (current vs. past or never), BMI (kg/mm^2^), reported diabetes status (yes/no), current treatment for raised blood pressure (yes/no).
5. Is immunosuppressed (e.g., poorly-controlled diabetes mellitus, cirrhosis, renal insufficiency, active malignancy, connective tissue disease, organ transplant) as a result of an underlying illness or treatment.
6. An abnormal EKG, defined as one showing pathologic Q waves and significant ST-T wave changes; left ventricular hypertrophy; any non-sinus rhythm excluding isolated premature atrial contractions; right or left bundle branch block; or advanced (secondary or tertiary) A-V heart block.
7. Has an active neoplastic disease (excluding nonmelanotic skin cancer) or neoplastic disease within the past 5 years or any history of hematologic malignancy.
8. Is using or intends to continue using oral or parenteral steroids, high-dose inhaled steroids (>800 μg/day of beclomethasone dipropionate or equivalent) or other immunosuppressive or cytotoxic drugs (an exception includes asthma for which any oral or inhaled steroid intake will mean exclusion from study enrollment).
9. Has a known active history of human immunodeficiency virus, hepatitis B surface antigen positivity, or Hepatitis C infection.
10. Has a history of active alcohol or drug abuse in the last 5 years.
11. Has a history of receiving blood products within the 3 months prior to enrollment in this study.
12. Has a history of psoriasis or porphyria, which may be exacerbated after treatment with chloroquine.
13. Has an acute or chronic medical condition that, in the opinion of the investigator, would render malaria challenge unsafe or would interfere with the evaluation of responses (this includes, but is not limited to: known liver disease, renal disease, neurological disorders, visual field defects, cardiac disorders, pulmonary disorders, auditory damage, diabetes mellitus, and transplant recipients).
14. Has a history of anaphylactic response to mosquito bites or known allergy to chloroquine, 4-aminoquinoline derivatives (atovaquone/proguanil (Malarone®)), ibuprofen, or acetaminophen that may be used to treat volunteers developing malaria after *P. falciparum* challenge.
15. Is using or intends to continuing using a medication known to cause drug reactions with chloroquine or Malarone®, such as cimetidine, metoclopramide, antacids or kaolin (antacids and kaolin can be administered at least 4 hours from intake of chloroquine).
16. History of retinal or visual field changes, auditory damage, or seizures.
17. History of splenectomy
18. Has known sickle cell trait or laboratory evidence of sickle cell trait.
19. Has an acute illness, including an oral temperature greater than 100.4°F, within 1 week prior to malaria challenge.
20. Plans to undergo surgery (elective or otherwise) between enrollment and 4 weeks (28 days) post-challenge
21. Received an experimental agent (vaccine, drug, biologic, device, blood product, or medication) within 1 month prior to enrollment in this study, or expects to receive an experimental agent during the 2-month post-challenge period.
22. Has a diagnosis of schizophrenia, bi-polar disease or other major psychiatric disease.
23. Has any condition that would, in the opinion of the site investigator, place the subject at an unacceptable risk of injury or render the subject unable to meet the requirements of the protocol.

## Treatment Assignment Procedures

### Randomization Procedures

Eighteen subjects will be randomly assigned to 1 of 3 challenge groups in a 1:1:1 ratio. Participants will be randomized to one of three groups of 6 subjects (1, 3 and 5 bites). Volunteers who withdraw prior to the malaria challenge will be replaced. Screening records will be kept on subjects who have signed the consent form. If the ID_100_ (100% infectivity for all cohort volunteers) is not achieved, the study will not progress to Part B.

**Table 3:**

| **Anopheles challenge, Part A** | **Number of Subjects** |
| --- | --- |
| 5 bites | 6 subjects |
| 3 bites | 6 subjects |
| 1 bite* | 6 subjects |
| **Anopheles challenge, Part B** |  |
| Determined by challenge A** | 20 subjects |

*

* The one bite cohort may be dropped if less than 18 (12-17) volunteers are enrolled. Randomization to the 3 and 5 bite cohorts will proceed.

** The number of Anopheles bites used in subsequent challenge will be determined by the cohort with the fewest number of bites required to obtain an ID_100_.

Randomization will be done online using the enrollment module of AdvantageEDC and the randomization tables provided by biostatisticians at EMMES. The randomization code will provide assignment to the challenge cohort. Study numbers will be assigned to participants of each cohort in the order in which they provide written informed consent to be enrolled in the trial.

**Table 4:** Timeline for safety reviews of malaria challenge studies, Part A and B. Active study participation is denoted by “x”. The Safety monitoring committee (SMC) and Independent Safety Monitor (ISM) will convene on Day 60 (Day 56-91) of Part A and B to review safety data. A recommendation as to whether the study can proceed will be made within 7 days for Part B. The challenge dose for Part B will be informed by the results of Part A (See **Section 11.4.2**).

|  | **Malaria Challenge Part A** | | | | | | | | **Part B^d^** | | | | | | | | | | | | |
| --- | --- | --- | --- | --- | --- | --- | --- | --- | --- | --- | --- | --- | --- | --- | --- | --- | --- | --- | --- | --- | --- |
| Week | **1** | **2** | **3** | **4** | **5** | **6** | **7** | **8** | | **9** | **10** | **11** | **12** | **13** | **14** | **15** | **16** | **17** | **18** | **19** | **(24)** |
| Day |  |  |  | **30** |  |  |  | **60** | |  |  |  | **90** |  |  |  | **120** |  |  |  | **180** |
| 5 bites | x | x | x | x | x | x | x | x | |  |  |  |  |  |  |  |  |  |  |  |  |
| 3 bites | x | x | x | x | x | x | x | x | |  |  |  |  |  |  |  |  |  |  |  |  |
| 1 bite^a^ | x | x | x | x | x | x | x | x | |  |  |  |  |  |  |  |  |  |  |  |  |
| **Optimal Bite^b^** |  |  |  |  |  |  |  |  | |  |  |  | x | x | x | x | x | x | x | x |  |
| ISM ^c^ |  |  |  |  |  |  |  | x | |  |  |  | x |  |  |  |  |  |  |  | x |
| SMC |  |  |  |  |  |  |  | x | |  |  |  | x |  |  |  |  |  |  |  | x |

^a^ The 1 bite cohort will proceed if enrollment numbers allow.

^b^Optimal bite refers to the minimum number of *A. stephensi* bites required to safely achieve 100% adult human volunteer infectivity

^c^ The ISM and SMC will meet on ~Day 60 (Days 56-91) after Part A to review 56 safety data prior to progressing to Part B and ~Day 60 (56-91) after Part B to review 56-day safety data.

^d^ Malaria Challenge Part A and B inpatient follow-up will occur at the SNBL Biotechnology Park. The study will resume at least 7 days after safety review of Part A. The General Clinical Research Center may serve as back-up.

### Masking Procedures

Following randomized assignment of study participants to groups and doses, an unblinded study team member will administer the appropriate challenge. Volunteers will be assigned an anonymized study number. The nature of the challenge is such that the number of Anopheles mosquitoes used for challenge will be clearly visible to both the study participant and the administrator, and inflammation from the bites will remain upon the forearm of the study participant for quantification. The study personnel who do not participate in the challenge and who perform any study assessments (i.e., reading of malaria smears, etc.) will be blinded to the study cohort for Part A of the challenge study. Results will be unblinded prior to Part B in order to determine the optimal number of bites determined to confer 100% infectivity. Part B will be comprised of study subjects receiving this optimized number of bites and therefore, will be known to study investigators. The SMC will receive data in aggregate and sorted by challenge cohort. They will meet to review 56 day safety data for each challenge (Table 4) within five weeks of Cohort completion of Day 56 (Days 56-91). Refer to **Section 9.5.2** for full SMC guidelines. The SMC will receive 56 day safety data (including AEs, laboratory data through Day 56 and malaria smear results through week 4 of outpatient weekly surveillance visits (Refer to Tables 5 and 6)) for each challenge.

### Reasons for Withdrawal/Discontinuation

The following criteria will be checked at each visit. If any become applicable during the study, the volunteer’s continued participation in the study will be evaluated on a case by case basis. If it is felt that inclusion of the study volunteer’s data for analysis is compromised, the study volunteer will be terminated from the study and study data will not be included in analysis. This does not preclude the ethical responsibility of the investigators to ensure the safety of the volunteer and offer curative therapy for a malaria episode and to follow the volunteer for cardiac manifestations of disease. If the volunteer has already experienced a malaria episode and received curative therapy, continued inclusion in the study will be considered. All data generated before withdrawal will be included in final study analysis.

- Use of any antibiotic or antimalarial drug not approved by the study personnel beginning 28 days prior to the malaria challenge and extending to day 56 of study surveillance.
- Use of any investigational drug or vaccine post-malaria challenge during the study surveillance (Day of challenge and 55 subsequent days).
- Evidence of cardiovascular-related symptoms to include chest pain or discomfort, shortness of breath, change in exercise tolerance, and palpitations.
- Chronic administration (defined as more than 14 days) of any dose level of immunosuppressants or other immune-modifying drugs during the study period and chronic daily use of inhaled steroids. Intermittent use of inhaled (<800 μg/day of beclomethasone dipropionate or equivalent) and topical steroids are allowed.
- Administration of any blood products up to Day 56 of study surveillance.
- Systemic hypersensitivity reaction following administration of the Anopheles challenge. Severe (i.e., Grade III) local reactions will be evaluated to determine whether or not further study participation is warranted and whether local or systemic therapy is necessary
- Per the discretion of the PI in the event that circumstances arise preventing the subject’s continuation in the study, (i.e. pregnancy, some SAEs, noncompliance).

Subjects are free to withdraw from participating in the study at any time upon their request.

A study subject will be discontinued from active participation in the study (and followed for safety) if:

- Any clinical adverse event (AE), laboratory abnormality, intercurrent illness, or other medical condition or situation occurs such that continued participation in the study would not be in the best interest of the subject.
- Development of any exclusion criteria may be cause for discontinuation.

Subjects are free to withdraw from participating in the study at any time upon request. Participants who elect to withdraw from the study will not be subjected to additional research portions of the study (research blood draws, RTQ-PCR, etc.) although they will be strongly advised to continue with the safety portions (i.e., blood smears, 12-lead ECG, troponin levels) of the study.

### Handling of Withdrawals

Every effort will be made to collect safety and laboratory data on any subject discontinued because of an AE or SAE by continuing the safety follow-up procedures. If voluntary withdrawal occurs, the subject will be asked to continue scheduled evaluations and be given appropriate care under medical supervision until the symptoms of any malaria episode or AE resolve or the subject’s condition becomes stable. If possible, subjects who leave the study area will be traced and visited by clinical investigators to collect safety follow-up data. Volunteers will be asked to wear identifying safety bracelets or necklaces, stating that they have been exposed to infective malaria parasites and listing contact information for study personnel, and will be contacted by cell phone (or beeper provided if they do not possess this item). Subjects will not be replaced in the event that they choose to withdraw from the study after the malaria challenge. The study design does allow for a 20% loss to follow-up (2 volunteers). Study non-compliance will be defined as missing 2 sequential outpatient evaluations or more than 24 hours (2 consecutive malaria smears) of contiguous in-patient evaluation without notification or explanation to allow for verbal follow-up. All data generated before withdrawal will be included in final study analysis, and volunteers will be followed for safety. Events that occur and are not subject to participant control (e.g., death in the family) will be addressed on a case by case basis.

### Termination of Study

This trial may be terminated by the PI, sponsor or funding agency due to the development of a serious malaria event resulting in laboratory toxicity or at the recommendation of the SMC if serious safety concerns are identified.

# Study Intervention/Investigational Product

## Study Product Description

### Acquisition

Sanaria, Inc. will provide the aseptic mosquitoes and parasites utilized in this protocol. The production method will be the subject of a Biologics Master File (BMF). Sanaria will deliver the mosquitoes for the challenge study to the University of Maryland, Center for Vaccine Development.

### Formulation, Packaging, and Labeling

Aseptically-raised *Anopheles stephensi* mosquitoes to be utilized in the challenge study are infected with *P. falciparum* parasites of the NF54 strain utilizing methods developed by. Sanaria, Inc. Briefly, eggs from *A. stephensi* mosquitoes are chemically sterilized and placed in media for nutrition and growth. After sterilization, a proportion of these eggs are cultured to assess bioburden. The eggs pass through a larval and pupae stage before emerging as adults. Adult female Anopheles mosquitoes are given a blood meal utilizing erythrocytes infected with *Pf* NF54. A proportion of the mosquitoes are cultured at each developmental stage to assess bioburden. If at any point during the growth phase, there is any detectable microbial growth in the bioburden testing, then the products of those containers are destroyed.

On the day prior to challenge, containers of aseptic mosquitoes that have passed the bioburden criteria, are sampled for the percentage of mosquitoes with *P. falciparum* sporozoites in their salivary glands, and the mean numbers of sporozoites per infected mosquito. Only containers with a mean sporozoite count greater than or equal to 10,000 sporozoites per infected mosquito and an estimated final female mosquito survival rate of ≥ 40% will be used.

The containers chosen for the experimental challenge will be transported to the University of Maryland to the insectary for use in the malaria challenge studies. Safety will be maintained. A full description of the procedures performed by Sanaria in transport are included in the Biologics Master File to be submitted to the FDA by Sanaria. The collective experience spanning decades of both the CVD and Sanaria personnel ensures for safe transport. University of Maryland personnel have successfully transported infected mosquitoes to the CVD from NMRC in Rockville, MD for malaria challenge studies ^24^ and from USAMRIID in Frederick, MD for dengue biting studies.^59^ Most recently (November 2007), mosquitoes infected with *P. falciparum* sporozoites were safely transported to Oxford, England for a challenge study.

### Product Storage and Stability

The test product for this study will be aseptically-raised *A. stephensi* female mosquitoes infected with aseptically-raised *P. falciparum* parasites of the NF54 strain. These mosquitoes require carefully regulated environmental conditions such as temperature and humidity. Upon transfer to the University of Maryland, Center for Vaccine Development (a distance of approximately 35 miles from Sanaria, Inc. in Rockville, MD), the containers of mosquitoes will be placed in the insectary utilizing incubators that will maintain appropriate environmental conditions. The security of the insectary is critical and, as such, every effort will be made to restrict access to the insectary and provide for a secure location.

The University of Maryland has a protected suite of 920 sq ft. devoted to an insectary, containment area, laboratory, and microscopy rooms, as well as incubation facilities for proper maintenance of temperature and humidity. The current facility was established in 1995 and renovated in 2008. A walk-in environmental chamber serves as the insectary. The temperature can be rigorously maintained (generally at 28°C with humidity maintained between 70-80%). A hygrothermograph record of the insectary environment can be maintained and checked daily. Inside the insectary, photoperiods are regulated by sunrise/sunset simulators. A two-door barrier system along with negative pressure blowers (Dyneform Model ICL-48 (Windsor, CT)) prevent the migration of infected mosquitoes from the high containment challenge room to the waiting antechamber. An additional barrier door prevents mosquito migration into the clean molecular laboratory space. The antechamber outside the laboratory space and challenge area provides a waiting area for challenge volunteers. An additional negative pressure blower and barrier door is located between the waiting area and the outside CVD facility corridor. Thus, a total of two blowers and three barrier doors exist between the challenge facility and the outside corridor. Access to the facility will be restricted to study personnel only. Volunteers will only be permitted to enter the waiting chamber under the supervision of study personnel and will be escorted out of the chamber with study personnel. Volunteers will be accompanied to the challenge chamber in groups of 2-4 individuals. After challenge, all volunteers will be instructed to practice mosquito risk reduction methods in order to prevent the remote possibility of transmission of *P. falciparum* to local *Anopheles* mosquitoes.

## Dosage, Preparation and Administration of Study Intervention/Investigational Product

Malaria challenge is conducted by strapping an inoculation containment cage (a cardboard pint container) of *P. falciparum*-infected mosquitoes to the forearm of volunteers and allowing the mosquitoes to completely engorge.^8, 23, 24, 60^ Containers with mosquitoes meeting pre-defined criteria will be transported from the Sanaria facilities in Rockville, MD (refer to Investigator’s Brochure for full detail). The criteria for inclusion of mosquitoes used in the proposed human studies include a mean of 10,000 sporozoites per paired salivary gland, > 40% female survival within the container and > 40% of sampled mosquitoes infected with sporozoites. The mean of 10,000 sporozoites per paired salivary gland is a number that is may be greater than that used as a cut-off criteria in previous malaria trials. In the past, the mosquito gland score has been the method of estimating the amount of sporozoites present in paired salivary glands of the mosquito upon dissection. The gland scores have been as follows: 0 (no sporozoites), 1 (1-10), 2 (11-100), 3 (101-1000), 4 (> 1000).^44^ In virtually all previous studies, mosquitoes with a gland score of > 2+ (> 11-100 sporozoites per paired gland) were considered infectious.^8^ It is thought that regardless of the density of sporozoites in the salivary glands, only a limited number of sporozoites can be inoculated during a 5-10 minute period (approximately 5-300),^61^ because of the physical characteristics of the mosquito salivary duct they must traverse. Thus, from a theoretical perspective, it has not been expected that there will be many more sporozoites inoculated, regardless of the numbers of sporozoites per mosquito. Colleagues at RUNMC have utilized mosquitoes with 10,000 sporozoites per paired salivary gland as a cutoff and have found a slighted reduced pre-patent period (9 days (range 8-13)^29^ as compared to a mean of 10.5 days (range 9-14) reported by Epstein et al.^18^ Nevertheless, the highest mean parasitemia in Nijmegen (55 parasites per ul blood) is equivalent to an approximately 0.001% parasitemia. If the volunteers have a decreased pre-patent period, they will be diagnosed and treated earlier, but they will not develop higher levels of parasitemia, and will not be at any increased risk of severe disease.

Prior to human challenge, the quantity of mosquitoes required for the randomized volunteer in the cohort to which they have been enrolled (5 vs. 3 vs. 1* mosquito) will be removed from the container and placed in a pint, cylindrical cardboard containers with a nylon screen on the top of the container. * The 1 bite cohort will proceed if study enrollment numbers allow. After the starved mosquitoes are allowed to engage in a blood meal and engorge (typically for ~5 minutes), the blooded mosquitoes that participated in feeding will be removed and dissected by an entomologist to determine 1) whether a blood meal was taken and 2) whether the mosquito had *Pf* sporozoites in her salivary glands. The total challenge event is anticipated to last 10-20 minutes depending upon the randomized bite group. If necessary, additional mosquitoes will be obtained from the incubating containers in the insectary for use in additional feeds until the requisite numbers of infected mosquitoes with adequate sporozoite quantities have fed upon the participant within that cohort. This may extend the time of challenge past the predicted 10-20 minute time frame. All mosquitoes remaining after completion of the study will be destroyed according to appropriate SOPs. Neither the volunteers, study personnel participating in the challenge events, nor entomology team will be blinded to the cohort.

## Modification of Study Intervention/Investigational Product for a Participant

It is important that each participant receive the exact number of infected mosquito bites as dictated by the randomized cohort. This requirement is critical to allow refinement of the model and adjust the number of infected mosquito bites necessary to induce an ID_100_. As such, no modification of dose will be allowed in Malaria Challenge, Part A. Malaria Challenge, Part B is dependent upon the results of Part A as we seek to determine the number of mosquito bites necessary and sufficient for reliable transmission of falciparum malaria. We will be able to ensure that each mosquito has 10,000 or greater sporozoites present in the paired salivary gland, by dissecting each challenge mosquito after feeding on the volunteer. Studies at Sanaria have demonstrated that very high sporozoite burdens are achieved in these aseptically-raised mosquitoes and that these levels can be induced routinely. Whether this high sporozoite burden translates to more efficient transmission of the parasite remains a matter of debate^42^ and will be part of what we seek to determine through the conduct of this trial. It is unclear if the number of sporozoite present in the glands results in increased numbers of sporozoites injected. In laboratory studies, the number of sporozoites injected per bite can vary by as much as 3 orders of magnitude.^62^ ***Reducing the number of mosquito bites to that absolutely required to attain an ID_100_ is necessary to avoid the problem of unnaturally introducing too many sporozoites at a malaria challenge. This could result in overwhelming the vaccine-induced protection elicited against the parasite which might ordinarily be sufficient under natural conditions of exposure.***

## Accountability Procedures for the Study Intervention/Investigational Product

The infected mosquitoes will delivered to the UMD CVD by Sanaria in a container designed to transport the mosquitoes in an aseptic fashion. Only the investigators and laboratory technicians along with the entomology team will have access to the insectary. The insectary is maintained in a locked facility with guards stationed at the entry portal to ensure that only proper personnel are allowed into the Center for Vaccine Development. After bite administration, the mosquitoes used in the challenge will be dissected and results recorded into a dissection log to be maintained by the CVD. The remaining mosquitoes that were not used in the study will be destroyed. Typically, mosquitoes are destroyed by freezing at -20 degrees centigrade for 24 hours followed by appropriate disposal into Biohazard waste.

## Assessment of Subject Compliance with Study Intervention/Investigational Product

Not applicable

## Concomitant Medications/Treatments

At each study visit/contact, the investigator will question the participant about any medication taken, including holistic/naturopathic medications. Concomitant medication, including vaccines, and any other medication relevant to the protocol, including any specifically contraindicated (e.g., antibiotics or antimalarials within 28 days, oral or parenteral steroids, high-dose steroids, cimetidine, metoclopramide, antacids, kaolin, or immunosuppressive or cytotoxic drugs, experimental vaccine or agent) or administered during the period starting from one week before (with the exception of antibiotics and antimalarials) each malaria challenge and ending two months (maximum 56 days) after will be recorded in the CRF with trade name and/or generic name of the medication, medical indication, start and end dates of treatment. If a volunteer has initiated an antacid or kaolin, any administration of chloroquine will be timed to administer antimalarial medication by more than 4 hours to prevent absorption interference.

# Study Schedule

## Screening

**Days -60 to -1:** Recruitment will be progressive and staged in two phases (Part A and Part B) until 18 and 20 individuals of either gender who fulfill the inclusion criteria are included. Volunteers will be recruited among adults 18-40 years of age who have been previously enrolled in CVD studies and reside in the greater Baltimore/Washington metropolitan area. Additionally, educational seminars will be offered from which interested parties may request information on participation if desired. After the study has been explained to the potential volunteers, they may leave and return later with their decision allowing time for careful consideration of their involvement in the study. The informed consent process will be conducted in private room at the Center for Vaccine Development (CVD) to ensure confidentiality.

After community information is disseminated as described in **Section 14.3**, all interested individuals will be invited to visit the study clinic on a specific date. These individuals will receive oral and written explanation of the study, after which consent will be obtained from those willing to participate. All screening tests, medical history and examinations will be performed only after an informed consent is obtained. A volunteer quiz consisting of 10 questions will be administered. A passing score of 70% will be required prior to enrollment.

Upon screening, the Investigator will prepare a screening form for each participant. This screening form will later become part of the CRF for participants enrolled in the challenge trial. A unique identification number will be assigned to each study participant. Participants will provide a medical history, with special attention to any history of recurrent infections to suggest immune suppression, previous history of splenectomy and prior allergic reactions. In addition, cardiovascular disease risk (defined as > 10%, 5 year risk) will be screened for utilizing a non-laboratory based method. Risk factors include sex, age (years), systolic blood pressure (mm Hg), smoking staus (current vs. past or never), BMI (kg/mm^2^), reported diabetes status, current treatment for raised blood pressure.^58^ They will also undergo physical examination and laboratory screening tests, which include: Hemoglobin, WBC, Platelet count, AST, ALT, creatinine, glucose, hepatitis and HIV serologies, sickle cell trait, urinalysis, and serum pregnancy analysis (if appropriate). The standard for processing blood in commercial laboratory settings is to practice Universal Precautions as matter of course. All blood will be drawn by experienced CVD personnel and will be placed in vacuum-sealed blood tubes for transportation to LabCorp (or the University of Maryland Medical Center as back-up). These sealed blood tubes will be processed by the respective laboratory in a manner which assumes that any blood sample could be contaminated with viruses, bacteria, fungi and parasites. Sera will be banked for troponin analysis. In the event that an asymptomatic individual has evidence of an elevated level, the banked sample will be analyzed to discern whether the result could represent a false positive (0.4% of tests). As it would not be expected to be elevated before malaria challenge intervention, levels will not be used to screen asymptomatic, previously healthy individuals without cardiovascular risk. Finally, a 12-lead ECG will be performed. An abnormal ECG will be defined as showing pathologic Q waves and significant ST-T wave changes; left ventricular hypertrophy; any non-sinus rhythm excluding isolated premature atrial contractions; right or left bundle branch block; or advanced (secondary or tertiary) A-V heart block. A participant who meets any of the exclusion criteria will be excluded. All screening tests will be completed within the 60 days prior to entry into the study but volunteers who are enrolled between Days -60 to -30 will have complete blood counts (CBCs), Biochemistries, and serum pregnancy tests repeated within 30 days of the challenge event so as to maintain accurate safety lab information. Laboratory studies may be conducted at other times during the course of the trial if the investigators judge it necessary for the safety of the participant. All screening and follow-up diagnostic laboratory testing will be drawn at the CVD in Baltimore and sent to commercial testing facilities for analysis. ECGs will be performed and interpreted at the University of Maryland. Recruitment will continue until 18 eligible participants have fulfilled all of the inclusion criteria and none of the exclusion criteria, and signed the study consent form. Part A (5 vs. 3 vs. 1 bite) may then commence. If less than 100% infectivity occurs in volunteers participating in all of the groups of Part A (1*, 3 or 5 bite cohorts) the study will not proceed to Part B (refer to objective of Part A which stipulates the objective of the study is to determine the minimum number of *A. stephensi* bites required to safely achieve 100% adult human volunteer infectivity). * The 1 bite cohort will proceed if study recruitment numbers allow. Part B (20 volunteers) volunteer accrual will commence after completion of Part A volunteer accrual. All 20 volunteers will be enrolled together should the inpatient portion occur at the SNBL. If the inpatient portion is to occur at the GCRC, the volunteers will be split into two groups of 10 volunteers and will commence after 10 volunteers have been recruited. Should participants change their minds and decline to participate prior to the malaria challenge; additional participants will be screened until enough participants are enrolled to meet the requirement of the cohort.

## Enrollment/Baseline

**Day 0:** After meeting eligibility requirements, individual participants will be randomly assigned to receive 1, 3 or 5 bites of aseptically raised mosquitoes infected with the NF54 strain of *P. falciparum* without stratification for gender. Participants will be assigned randomly to three groups* of 6 subjects each (Part A). A second group of 20 individuals (Part B) will be recruited after recruitment is complete for Part A of the malaria challenge study. As stated previously, randomization to the groups will be done using a computer-generated randomization list (See **Section 5.3.1**) It is anticipated that there may be a 20% loss in study participation (following challenge) over the course of the trial allowing for approximately 5 individuals to be analyzed in each of the 1*, 3 and 5 bite cohorts and 16 individuals in Part B. The eligibility criteria will be reviewed on each individual at enrollment to ensure that each individual was properly entered into the study. Repeat pregnancy tests (urine) will be required of all female participants in the absence of written medical proof of sterilization or amenorrhea (defined as > 1 year in duration with medical evaluation) on Day 0 (malaria challenge event). The ramifications of becoming pregnant during the study will be discussed in the informed consent process. Study samples to test pre-challenge ELISA, IFAT, and cellular studies will be drawn (See **Section 8.1.1**). *The 1 bite cohort will proceed if study recruitment numbers allow. This will not affect statistical strength of the study.

### Malaria Challenge

**Day 0:** Malaria challenge is conducted by strapping a container of aseptically-raised mosquitoes infected with the NF54 strain of *P. falciparum* to the forearm of a volunteer and allowing the mosquitoes (5 vs. 3 vs. 1*) to completely engorge.^8, 23^ Mosquitoes used in this study will already have been determined to have a mean of 10,000 sporozoites per paired salivary gland. Each mosquito will feed for approximately 5 minutes and will be dissected immediately to assess the presence of a blood meal and the presence of sporozoites within the glands. If the mosquitoes do not meet these criteria, an additional mosquito(es) will be added until the volunteers receives the specific quantity to which they were randomized to receive (5 vs. 3 vs. 1* bite). The volunteers will be monitored for a minimum of 30 minutes for any evidence of acute reactivity to the bites. Anaphylaxis to mosquito bites is an exceedingly rare event. Nevertheless, access to ACLS equipment and medications such as epinephrine and anti-histamines are available on site. Typically, a depressed ring impression remains upon the skin after removal of the container. Variable reactions occur within this ring of demarcation ranging from mild erythematous, papules to a generalized swelling with confluent erythematous plaques.^8^ These symptoms typically resolve in 24-72 hours and no reports of systemic allergic or anaphylactic reaction have been reported in previous studies. Low dose steroid cream (1% hydrocortisone) will be provided as needed for itching. *The 1 bite cohort will proceed if study recruitment numbers allow.

## Follow-up and Surveillance

**Days 0 to 56:** After malaria challenge, the participant will be observed for local and systemic reactions for a minimum of 30 minutes. Signs and symptoms will be solicited and recorded by the study personnel and/or investigators. Participants will undergo surveillance and follow-up combining outpatient and inpatient approaches to maximize safety, resource utilization and volunteer compliance.

After challenge, we will collect blood as necessary to ensure the safety of volunteers as part of the follow-up. Molecular diagnostic techniques will also be tested (See Tables 5 and 6). Following inoculation of *P. falciparum* parasites via the bite of aseptic mosquitoes, on study day 0, volunteers will be issued symptom diaries and thermometers and instructed to record all symptoms twice daily (8 AM and 8 PM) as well as record baseline temperatures. If they notice any symptoms or evidence of fever, they will be instructed to contact study personnel immediately and will have been provided landline, beeper and cell phone contact numbers. In the event that the volunteer does not have a cell phone, a beeper will be issued for the duration of the study to ensure a means of volunteer contact. In the absence of symptoms, they will be instructed to return for daily outpatient clinical evaluations beginning on study day 5 and continuing to study day 7. They will be asked to maintain their symptom diaries during this outpatient evaluation period. Malaria smears will be performed daily (along with the experimental PCR analysis) during outpatient visits 5-7. On day 8, all volunteers will be admitted to the inpatient ward of the SNBL or GCRC (Table 6). They will have the opportunity to leave the ward and are encouraged to attend to daily activities if they are symptom-free but will be expected to remain at the ward from approximately 7 PM to 7 AM. Daily malaria smears will continue until a positive smear is obtained, at which time the volunteer will undergo directly monitored treatment. Inpatient diagnostic examination includes daily malaria smears and PCR analysis. While they are symptomatic, we request that they remain on the ward while they receive supervised anti-malarial (Chloroquine) therapy for three days. As Chloroquine can rapidly clear parasites, we expect that they will quickly revert to smear-negative status (note: RTQ-PCR analysis is not being performed in real time and will not be used as a criterion for patency). If symptoms develop that are consistent with malaria (i.e., headache, fever, etc.) in blood-smear-negative individuals, blood smears will be performed q 6-8 hours in order to heighten surveillance for the development of malaria. If a positive blood smear is noted, daily CBCs, glucose, creatinine, AST and ALT will be performed for the duration of treatment (until smear negative x 2) and for 4 sequential weekly follow-ups after a positive malaria diagnosis. The volunteer will be discharged after the documentation of two sequential negative blood smears and will receive 3 days of therapy. As part of follow-up for cardiovascular disease risk related to the malaria challenge event, volunteers will have a troponin level and 12-lead ECG performed on day three post-malaria patency and the one week follow-up post-inpatient discharge. The timing of this follow-up is based upon the adverse cardiac event that occurred in a volunteer on day 5 after malaria patency (full description in **Section 2.3.1**). ECGs will first be reviewed by the study investigators. In the absence of electrocardiographic findings, results will be confirmed by a cardiologist within 72 hours. If abnormality is detected, cardiology interpretation and consultation will be sought within 24 hours. Volunteers will be questioned at all follow-ups for the presence of cardiovascular-related signs or symptoms (chest pain or discomfort, shortness of breath, change in exercise tolerance, and palpitations). Any volunteer with cardiovascular symptoms or evidence of abnormal ECGs/troponins will be referred to cardiology for evaluation. If a volunteer has not developed a positive malaria smear by surveillance day 18 and remains symptom free, he/she will be discharged from the inpatient SNBL or GCRC setting and will continue to be followed up daily as an outpatient (Days 19-28) after which he/she will be followed weekly. Follow-up cardiovascular disease risk will not be performed in the absence of malaria patency. It is highly unlikely that a volunteer would be able to transmit malaria via the bite of a mosquito if they remain aparasitemic and asymptomatic. Transmission of malaria requires a gametocytemia. Gametocytemia is detectable in approximately 2.4% of smear positive cases of uncomplicated *P. falciparum* malaria^63^ and rises with environmental stressors such as malaria therapy. While a single schizont develops into either a gametocyte or an asexual stage parasite, the mechanism for this pre-determination is unclear. Evidence of gametocytemia in the setting of a treated, uncomplicated malaria infection is well-described but in the absence of parasitemia or clinical symptoms of malaria, there is no evidence to suggest that gametocytemia occurs in a malaria-naïve host. Clinical evaluations, malaria smears and PCR will be continued at each visit until they develop symptoms, a positive blood smear, or study day 56 is reached. Study samples to include ELISAs, IFAT and ILSDA will be done on Days 14 and 28. PBMC will be obtained on Days 0, 5 and 56. Study day 56 is the last scheduled clinical follow-up.

Every effort will be made to ensure compliance with visits and provide for the safety of the participant. If a participant does not appear for a scheduled clinic visit, attempts will be made to contact the participant and arrange transport to the clinic center. If a serious adverse event (SAE) has occurred, appropriate measures will be taken to notify the CVD independent safety monitor, SMC, all sponsors and IRBs as described in **Section 9.3.1.**

### Malaria Infection

Following administration of *P. falciparum* via the bite of an infected mosquito, volunteers will maintain symptom diaries from Day 0-7. They will be followed as an outpatient from Days 5-7. Volunteers will be admitted to the in-patient clinical ward of the SNBL or GCRC from Days 8-18 (Table 6). Typical patency extends from Days 8-20 but studies conducted at the CVD documented time to infection as being from Day 7-14. The longest recorded patency by blood smear documented in the literature was 20 days with a mean of 10.5 days.^18^ One volunteer was documented to have a self-limited, blood-smear negative, positive malaria culture on Day 21 and 30 following challenge.^24^ Volunteers will be admitted and daily smears and RTQ-PCR assays performed. If symptoms develop that are consistent with malaria (i.e., headache, fever, etc.) in blood-smear-negative individuals, blood smears will be performed q 6-8 hours in order to heighten surveillance for the development of malaria. In the event that clinical malaria infection occurs, volunteers will be treated as an inpatient x 3 days and discharged with weekly follow-up for 4 weeks, having met a primary endpoint of the study. As described in Section 7.3 above, cardiovascular disease risk assessment will occur on day three post-malaria patency and the one week follow-up post-inpatient discharge. Volunteers will be questioned at all follow-ups for the presence of cardiovascular-related signs or symptoms. We do not expect complications or severe events to occur as a result of an experimental malaria challenge but any volunteers who display any symptoms that require more aggressive monitoring than can be offered at the GCRC will be transferred within the University of Maryland Medical Center. The GCRC has access to all acute care interventional equipment and specialists can rapidly be consulted should adverse clinical findings dictate. We would base transfer decisions upon the clinical stability of the patient. Labile vital signs including accelerated respirations indicative of acidosis, hypotension, hyperthermia, evidence of organ compromise such as LFT’s greater than 5X ULN or renal insufficiency or parasitemia unresponsive to Chloroquine therapy are all hypothetical reasons for transfer. Complete blood counts, creatinine, glucose, AST and ALT will be drawn on each day of positive malaria smear, the duration of treatment (if smear-negative before treatment completion) and each weekly (+/- 2 days) (x 4) follow-up thereafter (not required in the event that the volunteer does not acquire malaria). PCR analysis will continue for 2 days after the smear reverts to negative. Participants will receive 1500 mg chloroquine base as malaria treatment over 48 hours (600 mg at time 0 followed by 300 mg oral base at hours 6, 24 and 48), as first line therapy. In the event that the volunteer is taking antacids or kaolin, the chloroquine dosing will be spaced apart from the medication by 4 hours. Second line therapy will be atovaquone/proguanil (Malarone®) at 4 tablets (250/100 mg) orally for three days, representing a total daily treatment dose of 1 gram/400 mg. Ibuprofen and/or acetaminophen will be offered for symptomatic relief of fever or body aches.

The safety of our volunteers is paramount. In the report of Epstein et al.^18^, 62% of volunteers had symptoms for 12 hours before documentation of a positive blood smear. We intend to increase blood smear assessments to every 6-8 hours when there are symptoms consistent with malaria, while closely monitoring for alternate causes of these symptoms (i.e., influenza, gastrointestinal illness, etc.). In individuals with positive symptoms, we will also increase the number of fields read from the standard of 360 fields (1000x) to 720 fields (1000x) to increase the sensitivity by 2-fold. When reading 720 fields the microscopist will be assessing approximately 1.0 ul blood, which means the microscopist will be able to detect 1 parasite/ul blood. Based on a normal value of 4 x10^6^ erythrocytes/ul blood, this means we will be able to detect *P.falciparum* parasites in the bloodstream when 0.000025% of erythrocytes are parasitized (0.000025% parasitemia). Since the definition of severe malaria based on parasitemia is at a minimum > 3% parasitemia and often 10% parasitemia, this means our microscopists will be able detect a level of parasitemia at least 5 orders of magnitude below the cut-off value for severe malaria. In the case of persistent symptoms and negative blood smears (with increased numbers of blood smear per day and examination of 720 high power fields per slide), we will weigh the risk to the volunteer, the extent of symptoms, and clinical stability of the volunteers on an individual basis before instituting anti-malarial therapy. The study objective is to determine the infective dose of mosquito bites at which we can reliably transmit malaria to 100% of volunteers. As such, a false-positive diagnosis is of critical concern in our ability to accurately interpret the results of the challenge study. RTQ-PCR is currently not used as first line diagnostic due to its propensity to have false-positive results. The RTQ-PCR is being studied to objectively measure its utility as a diagnostic assay and to discern the false-positive rate. Careful assessment of the positive and negative predictive value and feasibility of RTQ-PCR in this study may militate for or against its more routine use in future clinical trials. The RTQ-PCR will be performed in batches retrospectively and results will not be shared with investigators until after the study to avoid biasing the primary endpoint measure.

## Final Study Visit

**Day 56 (+/- 2 days):** The final study visit will occur on Day 56 of surveillance (with a range of Day 54-58 for completion). In addition to the described events that will be performed as part of the weekly follow-up appointments, a targeted clinical exam will be performed and a urine pregnancy test will be performed in women of childbearing potential. It is unlikely that *P. falciparum* malaria will emerge after this time period if it has not occurred up to that point, but participants will receive information to contact study coordinators and local emergency care facilities should symptoms consistent with malaria infection develop subsequent to study termination. Queries as to antibiotic usage will be performed to assure that the participants have not consumed antibiotics such as trimethoprim-sulfamethoxazole, azithromycin or doxycycline that could result in suppression of the erythrocytic manifestations of malaria. Information regarding AEs will be solicited at the final scheduled study visit. Any ongoing related AEs will be followed to resolution or until a stable chronic condition has been established. Volunteers will not receive any terminal clearance and will not be treated with antimalarials in the absence of malaria symptoms or malaria smear positivity. Volunteers will receive a 6 and 12 month follow-up phone call. Volunteers will be encouraged to contact study personnel with symptoms consistent with malaria for one year after the initial malaria challenge.

## Early Termination Visit

Every attempt will be made to retain the participants once the trial has commenced. Early termination has special concerns given that the primary outcome of this study is to assess whether *P. falciparum* malaria has been transmitted by a defined quantity of aseptically-raised mosquitoes infected with the NF54 strain of falciparum malaria. While this is ordinarily quite safe and early parasitemias will be caught before significant symptoms are allowed to develop, unmonitored infection can progress to serious illness. Proper participant education at enrollment and stringent follow-up requirements will impress upon the volunteers the seriousness of compliance. Volunteers with any question of reliability will be asked to remain on the inpatient ward for the duration of the in-patient portion of the study, including completion of drug therapy. Participants will wear bracelets or necklace identifiers to inform outside medical personnel that they have been infected with malaria. Volunteers who insist upon terminating the study prematurely will be asked to submit a malaria smear and a PCR analysis. They will be offered therapy to prophylaxis against a malaria infection (atovaquone/proguanil (Malarone®) at 4 tablets (250/100 mg) orally for three days) which has been shown to have hepatic clearing capabilities. Daily telephone calls will be made to volunteers who decide to terminate prematurely. Signs and symptoms consistent with a malaria infection will be elicited. Follow-up care will be offered regardless of study participation if so desired by the participant. In the event that the volunteer refuses drug treatment, they will be contacted daily to assess signs and symptoms related to malaria until Day 28 after which, weekly phone calls will be made until Day 56. The potential for developing malaria wanes considerably after Day 21 of an infective bite and has not been documented in a malaria challenge scenario after this time.

## Unscheduled Visit

The participants will be followed on a daily basis during the high-risk period for malaria development (Days 4-18) with inpatient observation for malaria during days 8-18. Weekly follow-up will continue after that time point. As stated above, participants will wear armbands or necklace identifiers to inform outside medical personnel that they have been infected with *P. falciparum* malaria. Volunteers will maintain symptom diaries during their outpatient surveillance period. Volunteers without cellular phones will be issued beepers to facilitate communication between the investigator and the volunteers. Unscheduled visits will likely represent visits related to the onset of symptom development and will be recorded as an unscheduled visit in the CRF. Malaria smears and a clinical exam will be performed along with biochemistries, a CBC and PCR analysis. A CBC, biochemistry, PCR analysis and malaria smear will continue for the duration of days at which a positive smear continues after which they will be performed on a weekly basis. PCR analysis will end after the second negative malaria smear allowing for the fact that this test is likely to be more sensitive than the malaria smear.

**Table 5: Schedule of Events Surveillance Chart 1**

| **Event** | **Screen**  **Visit** | **Chall-enge** | **Outpatient Visits** | | | | **Inpatient**  **Stay^a^** | **Daily Outpatient Surveillance Visits** | | | | | | | | | | | **Weekly Outpatient Surveillance Visits** | | | | **1 year F/u^m^** |
| --- | --- | --- | --- | --- | --- | --- | --- | --- | --- | --- | --- | --- | --- | --- | --- | --- | --- | --- | --- | --- | --- | --- | --- |
| **Study Visit** | **S1** | **1** | **2** | **3** | **4** | | **5 to 15** | **16** | **17** | **18** | **19** | **20** | **21** | **22** | **23** | | **24** | **25** | **26** | **27** | **28** | **29** |  |
| **Surveillance Day** | **-60 to**  **-1** | **0** | **5** | **6** | **7** | | **8 to 18** | **19** | **20** | **21** | **22** | **23** | **24** | **25** | **26** | | **27** | **28** | **35** | **42** | **49** | **Last Visit**  **56** | 60 to 365 |
| Malaria Challenge |  | X |  |  |  | |  |  |  |  |  |  |  |  |  | |  |  |  |  |  |  |  |
| Informed Consent | X |  |  |  |  | |  |  |  |  |  |  |  |  |  | |  |  |  |  |  |  |  |
| History | X | X | X | X | X | | X | X | X | X | X | X | X | X | X | | X | X | X | X | X | X |  |
| Adverse Events |  |  | X | X | X | | X | X | X | X | X | X | X | X | X | | X | X | X | X | X | X |  |
| Physical Exam^b^ | X | X | X | X | X | | X | X | X | X | X | X | X | X | X | | X | X | X | X | X | X |  |
| Serologies^c^ | X |  |  |  |  | |  |  |  |  |  |  |  |  |  | |  |  |  |  |  |  |  |
| Hematology^d^ | X | X |  |  |  | | Refer |  |  |  |  |  |  |  |  | |  |  |  |  |  |  |  |
| Safety^e^ | X | X |  |  |  | | to |  |  |  |  |  |  |  |  | |  |  |  |  |  |  |  |
| Urinalysis^f^ | X |  |  |  |  | | Chart 2 |  |  |  |  |  |  |  |  | |  |  |  |  |  |  |  |
| Pregnancy Test^g^ | X | X |  |  |  | |  |  |  |  |  |  |  |  |  | |  |  |  |  |  | X |  |
| Malaria Smear^h^ |  | X | X | X | X | | X | X | X | X | X | X | X | X | X | | X | X | X | X | X | X |  |
| PCR Analysis^i^ |  | X | X | X | X | | X | X | X | X | X | X | X | X | X | | X | X | X | X | X | X |  |
| Study Samples^j^ |  | X | X |  | X | |  |  |  |  |  |  |  |  |  | |  | X |  |  |  | X |  |
| Antibiotic Assays^k^ |  | X |  |  | X | |  |  |  | X |  |  |  |  |  | |  | X |  |  |  |  |  |
| Total Blood (mL) | 30 | 77 | 50 | 3 | 10 | | 73-93^n^ | 3 | 3 | 3 | 3 | 3 | 3 | 3 | 3 | | 3 | 13 | 3 | 3 | 3 | 90 ^n^ |  |
| Cumulative Blood |  | 107 | 157 | 160 | 170 | | 243-263 | 246-66 | 249-69 | 25272 | 255-75 | 258-78 | 261-81 | 264-84 | 267-87 | | 270-90 | 283-303 | 286-06 | 289-09 | 292-12 | 382-402 | 382-402 |
| SMC Meeting^l^ |  |  |  |  |  | |  |  |  |  |  |  |  |  |  | |  |  |  |  |  | X |  |
| ^a^ See Chart 2. In the absence of a positive malaria smear and symptoms by surveillance day 18, participants will be discharged for follow-up as an outpatient (Chart 1)  ^b^ Targeted exam includes vital signs  ^c^  Serologies: HIV, Hepatitis, Sickle cell trait.  ^d^ Complete blood count (CBC) will also be drawn on all days of positive malaria smear, the duration of treatment and at each weekly follow-up (x 4) thereafter.  ^e^ Creatinine, glucose, AST and ALT will be drawn on all days of positive malaria smear, the duration of treatment and each weekly follow-up (x 4) thereafter. ECG and troponin will be done at screening (sera to be banked) | | | | | | post-patency day 3 and weekly visit 1 post-malaria Rx. ^f^  Urinalysis refers to urine dipstick  Serum pregnancy test (βhCG) will be done at screening. Urine pregnancy tests will be done at enrollment pre-malaria challenge, on the day of the first positive malaria smear, and at the last visit (Visit 29).  ^h^ Malaria smear refers to thick blood smear obtained weekly x 4 after completion of malaria therapy.  ^i^  Blood samples for research parasite DNA PCR will end after the third negative malaria smear following antimalarial therapy initiation.  ^j^  Study samples archived for the purpose of ELISA and IFAT (10 mL) analysis will be drawn on D0, 14, 28. 40- | | | | | | | | | | 50 mL will be drawn D0, 5 and 56 for PBMC preservation and 7 mL on D0, 5, 7 for microarray.  ^k^ Antibiotic assay will be performed in urine to detect the presence of occult antibiotic presence (Days 0, 7, 14 ,28)  ^l^  The independent safety monitor and safety monitoring committee will convene within 5 weeks (56-91) of Part A and B to review the previous 56 days of safety history, laboratories and adverse events.  ^m^ One year passive surveillance to assure that no malaria or complications develop. Participants will be contacted by phone at months 6 and 12.  ^n^ With malaria infection, 10 mL of blood drawn for each day of smear positivity and for 4 sequential weeks in f/u. | | | | | | | |

**Table 6: Schedule of Inpatient Stay and Malaria Event Surveillance Chart 2**

| **Event** | **Inpatient Surveillance^a^** | | | | | | | | | | | | | | |
| --- | --- | --- | --- | --- | --- | --- | --- | --- | --- | --- | --- | --- | --- | --- | --- |
| **Study Visit** | **5** | **6** | **7** | | **8** | **9** | **10** | **11** | **12** | **13** | | **14** | **15** | **Smear Positive Event** | **Weekly**  **F/u** **(x 4)^m^** |
| **Surveillance Day^b^** | **8** | **9** | **10** | | **11** | **12** | **13** | **14** | **15** | **16** | | **17** | **18^c^** | **Variable** | **Variable** |
| Admission | X |  |  | |  |  |  |  |  |  | |  |  |  |  |
| History | X | X | X | | X | X | X | X | X | X | | X | X | X | X |
| Adverse Events | X | X | X | | X | X | X | X | X | X | | X | X | X | X |
| Physical Exam^d^ | X | X | X | | X | X | X | X | X | X | | X | X | X | X |
| Hematology^e^ |  |  |  | |  |  |  |  |  |  | |  |  | X | X |
| Safety^f^ |  |  |  | |  |  |  |  |  |  | |  |  | X | X |
| Pregnancy Test^g^ |  |  |  | |  |  |  |  |  |  | |  |  | X |  |
| Malaria Smear^h^ | X | X | X | | X | X | X | X | X | X | | X | X | X | X |
| PCR Analysis^i^ | X | X | X | | X | X | X | X | X | X | | X | X | X | X |
| Study Samples^j^ |  |  |  | |  |  |  | X |  |  | |  |  | X |  |
| Antibiotic Assays^k^ |  |  |  | |  |  |  | X |  |  | |  |  |  |  |
| Total Blood (mL) | 3 | 3 | 3 | | 3 | 3 | 3 | 13 | 3 | 3 | | 3 | 3 | 30-50^l^ | 10 |
| Cumulative Blood (mL) | 3 | 6 | 9 | | 12 | 15 | 18 | 31 | 34 | 37 | | 40 | 43 | 73-93^l^ | 113-133 |
| ^a^ If malaria develops between Days 8-18, the patient will be treated as an inpatient x 3 days and discharged with weekly follow-up x 4, having met a primary endpoint.  ^b^ The Surveillance Day derives from each day post-malaria challenge  ^c^  In the absence of a positive malaria smear and symptoms by surveillance day 18, participants will be discharged for follow-up as an outpatient.  ^d^ Targeted exam including vital signs (heart rate, blood pressure, respiratory rate, temperature)  ^e^ Complete blood count (CBC) will also be drawn on all days of positive malaria smear, the duration of treatment and at each weekly follow-up thereafter. | | | | ^f^ Creatinine, glucose, AST and ALT will be drawn on all days of positive malaria smear, the duration of treatment and each weekly follow-up thereafter. ECG and troponin will be done at post-patency day 3 and weekly visit 1 post-  malaria therapy/inpatient discharge.  ^g^ Urine pregnancy test will be done on the day of the first positive malaria smear  ^h^ Malaria smear refers to thick blood smears to be drawn once daily during the inpatient stay, each day of positive malaria smear, the duration of treatment and at each weekly follow-up thereafter. Inpatient symptomatic volunteers will have q 6-8 hour smears to assess for malaria. | | | | | | | ^i^  Blood samples for research parasite DNA PCR will end after the third negative malaria smear following antimalarial therapy initiation.  ^j^  Study samples refers to research sample (10 mL) drawn on Day 14 and archived for the purpose of ELISA analysis and IFAT.  ^k^ Antibiotic assay will be performed in urine to detect the presence of occult antibiotic presence  ^l^ Biochemistries and CBC will be drawn on all days of positive malaria smear and the duration of treatment (3-5 d). This is anticipated to be 30-50 mL in volunteers who contract malaria. Blood smears and PCR will be done via venous blood draw.  ^m^Weekly f/u (+/- 2 days) will be as outpatients. This will likely correspond to Days 21, 28, 35 and 42. Biochemistries, CBC, smear and PCR to be done. | | | | |

# Study Procedures/Evaluations

## Clinical Evaluations

The following study procedures will occur both for Malaria Challenge, Part A and Part B. Part B will occur within 35 days of the completion of the Part A, Day 60 follow-up appointment.

### Screening and enrollment:

#### Day –60 to –1: Screening and inclusion of participants

- Eligible participants will be issued general information pertaining to the study including purpose and study procedures. Interested parties will be asked to sign a screening consent form. The following procedures will follow only after a signed consent form is obtained.
- A review of inclusion and exclusion criteria will be performed.
- A general medical history including targeted questions as to previous allergic reactions, travel history, history of splenectomy and/or sickle cell trait and cardiovascular disease risk assessment will be performed.
- A screening 12-lead ECG will be performed
- A medical exam with vital signs will be performed
- All current medications will be reviewed by study personnel
- A 10 mL sample of venous blood for safety evaluation (CBC, Biochemistries (creatinine, glucose, AST and ALT), and pregnancy tests (for all females unless written medical confirmation of sterility or amenorrhea (defined as greater than 1 year with medical evaluation) is provided) will be obtained from all study participants. Sera will be banked for retrospective troponin levels. Results will be considered valid within 30 days of study enrollment and will be repeated in the event that the volunteer was enrolled between Days -60 and -31.
- A 20 mL sample of venous blood will be obtained for viral serologies (HIV, HbsAg, HCV), and sickle cell trait.
- A urinalysis will be performed (dipstick) on each individual with results considered valid within 30 days of study enrollment. In the event that the volunteer was enrolled between Days -60 and -31, the urinalysis will be repeated.

#### Day 0: Malaria Challenge

#### Prior to the malaria challenge:

- All screening laboratories and ECG interpretation(s) will be reviewed
- Eligibility criteria will be reviewed
- Interim (previous 2 week) antibiotic usage will be reviewed.
- A unique study alpha numeric identifier will be assigned and the participant will be randomized to the 1*, 3 or 5 bite cohorts of Malaria Challenge, Part A or Malaria Challenge, Part B. *The 1 bite cohort will proceed if study recruitment numbers allow.
- Urine will be collected on all females to perform a urine β-HCG pregnancy test (unless written medical confirmation of sterility or amenorrhea (defined as greater than 1 year with medical evaluation) is provided)
- Urine will be banked on all individuals for an antibiotic assay to test for the presence of antibiotics that may act to suppress parasite growth (in those individuals who do not acquire malaria infection).
- 77 mL of venous blood will be collected. 10 mL will be used to perform CBC, Biochemistries (creatinine, glucose, AST and ALT), malaria smear, and PCR analysis. 67 mL will be archived for research purposes (i.e., ELISA and IFAT (10 mL) and 50 mL for PBMC preservation for the purposes of cellular studies and (7 mL) for gene expression (microarray) analyses).
- Vital signs will be recorded (including an oral temperature, blood pressure, heart rate and respiratory rate).
- An updated history and a physical (minimum physical exam includes HEENT, cardiac, pulmonary, abdominal exam, and extremities) will be performed.
- Participants will be issued bracelets or necklaces identifying them as having been exposed to malaria. If they do not own a cell phone, a beeper will be issued to facilitate contacting study personnel.
- The malaria challenge will be administered.
- After challenge, the participants will be observed for a minimum of 30 minutes.
- Oral temperature, blood pressure, and pulse will be recorded.
- All solicited and unsolicited events will be recorded.
- Participants will be instructed to maintain a symptom diary for 7 days and to record symptoms and temperature twice daily (8 AM +/- 2 hours and 8 PM +/- 2 hours). The card and instructions will be given on the day of malaria challenge.
- Participants will be instructed to contact study personnel immediately should they manifest any signs or symptoms they perceive as serious.
- Participants will be counseled on mosquito avoidance behavior.

### Subject follow-up:

#### Days 1-4, Outpatient Post-malaria Challenge Surveillance

- Participants will be instructed to maintain a symptom diary and to record symptoms and temperature twice daily (8 AM +/- 2 hours and 8 PM +/- 2 hours).
- Participants will be instructed to contact study personnel immediately should they manifest any signs or symptoms they perceive as serious.
- In the absence of symptoms, participants will be instructed to return for daily clinical evaluations beginning on study day 5.

#### Days 5-6, Outpatient Post-malaria Challenge Surveillance Visits

- Participants will be interviewed. Symptom diaries will be reviewed and solicited and unsolicited signs and symptoms through Day 4 post-malaria challenge will be recorded.
- A review of all current medications (focusing on antibiotics) will be conducted
- Vital signs and a targeted clinical exam with special attention to the site of malaria challenge will be conducted
- 50 mL of venous blood will be collected on Day 5. 2-3 mL will be used to perform a malaria smear and PCR analysis. 47 mL will be archived for research purposes (i.e., 40 mL for PBMC preservation for the purposes of cellular studies and 7 mL for gene expression (microarray) analyses).
- 3 mL of venous blood will be collected on Day 6. 2-3 mL will be used to perform a malaria smear and PCR analysis.
- Participants will be counseled on mosquito avoidance behavior.

#### Day 7, Outpatient Post-malaria Challenge Surveillance

- Participants will be interviewed. Symptom diaries will be reviewed and solicited and unsolicited signs and symptoms will be recorded.
- A review of all current medications (focusing on antibiotics) will be conducted.
- Vital signs and a targeted clinical exam will be conducted.
- 10 mL of venous blood will be collected. 2-3 mL will be obtained for malaria diagnostics (malaria blood smear and PCR analysis) and 7 mL for gene expression (microarray) analysis.
- Urine will be banked for an antibiotic assay to test for the presence of antibiotics that may act to suppress parasite growth (to be performed only in those individuals who do not subsequently acquire malaria infection).
- Participants will be counseled on mosquito avoidance behavior.

#### Days 8-18, Inpatient Daily Post-malaria Challenge Surveillance

- Participants will be admitted to the Shin Nippon Biomedical Laboratories (SNBL) (or General Clinical Research Center (GCRC)) for inpatient observation during the period of time they are most at risk for the development of falciparum malaria
- The admission will be open in that participants will be able to leave the ward after blood draws from ~7 AM to 7 PM to attend to daily activities. They will be asked to sleep on the ward.
- Participants will be interviewed. Solicited including cardiac symptoms (chest pain or discomfort, shortness of breath, change in exercise tolerance, and palpitations) and unsolicited signs and symptoms will be recorded.
- A review of all current medications (focusing on antibiotics) will be conducted.
- Vital signs and a targeted clinical exam will be conducted.
- A small quantity of blood (2-3 mL) will be obtained daily for malaria diagnostics (malaria blood smear and PCR analysis). Symptomatic individuals will have smears performed q 6-8 hours for the purposes of malaria diagnosis.
- 10 mL of venous blood will be collected on all days of a positive malaria smear and for the duration of treatment. The sample will be used to perform a CBC, Biochemistries (creatinine, glucose, AST and ALT), malaria smear, and PCR analysis as well as a troponin level on post-patency day 3.
- 10 mL of venous blood will be collected on day 14 and archived for the purpose of ELISA analysis and IFAT.
- On day 14, urine will be banked for an antibiotic assay to test for the presence of antibiotics that may act to suppress parasite growth (in those individuals who do not acquire malaria infection).
- Urine will be collected on all females to perform a urine β-HCG pregnancy test (unless written medical confirmation of sterility or amenorrhea (defined as greater than 1 year with medical evaluation) is provided) on the day of the first positive malaria smear.
- If by Day 18, the study volunteer has not contracted malaria and is free of signs and symptoms of malaria, they will be discharged from the inpatient setting and will continue with daily clinical evaluations and malaria smears. Participants will resume maintenance of symptom diaries with twice daily temperature and symptom recordings.

#### Days 19-28, Outpatient Daily Post-malaria Challenge Surveillance

- Participants will be interviewed. Symptom diaries (for those who do not acquire malaria) will be reviewed and solicited and unsolicited signs and symptoms will be recorded.
- A review of all current medications (focusing on antibiotics) will be conducted.
- Vital signs and a targeted clinical exam will be conducted.
- A small quantity of blood (2-3 mL) will be obtained daily for malaria diagnostics (malaria blood smear and PCR analysis).
- 10 mL of blood will be obtained on Day 28 for research purposes (i.e., ELISAs and IFAT.
- On day 21 and 28, urine will be banked for an antibiotic assay to test for the presence of antibiotics that may act to suppress parasite growth (in those individuals who do not acquire malaria infection).
- If the participant remains malaria-free until Day 28, they will continue to be followed on a weekly basis (See **8.1.2.6** below)
- Participants will be counseled on mosquito avoidance behavior.

#### Days 29-56, Outpatient weekly (x4 weeks (+/- 2 days)) Post-malaria Challenge Surveillance

- Participants will be interviewed. Symptom diaries will be reviewed and solicited and unsolicited signs and symptoms will be recorded.
- A review of all current medications (focusing on antibiotics) will be conducted.
- Vital signs and a targeted clinical exam will be conducted.
- Venous blood (2-3 mL) will be drawn for the purposes obtaining a malaria blood smear and PCR analysis at outpatient visits 26-29. In the event that volunteers have acquired a malara infection and complete their 4 sequential weekly follow-up visits prior to Day 56, they will receive one final malaria blood smear and PCR analysis at this study visit.
- 47 mL of blood will be obtained on Day 56 (+/- 2 days) for research purposes (i.e., PBMC (40mL) for immunoassays and (7 mL) for gene expression (microarray)).
- Urine will be collected on Day 56 on all females to perform a urine β-HCG pregnancy test (unless written confirmation of sterility or amenorrhea (defined as greater than 1 year) is provided).
- Beepers will be collected on Day 56 (range Days 54-58) from those individuals to whom one had been issued.
- Active study surveillance will be terminated after Day 56 (range Day 54 to 58).
- Study participants will be contacted by telephone at 6 and 12 months for follow-up.
- Study participants will be instructed to contact study personnel with symptoms consistent with malaria for one year post-challenge.

#### Malaria infection (Predicted to occur between Days 8-18)

- Participants will be interviewed. Solicited and unsolicited signs and symptoms will be recorded.
- A review of all current medications (focusing on antibiotics) will be conducted.
- Vital signs and a targeted clinical exam will be conducted. Vital signs will be monitored q 4-6 hours.
- 10 mL of venous blood will be collected. The samples will be used to perform CBC, Biochemistries (creatinine, glucose, AST and ALT), and a malaria smear. A troponin will be performed on post-patency day 3. These laboratories will be drawn on a daily basis as long as the malaria smear remains positive or for the duration of malaria therapy if the parasitemia becomes undetectable.
- The PCR analysis will be performed daily for the duration of the positive malaria smear and continue for two days after resolution of the visually detectable blood-stage parasitemia.
- Urine will be collected on all females at the time of malaria smear positivity to perform a β-HCG pregnancy test.
- Participants will receive 1500 mg chloroquine base/2500 mg salt as malaria treatment over 48 hours (600 mg base/1000 mg salt at time 0 followed by 300 mg oral base/500 mg salt at hours 6, 24 and 48), as first line therapy. Second line therapy will be atovaquone/proguanil (Malarone®) at 4 tablets (250/100 mg) orally for three days, representing a total daily treatment dose of 1 gram/400 mg.
- Ibuprofen at 200-600 mg q 6 hours and/or acetaminophen at 325-500 mg orally q 4-6 hours will be administered as needed to relieve symptoms such as fever, headache and body aches.
- Study participants will be discharged after completion of oral antimalarial therapy x 3 days and the documentation of two sequentially negative malaria smears. They will be asked to follow-up on a weekly basis x 4 weeks (+/- 2 days).
- A 12-lead safety ECG will be obtained on post-patency day 3 and at the first post-treatment weekly follow-up
- 10 mL of venous blood will be collected at each of 4 post-treatment weekly (+/- 2 days) follow-ups (This may or may not coincide with Visits 26-29 which is the weekly follow-up visit schedule for individuals who do not get malaria. Refer to Chart 2 for Schedule of Inpatient Stay and Malaria Event). The samples will be used to perform CBC, Biochemistries (creatinine, glucose, AST and ALT), and a malaria smear as well as a troponin level for post-treatment weekly follow-up 1.

## Laboratory Evaluations

### Clinical Laboratory Evaluations

**Screening:**

The following laboratory tests will be performed at the time of participant screening and will be reviewed prior to enrollment into the protocol. Each laboratory result will be compared to upper and lower limit norms for the University of Maryland laboratory system. Results will be categorized as normal meaning that the values were within standard limits determined based on local population results for the UMD reference laboratories, or abnormal/positive based on outlying values beyond the upper or lower limit of normality. The University of Maryland Reference laboratories (or LabCorp facilities), will perform these assays. It is estimated that 10 mL will be sufficient to complete the biochemical and hematological analysis. Additionally, 20 mL will be sufficient to complete viral serologies and sickle cell trait analysis.

**Hematology***:* hemoglobin, white blood cells (WBC) and, platelet count.

**Biochemistry**: Glucose, renal function test (creatinine), and liver enzymes (AST [Aspartate aminotransferase], and ALT [Alanine aminotransferase]).

**Cardiac enzymes**: Sera for a troponin level will be banked at the CVD at screening and performed at the University of Maryland Medical System at defined time points (day 3 post-patency and one week post-malaria therapy) in follow-up.

**Viral serologies:** Anti-Hepatitis C virus (HCV), Hepatitis B surface antigen (HbsAg) and Human Immunodeficiency Virus (HIV) will be recorded at baseline

**Sickle Cell Trait:** Hemoglobin electrophoresis screening for sickle cell trait will be recorded at baseline.

**Urinalysis:** A dipstick test will be performed examining urine for the presence of protein or blood. Trace values will be accepted but values quantified as “moderate” and/or > 1+ will be deemed abnormal. (Refer to Table 10)

**Enrollment:**

In addition to hematology, cardiac enzymes and biochemistry that will be repeated at scheduled intervals; the following assays will be performed and repeated according to the schedule outlined in Tables 5 and 6.

**Pregnancy Test:** A serum pregnancy test will be performed at the clinical trial study site at the time of screening on all female participants regardless of age unless written medical demonstration of sterility or amenorrhea (defined as one year with medical evaluation) can be provided. A Food and Drug Administration (FDA)-approved testing kit for urine β-hCG pregnancy testing will be performed at the clinical trial study site on all female participants within 24 hours of the malaria challenge study. In addition, testing will be performed on the first day of a positive malaria smear and again on Visit 29 (Day 54-58) at the conclusion of active surveillance.

**Blood malaria smear**: A small aliquot of blood (10 μL) will be placed upon microscope slides for the creation of thick malaria smears. The thick smear will be allowed to dry, lysed with distilled water and stained with Giemsa for analysis of intra-erythrocytic ring forms consistent with malaria. Investigators will examine 300 high-power fields (1000x) of thick blood smear (this represents approximately 0.75 µl of blood) with the quantity of parasite per μL determined utilizing the amount of parasites detected per 300 WBC’s visualized (assuming a mean WBC of 7,500 cells/μL) or the amount of parasites visualized in a specific aliquot of blood (i.e., number of parasites per 10 µl of blood). This will be done by counting 500-1000 RBCs and reporting the percentage of infected RBCs per 100 RBCs (0.5%, 1.0% etc.) See Table 6 for quantification of parasitemia. The peripheral blood smear provides comprehensive information on the stages, and the density of parasitemia with a sensitivity of 5 to 20 parasites/µL of blood for an experienced laboratory professional although this level can be much higher taking into account reader variability. These assays will be performed on-site in the Malaria Laboratory and Insectary of the CVD or at facilities created in the SNBL or the GCRC at the University of Maryland Medical Center. Two researchers in the CVD Malaria Unit “screeners” who have extensive experience in the reading of malaria blood smears and/or personnel who have undergone a slide-reading seminar will screen, read and quantify parasite burden. For positive smears, or if questions or discrepancies arise, a third investigator “expert” will be on hand to read and quantify parasite burden with the benefit of a higher level of slide reading expertise. Investigators will take part in a training workshop prior to study initiation to practice blood smear reading techniques and familiarize them with a Malaria Smear SOP to ensure identical procedure and technique. The “expert” reader will participate in an intensive training workshop with a much greater level of stringency in order to eliminate any and all false positive blood smear reading in the study. The minimum acceptance criteria is NO false positive reads on thick smears and a positive smear will be defined as two unquestionable parasites present on smear. The above technique has proven to be highly effective and accurate with 45% of individuals being diagnosed prior to malaria symptoms (personal communication, Ted Hall, WRAIR). The slides will be stored for future review as necessary.

**Table 7: Parasitemia determined by light microscopy as adapted from Hanscheid.^64^**

| **Parasitemia^a^** | **Parasites/μL^b^** | **Correlation** |
| --- | --- | --- |
| 0.0001-0.0004% | 5-20 | Examine 300 HPF^c^ to assure 0.75 μL is reviewed. |
| 0.002% | 100 | Threshold at which clinical symptoms can be seen |
| 0.2% | 10,000 |  |
| 2% | 100,000 |  |
| 2-5% | 100,000-250,000 | Hyperparasitemia, severe malaria |
| 10% | 500,000 | Exchange transfusion may be considered |

**^a^** Determined by thin smear analysis ^b^ Determined by thick smear analysis ^c^  HPF = High power field

### Special Assays or Procedures

- **12-lead ECG:** A 12-lead ECG will be performed at screening prior to enrollment for the purposes of evaluating for occult cardiovascular disease. In addition to the screening ECG, repeat ECGs will be performed in individuals who develop patent malaria infection at post-patency day three and at the first post-treatment weekly follow-up to evaluate for cardiovascular disease. All ECGs will be evaluated first by clinical investigators and subsequently by a board certified cardiologist at the University of Maryland. Screening ECGs will be interpreted by cardiology within 7 days. Follow-up ECG results will be interpreted by a cardiologist within 72 hours in the absence of electrocardiographic findings. If an abnormality is detected, cardiology interpretation and consultation will be sought within 24 hours. An abnormal ECG will be defined as follows: pathologic Q waves and significant ST-T wave changes; left ventricular hypertrophy; any non-sinus rhythm excluding isolated premature atrial contractions; right or left bundle branch block; or advanced (secondary or tertiary) A-V heart block.
- **Research serology:** An aliquot of blood (10 mL) will be drawn at the time of enrollment and at periodic points during study surveillance (pre-challenge, and on day 14 and 28, after challenge). These sera will be assessed by enzyme linked immunosorbent assay (ELISA) for antibodies to the circumsporozoite protein. liver stage antigen 1, erythrocyte binding antigen 175, and merozoite surface protein 1 and possibly apical membrane antigen 1 (PfCSP, PfLSA-1, PfEBA-175 and PfMSP-1 and possibly PfAMA1), and by indirect fluorescent antibody test for antibodies to *P. falciparum* sporozoites, late liver stage parasites, asexual and sexual erythrocytic stages. These studies will be done for research purposes only and will have no bearing upon the clinical management of volunteers who contract malaria.
- **Research Peripheral Blood Mononuclear Cells (PBMC):** An aliquot of blood (50 mL) will be drawn at the time of enrollment and at periodic points (40 mL for Days 5 and 56) during study surveillance (pre-challenge, and on day 5 and 56 (+/- 2 days) after challenge). PBMC will be separated and cryopreserved. PBMC will be used for the purposes of immunoassays and multi-channel intracellular staining by flow cytometry and other techniques to assess immune responses in those individuals who do and do not acquire malaria infection. These studies will be done for research purposes only and will have no bearing upon the clinical management of volunteers who contract malaria. Samples will remain and be stored in liquid nitrogen vapor phase at facilities at the CVD. Funding for pursuing these studies will be garnered via other mechanisms and not through the VTEU.
- **Research microarray (Gene Expression Profiling) from whole blood RNA:** Microarray analysis will be utilized to determine whether upregulation of any specific genes is correlated with protection against challenge. Timepoints coincide with planned follow-up visits to assess safety. Day 5 and 7 post challenge was chosen based upon our knowledge of the *P. falciparum* life cycle. Within 72 hours after the invasion of sporozoite invasion of hepatocytes, parasites express novel hepatocyte stage-specific antigens which may be the key components for eliciting pre-erythrocytic stage protection. In order to capture the immune response elicited by these novel hepatocyte antigens, we wish to perform the microarray analysis using the timepoint of 5 and 7 days post challenge (recognizing that 3 days would be ideal although no scheduled volunteer follow-up occurs at that time) and compare this to baseline Day 0. RNA will be extracted from whole blood from which cDNA can be constructed. Labeled targets can then be hybridized to microarray cassettes incorporating the pre-selected genes of interest and the signals read. Data will be processed to investigate whether there is an expression signature that correlates with protection.
- **PCR analysis:** A sensitive real-time polymerase chain reaction (PCR) will be used for the detection of *P. falciparum* parasites. As we intend on quantifying parasitemia, PCR will be run off venous blood acquired in EDTA tubes. Optimization experiments have established a high degree of sensitivity. This technique has been shown to reliably detect low numbers of *P. falciparum* parasite (i.e., 10 parasites/mL of blood).^48^ Due to false positive sample results, malaria smears will be the gold standard of parasite detection with PCR performed to validate traditional techniques and to confirm smear positive diagnoses. These assays will be performed in the malaria laboratory at the Center for Vaccine Development. This laboratory has extensive experience in falciparum PCR analysis and routinely processes samples from around the world.
- **Antibiotic Assay:** Urine will be banked for the purposes of performing antibiotic assays in individuals who do not acquire clinical malaria post-challenge. Urine will be obtained and archived on Day 0, 7, 14 and 28. It is unlikely that a malaria event will occur past this time period. This is a biological assay for occult antibacterial activity using a lawn of a bacillus strain on agar as the indicator. The assays were used to determine adherence to antibiotic dosing regimens in pediatrics and also for continuous ambulatory peritoneal dialysis patients. The procedure is qualitative, i.e. antibacterial activity or no activity. The antibiotic is not identified. This method has been used in prior malaria challenge events that occurred at the CVD and will be performed as a research assay at the University of Maryland Medical System Microbiology Laboratory by Dr. Richard Venezia.

### Specimen Preparation, Handling, and Shipping

#### Instructions for Specimen Preparation, Handling, and Storage

Blood will be collected from study participants by venipuncture up to 11 times during the study, including screening. Additionally, fingerpricks will be performed on at least 9 occasions (range 9-24) depending upon the time of malaria onset. The maximum amount of blood requested from any participant for standard collection during the study for research purposes will not exceed 190 mL over the study period. However, additional blood may be obtained as deemed necessary by the investigators or clinicians to evaluate any illness or condition. Blood will be obtained by research staff as part of the clinical evaluation of the participant. Assays will be sent to the University of Maryland central laboratory processing or onto LabCorp diagnostics depending on the nature of the testing. Research samples will be processed and stored at -80°C at the Center for Vaccine Development prior to transfer to the Sanaria, Inc. laboratories. In the case of PBMCs for cellular studies and microarray, the cell samples will be stored (or flash frozen in the case of the microarray) in liquid nitrogen vapor phase in storage facilities at the CVD. Urine samples for antibiotic testing will likewise be stored at the Center for Vaccine Development. These will only be tested in individuals who do not develop malaria after an appropriate malaria challenge. Dr. Richard Venezia of the University of Maryland, Division of Microbiology will be performing these research assays. RTQ-PCR assays will be run at the Center for Vaccine Development.

#### Specimen Shipment

Samples may be delivered to the Sanaria facilities for research assays such as ELISAs and indirect florescence antibody testing. PBMC will be cryopreserved and remain at the University of Maryland. RNA from these cells may be shipped as part of a microarray analysis study to assess early genetic responses to malaria infection. Sera will be maintained at -20°C or less whereas RNA can be transported at room temperature. Sanaria is located approximately 35 miles from the University of Maryland CVD in Baltimore at 9800 Medical Center Drive, Rockville, MD 20850, Contact: Stephen L. Hoffman, M.D., Tel: 301-770-3222, Fax: 301-770-5554. Study personnel will hand-deliver samples to assure the integrity. Specimen-tracking logs will be maintained.

# Assessment of Safety

## Specification of Safety Parameters

The primary safety measurements of this trial are:

1. Occurrence of solicited symptoms after malaria challenge (and before occurrence of a positive blood smear) during a 7-day surveillance period (day of challenge (Day 0), days 1, 2 and 3 after challenge, and day 5-7 visits)
2. Occurrence of solicited symptoms to a malaria event (recorded at each day of inpatient observation during the malaria event and once weekly as part of the standard outpatient malaria event follow-up). The malaria event is defined as the presence of a positive blood smear.
3. Occurrence of solicited and unsolicited symptoms after the malaria challenge during a 56-day surveillance period (day of challenge and 55 subsequent days)
4. Occurrence of serious adverse events throughout the study period of 365 days. Special attention will be paid to cardiac signs and symptoms post-malaria challenge.

## Methods and Timing for Assessing, Recording, and Analyzing Safety Parameters

### Adverse Events

An adverse event includes any noxious, pathological or unintended change in anatomical, physiological or metabolic functions as indicated by physical signs, symptoms and/or laboratory-detected changes occurring in any phase of the clinical study whether associated with the study product and whether or not considered related to the intervention. This definition includes an exacerbation of pre-existing conditions or events, intercurrent illnesses, or drug interaction. While smear-positive (either symptomatic or asymptomatic) malaria infection is a primary outcome measure of this study and is expected given the nature of the parasite challenge, we will document solicited symptoms incurred as a result of the malaria event and code them as adverse events to analyze whether uncomplicated malaria induced by aseptically-raised parasites has clinical characteristics that differ with standard uncomplicated malaria events. We have no reason to believe that malaria infection as a result of aseptic challenge events is any different than malaria acquired through the traditional challenge method. We will carefully gather data in order to verify this hypothesis. Severe malaria infection (i.e., life-threatening) will be reported as an SAE throughout the 56 day period of active surveillance as well as throughout the entire 365 day passive follow-up. Anticipated day-to-day fluctuations of pre-existing conditions that do not represent a clinically significant exacerbation need not be considered adverse events. Discrete exacerbations of chronic conditions that are deemed to be different than regularly sustained day-to-day fluctuations, occurring during a study period will be reported as adverse events in order to assess changes in frequency or severity.

Adverse events will be documented in terms of a medical diagnosis. When this is not possible, the adverse event will be documented in terms of signs and/or symptoms observed by the investigator or reported by the subject at each study visit. All AEs occurring while on study will be documented appropriately regardless of relationship. Pre-existing conditions or signs and/or symptoms (including any which are not recognized at study entry but are recognized during the study period) present in a participant prior to the start of the study will be recorded on the participant’s CRF and will be recorded as an AE if deterioration or exacerbation in the condition occurs during the study. Any hospitalization other than the planned inpatient evaluation from Days 8-18 will be considered a serious adverse event. Information to be collected includes event description, date of onset, clinician’s assessment of severity, relationship to study product (assessed only by those with the training and authority to make a diagnosis, which would include a MD, PA, Nurse Practitioner, DO or DDS), and date of resolution/stabilization of the event. All AEs will be followed to adequate resolution or stabilization. Solicited adverse events to be recorded as endpoints are described below in Table 8 and 9. Table 8 summarizes solicited events to the malaria challenge event while Table 9 refers to solicited events sustained as a result of a smear-positive malaria event. Note: solicited symptoms should be recorded on the diary cards unless they continue past the surveillance time frame (challenge solicited AEs go beyond day 7). As we expect some mild systemic symptoms to occur as a result of mosquito challenge, a solicited challenge site symptom such as erythema, induration or swelling will be recorded as an AE if it continues more than 48 hours after the malaria challenge event. Events that occur prior to 48 hours will be collected but will not be deemed as a challenge site AE unless noted for more than 48 hours after the malaria challenge event. All other adverse events will be recorded as unsolicited adverse events. All AEs must be graded for severity and relationship to study product.

**Severity of Event:** All AEs will be assessed by the clinician using a protocol defined grading system. For events not included in the protocol defined grading system, than the following guidelines will be used to quantify intensity.

- Mild: events require minimal or no treatment and do not interfere with the patient’s daily activities.
- Moderate: events result in a low level of inconvenience or concern with the therapeutic measures. Moderate events may cause some interference with functioning.
- Severe: events interrupt a patient’s usual daily activity and may require systemic drug therapy or other treatment. Severe events are usually incapacitating.
- Life-threatening: any adverse drug experience that places the patient or subject, in the view of the investigator, at immediate risk of death from the reaction as it occurred, i.e., it does not include a reaction that had it occurred in a more severe form, might have caused death.

Changes in the severity of an AE should be documented to allow an assessment of the duration of the event at each level of intensity to be performed. Adverse events characterized as intermittent require documentation of onset and duration of each episode.

**Relationship to Malaria Challenge and to a Malaria Event**: Every effort will be made by the investigator to explain each adverse event and assess its causal relationship, if any, to administration of the malaria challenge or to a malaria event.

The degree of certainty with which an adverse event can be attributed to the malaria challenge (or alternative causes, e.g., concomitant medications, etc.) will be determined by how well the event can be understood in terms of one or more of the following:

- Reaction of similar nature having previously been observed with malaria challenge events.
- The incident having often been reported in literature from previous malaria challenge events.
- The incident being temporally associated with malaria challenge.

All AEs will have their relationship to the malaria challenge assessed using the terms: associated or not associated. All local (challenge-site) reactions extending past 48 hours will be considered causally related to the study unless alternate etiologies are determined.

- Associated – The event is temporally related to the administration of the malaria challenge and no other etiology explains the event.
- Not Associated – The event is temporally independent of the malaria challenge and/or the event appears to be explained by another etiology.

### Intensity of Response to the Malaria Challenge and to a Malaria Event

All AEs related to the malaria challenge event or to the smear-positive malaria event will be assessed by the clinician using the following protocol-defined grading system. The following guidelines will be used to quantify intensity. All AEs determined to be life-threatening will be categorized as an SAE.

Grade 0 = Absence of the indicated symptom

Grade 1 = Mild (awareness of a symptom but the symptom is easily tolerated)

Grade 2 = Moderate (discomfort enough to cause interference with usual activity)

Grade 3 = Severe (incapacitating; unable to perform usual activities; requires absenteeism or bed rest)

Grade 4 = Life-threatening event (requires hospitalization)

Solicited systemic AEs to the malaria challenge will include the following: body aches (exclusive of the injection site), urticaria, and headache from Days 2-7 (See Table 8). Other solicited AEs related to the challenge will include the following: Nausea, abdominal pain, diarrhea, fever, malaise, arthralgia and myalgia. These events will be categorized as related to the challenge event if the volunteer is documented as smear negative. Smear positive events will be termed solicited AEs to a malaria event (described below).

Solicited challenge site AEs will include the following: Persistent pain, erythema, and induration/swelling 48 hours or more after the malaria challenge event. (Note: challenge site AEs will be recorded for the first 48 hours but will not be deemed an AE unless persisting past this time frame.

Solicited AEs related to the challenge will include the following: Nausea, abdominal pain, diarrhea, fever, malaise, arthralgia and myalgia.

Solicited AEs to a malaria event will include the following: Malaise, chills/rigors, nausea, vomiting, abdominal pain, diarrhea, fever, dizziness, jaundice, arthralgia, myalgia and headache.

All AEs will be graded for severity and relationship to study product (i.e., malaria challenge with aseptic mosquitoes) as described in **Sections 9.2.2** and **9.2.3** in this protocol.

Changes in the severity of an AE will be documented as to the duration or until stabilization or resolution of the event with the maximum severity noted. Adverse events characterized as intermittent will be recorded both at the onset of the symptoms and conclusion of the complete event with each episode noted in narrative description of the event in the comments field for the AE. Each event will be followed for the entire duration of symptomatology. The intensity of the event will be assessed and graded by the investigator according to guidelines put forth in Tables 8 and 9.

| **Table 8. Challenge Solicited Adverse Event (AE) Assessment** | | | |
| --- | --- | --- | --- |
| **AE** | **Grade** | **Intensity Definition** | |
| Pain at challenge site | 0 | Absent | |
|  | 1 | Pain that is easily tolerated | |
|  | 2 | Pain that interferes with daily activity | |
|  | 3 | Pain that prevents daily activity | |
| Erythema at challenge site (> 48 hours post-challenge) | 0 | Absent | |
|  | 1 | 0-20 mm | |
|  | 2 | 21-50 mm | |
|  | 3 | > 50 mm | |
| Induration/swelling at challenge site (> 48 hours post-challenge) | 0 | Absent | |
|  | 1 | 0-20 mm | |
|  | 2 | 21-50 mm | |
|  | 3 | > 50 mm | |
| Malaise | 0 | Absent | |
|  | 1 | Malaise that is easily tolerated | |
|  | 2 | Malaise that interferes with daily activity | |
|  | 3 | Malaise that prevents daily activity | |
| Nausea | 0 | Absent | |
|  | 1 | Nausea that is easily tolerated | |
|  | 2 | Nausea that interferes with daily activity | |
|  | 3 | Nausea that prevents daily activity | |
| Abdominal Pain | 0 | Absent | |
|  | 1 | Abdominal pain that is easily tolerated | |
|  | 2 | Abdominal pain that interferes with daily activity | |
|  | 3 | Abdominal pain that prevents daily activity | |
| Diarrhea | 0 | Absent | |
|  | 1 | Diarrhea that is easily tolerated | |
|  | 2 | Diarrhea that interferes with daily activity | |
|  | 3 | Diarrhea that prevents daily activity | |
| Fever with negative malaria smear | 0 | < 37.5°C | < 99.5°F |
|  | 1 | 37.6-38.0°C | > 99.5 – 100.4°F |
|  | 2 | 38.1-39.0°C | > 100.4 – 102.2°F |
|  | 3 | > 39.0°C | > 102.2°F |
| Urticaria | 0 | None | |
|  | 1 | No medications required | |
|  | 2 | Topical or oral therapy required for < 24 hours | |
|  | 3 | Topical or oral therapy required for > 24 hours or iv medicine required | |
| Arthralgia with negative malaria smear | 0 | Absent | |
|  | 1 | Pain that is easily tolerated | |
|  | 2 | Pain that interferes with daily activity | |
|  | 3 | Pain that prevents daily activity | |
| Myalgia (Smear negative) | 0 | Absent | |
|  | 1 | Muscular pain that is easily tolerated | |
|  | 2 | Muscular pain that interferes with daily activity | |
|  | 3 | Muscular pain that prevents daily activity | |
| Headache (Smear negative) | 0 | Absent | |
|  | 1 | Headache that is easily tolerated | |
|  | 2 | Headache that interferes with daily activity | |
|  | 3 | Headache that prevents daily activity | |

| **Table 9. Malaria Solicited Adverse Event (AE) Assessment** | | | |
| --- | --- | --- | --- |
| **AE** | **Grade** | **Intensity Definition** | |
| Malaise | 0 | Absent | |
|  | 1 | Malaise that is easily tolerated | |
|  | 2 | Malaise that interferes with daily activity | |
|  | 3 | Malaise that prevents daily activity | |
| Chills/rigors | 0 | Absent | |
|  | 1 | Symptoms that are easily tolerated | |
|  | 2 | Symptoms that interfere with daily activity | |
|  | 3 | Symptoms that prevent daily activity | |
| Nausea | 0 | Absent | |
|  | 1 | Nausea that is easily tolerated | |
|  | 2 | Nausea that interferes with daily activity | |
|  | 3 | Nausea that prevents daily activity | |
| Vomiting | 0 | Absent | |
|  | 1 | Vomiting that is easily tolerated | |
|  | 2 | Vomiting that interferes with daily activity | |
|  | 3 | Vomiting that prevents daily activity | |
| Abdominal Pain | 0 | Absent | |
|  | 1 | Abdominal pain that is easily tolerated | |
|  | 2 | Abdominal pain that interferes with daily activity | |
|  | 3 | Abdominal pain that prevents daily activity | |
| Diarrhea | 0 | Absent | |
|  | 1 | Diarrhea that is easily tolerated | |
|  | 2 | Diarrhea that interferes with daily activity | |
|  | 3 | Diarrhea that prevents daily activity | |
| Fever | 0 | < 37.5°C | < 99.5°F |
|  | 1 | 37.6-38.0°C | > 99.5 – 100.4°F |
|  | 2 | 38.1-39.0°C | > 100.4 – 102.2°F |
|  | 3 | > 39.0°C | > 102.2°F |
| Dizziness | 0 | None | |
|  | 1 | Dizziness that is easily tolerated | |
|  | 2 | Dizziness that interferes with daily activity | |
|  | 3 | Dizziness that prevents daily activity | |
| Arthralgia | 0 | Absent | |
|  | 1 | Pain that is easily tolerated | |
|  | 2 | Pain that interferes with daily activity | |
|  | 3 | Pain that prevents daily activity | |
| Myalgia | 0 | Absent | |
|  | 1 | Muscular pain that is easily tolerated | |
|  | 2 | Muscular pain that interferes with daily activity | |
|  | 3 | Muscular pain that prevents daily activity | |
| Headache | 0 | Absent | |
|  | 1 | Headache that is easily tolerated | |
|  | 2 | Headache that interferes with daily activity | |
|  | 3 | Headache that prevents daily activity | |
| Chest pain/discomfort | 0 | Absent | |
|  | 1 | Chest pain that is easily tolerated | |
|  | 2 | Chest pain that interferes with daily activity | |
|  | 3 | Chest pain that prevents daily activity | |
| Shortness of breath | 0 | Absent | |
|  | 1 | Shortness of breath that is easily tolerated | |
|  | 2 | Shortness of breath that interferes with daily activity | |
|  | 3 | Shortness of breath that prevents daily activity | |
| Change in exercise tolerance | 0 | Absent | |
|  | 1 | Change that is easily tolerated | |
|  | 2 | Change that interferes with daily activity | |
|  | 3 | Change that prevents daily activity | |
| Palpitations | 0 | Absent | |
|  | 1 | Palpitations that are easily tolerated | |
|  | 2 | Palpitations that interfere with daily activity | |
|  | 3 | Palpitations that prevent daily activity | |

### Serious Adverse Events

An SAE is an AE, whether or not related to the malaria challenge or to the smear-positive malaria event that meets one of the following criteria. SAEs will be followed and reported throughout the 365 day follow-up surveillance period of the study.

1. *Death:* Defined as occurring within the period of protocol-defined surveillance
2. *Life threatening*: Defined as an event that places a participant at risk of death at the time of the event (this does not refer to any hypothetical outcomes that might have caused death/debilitation if it were more severe). Severe malaria episodes will be categorized under this criterion.
3. *Requires inpatient hospitalization or prolongation of existing hospitalization* Defined as an event requiring hospitalization other than the inpatient hospitalization already outlined as part of the protocol (Days 8-18).
4. *Results in congenital anomaly/birth defect*:
5. *Results in a persistent or significant disability or incapacity*: Event associated outcome resulting in debilitation.
6. *Other important medical event:* An event that may not result in death, be life threatening, or require hospitalization, may be considered an SAE when, based upon appropriate medical judgment, the event may jeopardize the subject and may require medical or surgical intervention to prevent one of the outcomes listed above. Examples of such medical events include anaphylaxis requiring intensive treatment in an emergency room or at home, seizure activity related to malaria, autoimmune phenomenon that do not result in inpatient hospitalization, or the development of drug dependency or substance abuse.

All SAEs will be:

- - recorded on the appropriate DMID-specific SAE form.
  - followed through resolution by a study clinician
  - reviewed and evaluated by a study clinician and the ISM

### Procedures to be Followed in the Event of Abnormal Laboratory Test Values or Abnormal Clinical Findings

Collection of laboratory data will be limited to those laboratory parameters that are relevant to safety, study outcome measures, and/or clinical outcome. LabCorp of Herndon, Virginia will be responsible for most testing results (with University of Maryland laboratories as a back-up). Troponin levels be performed at the University of Maryland. They will not be graded but will be evaluated in the context of elevated (> 0.1 ng/mL) versus not elevated (0-0.1 ng/mL). Laboratory test abnormalities will be analyzed based on the following grading scale:

**Table 10:** Laboratory testing ranges for LabCorp blood test results and grading scale.

| **Laboratory** | **Normals** | **Mild (Grade 1)** | **Moderate (Grade 2)** | **Severe (Grade 3)** | **Life-threatening (Grade 4)** |
| --- | --- | --- | --- | --- | --- |
| Hemoglobin (♀) – change from baseline value in gm/dL | 11.5-15.0 | Any decrease  2.4-3.4 from baseline | Any decrease 3.5-4.4 from baseline | Any decrease > 4.5 from baseline | < 7.0 g/dl |
| Hemoglobin (♂) – change from baseline value in gm/dL | 12.5-17.0 | Any decrease  2.4-3.4 from baseline | Any decrease 3.5-4.4 from baseline | Any decrease > 4.5 from baseline | < 7.0 g/dl |
| WBC – x 10^3^ cells/mm^3^  (Leukocytosis) | 3.5-10.5 | >10.5 & < 15.0 | ≥ 15 & < 20 | ≥ 20 | > 50 with fever (> 99.5°) |
| WBC – cells/mm^3^  (Leukopenia) |  | <3.5 & ≥ 2.5 | < 2.5 & ≥ 1.5 | <1.5 | <1.50 with fever (> 99.5°) |
| Platelets – x 10^3^ cell/mm^3^ | 140-415 | 125-139 | 100-124 | 20-99 | <20 |
| ASTs (increase by factor) IU/L | 0-40 | >1.0 & <2.5 x ULN | ≥ 2.5 & <5 x ULN | ≥ 5 x ULN* | >10 x ULN and requires hospitalization |
| ALTs (increase by factor) (♀) IU/L | 0-40 | >1.0 & <2.5 x ULN | ≥ 2.5 & <5 x ULN | ≥ 5x ULN* | >10 x ULN and requires hospitalization |
| ALTs (increase by factor) (♂) IU/L | 0-55 | >1.0 & <2.5 x ULN | ≥ 2.5 & <5 x ULN | ≥ 5x ULN* | >10 x ULN and requires hospitalization |
| Serum creatinine – mg/dL | 0.5-1.5 | >1.0 & <1.5 x ULN | ≥ 1.5 & <3 x ULN | ≥ 3 x ULN* | >5.0 x ULN and requires dialysis |
| Urine protein | 0 or 1+ | 2+ or 0.5-1 gm loss/day | 3+ or 1-2 gm loss/day | 4+ or >2 gm loss/day | NA |
| Hematuria | 0 or 1+ | 2+ confirmed by 5-10 rbc/hpf | 3+ confirmed by >10 rbc/hpf | gross, with or without clots, OR red blood cell casts | NA |
| Serum glucose – high mg/dL (fasting) | 65-110 | 111-140 | 141-250 | >251-500 | > 500 |
| Serum glucose – low mg/dL (fasting) | 65-110 | 55-64 | 40-54 | 30-39 | < 30 |

ULN is upper limit of normal

LLN is lower limit of normal

Adverse events and SAEs should be documented and reported appropriately. See **Sections 9.3 and 9.3.1.**

Urine pregnancy testing will be performed as specified in **Section 8.2.1** and pregnancy will be reported as specified in **Section 9.3.5**.

## Adverse Event Reporting Procedures

All deaths and life-threatening events, whether related or unrelated, will be reported by telephone, E-mail, or fax within 24 hours of site awareness. SAEs, other than death or life-threatening events, will be reported by telephone, E-mail or fax within 72 hours of site awareness (The University of Maryland IRB requires notification within 48 hours). In addition, Grade 3 AEs that meet the halting criteria for the study will be reported to the SMC (See **Section 9.4**). A written report will follow within 3 working days of the event. SAEs will be reported by the PI or co-investigator to the following:

- IND Sponsor, DMID: Protocol champion; Steven A. Rosenthal, MD, Division of Microbiology and Infectious Diseases, NIAID, NIH, Tel: 301-496-2544, e-mail: Srosenthal@niaid.nih.gov.
- Human Research Protections Office (University of Maryland IRB): Tel:(410) 706-5037, Fax: (410) 706-4189, email: HRPO@som.umaryland.edu.

### Serious Adverse Events

Any AE considered serious by the PI or Sub-investigator or that meets the aforementioned criteria must be submitted on an SAE form to PPD, Inc, NIAID’s pharmacovigilance contractor, at the following address:

**Medical Affairs/Pharmacovigilance
PPD, Inc
929 North Front Street
Wilmington, NC 28401-3331
SAE Fax line: 888-488-9697**

Questions about SAE reporting can be referred to the SAE Hotline (available 24 hours a day/7 days a week) at 800-201-8725.

The study clinician will complete a Serious Adverse Event Form within the following timelines:

- All deaths and immediately life-threatening events, whether related or unrelated, will be recorded on the Serious Adverse Event Form and sent by fax within 24 hours of site awareness.
- Serious adverse events other than death and immediately life-threatening events, regardless of relationship, will be reported via fax by the site within 72 hours of becoming aware of the event.

Other supporting documentation of the event may be requested by the pharmacovigilance contractor and should be provided as soon as possible.

All SAEs will be followed until satisfactory resolution or until the PI or Sub-Investigator deems the event to be chronic or the patient to be stable.

### Regulatory Reporting for Studies Conducted Under DMID‑Sponsored IND

Following notification from the investigator, DMID, the IND sponsor, will report events that are both serious and unexpected and that are associated with study product(s) to the Food and Drug Administration (FDA) within the required timelines as specified in 21 CFR Part 312.32: fatal and life-threatening events within 7 calendar days (by phone or fax) and all other SAEs in writing within 15 calendar days. All serious events designed as “not associated” to study product(s), will be reported to the FDA at least annually in a summary format.

### Regulatory Reporting for Studies Not Conducted Under DMID‑Sponsored IND

Not applicable

### Other Adverse Events (if applicable)

Not applicable

### Reporting of Pregnancy

All pregnancies that develop in participants within the 56 day window of the malaria challenge event (Day of challenge plus 55 days) will be reported by the Investigator to the Sponsor and IRB within 24 hours of learning of its occurrence. Pregnancy will be reported to DMID if it occurs within the 56 day window of the malaria challenge event and will be recorded into the database. The pregnancy should be followed every 12-16 weeks by telephone up until 30 days after delivery to determine outcome, including spontaneous or voluntary termination, details of the birth, and the presence or absence of any birth defects, congenital abnormalities, or maternal and/or newborn complications. It is anticipated that this window of follow-up would be encompassed within the original 365 day window of passive follow-up described in Table 4. All documentation of events during the pregnancy will be recorded on a Clinical Trial Pregnancy Form.

## Halting Rules

A total of 18 individuals randomized to one of three cohorts (1 bite* vs. 3 bites vs. 5 bites) will be enrolled in Part A of the malaria challenge study. *The 1 bite cohort will proceed if study recruitment numbers allow. The PI assisted by EMMES will identify when halting rule criteria are met and alert the appropriate parties. The SMC will review all available 56-day surveillance data subsequent to the malaria challenge of these individuals. The investigator will inform the UMD-IRB of relevant issues. All The SMC will be responsible for granting approval for proceeding to Malaria Challenge, Part B.

The following criteria will be used to define unacceptable intensity of response to either the mosquito challenge or to the subsequent clinical malaria event for both Parts A and Part B. Each will be examined in the context of the individual challenge event and not cumulative across both events. Furthermore, halting criteria will apply for 7 days after the challenge event (Table 8) but for the duration of the 56 study follow-up period in regards to the malaria episode (Table 9). It is important to note that a mild amount of local inflammation is expected as part of a normal human immunologic response in the first 48 hours after bite (see **Section 2.3.1.**). Additionally, an endpoint of this malaria challenge protocol will be the development of malaria infection and that the event itself is likely to trigger a variety of clinical symptoms such as fever, headache, malaise, and arthralgias/body aches.

1. One or more participants experience a SAE (as defined in **Section 9.2.3**) that is determined to be related to the study product administration (*Note: This includes a severe malaria episode as defined by World Health Organization^56^ criteria).
2. Two or more participants in a single group subset (i.e. Part A) experience an unsolicited Grade 3 or higher local allergic reaction characterized by ulceration, abscess, or necrosis associated with the malaria challenge.
3. Two or more participants in a single group subset (i.e. Part A) experience an unsolicited Grade 3 or higher systemic allergic reaction (i.e., bronchospasm, allergy-related edema/angioedema, hypotension or anaphylaxis) associated with the malaria challenge.
4. Three or more participants in a single group subset (i.e. Part A) report an unsolicited adverse event that is of Grade 3 or higher and is considered associated with the study challenge or other study procedures (this does not include clinical laboratory values that may have been reported as adverse events as these are captured in Halting Rules 6 & 7 and does not include allergic reactions as captured in Halting Rules 2 and 3).
5. Six or more participants (> 35%) in a single group subset (i.e. Part A) report the same solicited Grade 3 systemic AE and is considered associated with the study challenge or other study procedures. In the event that the 1 bite cohort is dropped, this will revert to 4 or more participants (> 35%) in a single group subset (Part A).
6. Two or more participants in a single group subset (i.e. Part A) experience a Grade 3 or higher laboratory abnormality in the absence of a positive malaria smear that is determined to be related to study product administration.
7. Three or more participants in a single group subset (i.e. Part A) experience a Grade 3 or higher laboratory abnormality in the presence of a positive malaria smear that is determined to be related to the malaria event.
8. Any clinical cardiac event that does not meet the criteria of SAE (as defined in Section 9.2.3)

If any of the stopping rules are invoked through the course of the Malaria Challenge, Part A, the study will not proceed to the next cohort or step in the protocol, Malaria Challenge, Part B without authorization and consultation with the SMC. The SMC members will elect an SMC chair who will assume responsibility for relaying committee decisions to DMID and the University of Maryland.

## Safety Oversight (ISM plus SMC)

### Independent Safety Monitor

A qualified physician will serve as the Independent Safety Monitor (ISM) for this study. He or she will be a qualified and experienced physician not otherwise associated with this protocol, who is able to provide medical care to research subjects for conditions that may arise during the conduct of the study and oversee the safety aspects of the study. The ISM will sit on the SMC. At a minimum, the ISM will comment on the outcomes of the serious adverse event (SAE) and relationship of the SAE to the test product. The ISM will also indicate whether s/he concurs with the details of the report provided by the study investigator.

The ISM will be available during active phases of malaria challenge and during the immediate post-challenge surveillance period.

The ISM’s role will include:

- Acting as a member of the SMC
- As a member of the SMC, reviewing accumulated safety data and recommending proceeding with the subsequent malaria challenge
- Providing advice to the investigators on whether a set of clinical circumstances in a study warrants formal notification to the sponsor and the SMC.
- Providing clinical advice on any illness in study subjects especially in circumstances in which treatment might influence the course of the trial.
- Review all SAEs as outlined above

The ISM will liaise closely with DMID throughout the course of the trial and relay relevant safety information to the PI. The PI will subsequently inform the IND sponsor (DMID), and the UMD IRB of safety concerns that arise.

### Safety Monitoring Committee

An independent Safety Monitoring Committee (SMC) will be constituted to review safety data at specified intervals prior to the next malaria challenge (Part B). The membership of the SMC will include expertise in clinical vaccine trials and clinical malaria. The role of the SMC is to provide safety oversight over the conduct of the trial. It will review safety data accumulated in Part A of the malaria challenge study and advise on progression to Part B. The SMC will hold conference calls to review the 56-day safety data generated from the trial and advise whether or not the study can proceed to Part B. Senior investigators and representatives from DMID and Sanaria, Inc, will be invited but not obligated to participate in the conference calls during open sessions, which may be followed by closed sessions including only SMC members.

DMID will inform the SMC of:

- All subsequent protocol amendments, informed screening or study consent form changes or revisions of other documents originally submitted for review
- Serious adverse events (SAEs)
- Adverse events that meet the halting criteria (Section 9.4)
- New information that may affect adversely the safety of the subjects or the conduct of the study.

See **Section 11.4.1** for schedule of SMC meetings in relation to the study timeline.

# Clinical Monitoring

## Site Monitoring Plan

Site monitoring will be conducted to ensure that human subject protection, study procedures, laboratory procedures, study intervention administration, and data collection processes are of high quality and meet sponsor, GCP/ICH, and regulatory guidelines, and that the study is conducted in accordance with the protocol and sponsor standard operating procedures. DMID, the sponsoring agency, or its designee will conduct site-monitoring visits as detailed in the monitoring plan or in the Manual of Procedures.

Site visits will be made at standard intervals as defined by DMID and may be made more frequently as directed by DMID. Monitoring visits will include, but are not limited to, review of regulatory files, accountability records, CRFs, informed consent forms, medical and laboratory reports, and protocol compliance. Study monitors will meet with investigators to discuss any problems and actions to be taken and document visit findings and discussions.

# Statistical Considerations

This study will establish whether aseptic anopheles mosquitoes are capable of developing salivary gland infections by the most widely used *P. falciparum* challenge isolate, NF54, and whether these infections can be transmitted to human volunteers. It is largely a descriptive and “proof of concept” study and as such, only initial point estimates will be calculated without formal hypothesis testing. We seek to identify an optimal, aseptically-raised *A. stephensi* mosquito bite quantity to transmit subclinical falciparum malaria strain NF54 parasitemia to 100% (ID_100_) of malaria-naïve, U.S. volunteers for use in challenge models designed to test the efficacy of malaria vaccine constructs and drugs. Additionally, we will examine the prepatent period durations between bite cohorts to measure time to malaria onset as a function of sporozoite load. This study will explore the use of aseptically-raised mosquitoes, refine the methodology previously established by personnel at the CVD and improve the design of malaria challenge models. These applications are critical to improving the methodology of malaria challenge studies and for standardization of the challenge model to all vaccine constructs and drugs.

## Overview of Study Hypotheses

**Primary Objectives:**

1. Develop and evaluate the safety and reactogenicity of a new human malaria challenge model using aseptically-raised anopheles mosquitoes infected with the NF54 isolate of *P. falciparum* and reared under conditions similar to current Good Manufacturing Practice (cGMP)

**Secondary objectives:**

1. Obtain information on the minimum number of *A. stephensi* bites required to safely achieve 100% adult human volunteer infectivity (Malaria challenge, Part A).
2. Obtain information on the minimum quantity of bites in a second challenge study to achieve 100% adult human volunteer infectivity (Malaria challenge, Part B).
3. Develop molecular diagnostic techniques for rapid and accurate real-time diagnosis of *P. falciparum* infection, to assess a role as a new diagnostic standard for *P. falciparum* challenge studies.

**Primary Outcome Measures:**

- Occurrence of a positive malaria smear after the malaria challenge during a 56-day surveillance period (day of challenge and days 5-28, 35, 42, 49, and 56 after challenge)
- Occurrence and severity of solicited symptoms for 7 days after the malaria challenge or for the duration of follow-up after a malaria event (treatment days x 3 and smear-negative x 2 plus weekly follow-up x 4).
- Occurrence and severity of unsolicited symptoms after the malaria challenge during a 56-day surveillance period (day of challenge and 55 subsequent days. Volunteers will be questioned at Visits 2-30).
- Occurrence of serious adverse events throughout the study period

**Secondary Outcome Measures:**

- Occurrence of a positive real-time quantitative PCR after the malaria challenge during a 56-day surveillance period (day of challenge and days 5-28, 35, 42, 49, and 56 after malaria challenge).

## Sample Size Considerations

This trial is not powered to detect differences between groups. Even if comparative statistics for the safety variables are computed, the study will have low power to detect anything other than very large differences in the incidence of local and systemic side effects between the challenge groups. This is done weighing the need to detect any possible untoward reactions against the need to limit the number of volunteers involved for safety purposes. The sample size of 6 in the 1*, 3 and 5 bite cohorts (Malaria Challenge, Part A) and 20 in Malaria Challenge, Part B is felt to be necessary and sufficient for the initial assessment of the safety, tolerance and transmission efficiency of the study product, *Pf* infected aseptic anopheles mosquitoes. This sample size takes into account an estimated 20% loss to follow-up of which the sample size will be 5 in the 1*, 3 and 5 bite groups of Part A and 16 in Part B. Two additional volunteers will be screened in preparation of challenge in the event that individuals do not appear at the scheduled time for challenge. These volunteers can be included in the Part B cohort of volunteers in the event that their services are not required for Part A. A contingency plan has been developed in the event of reduced recruitment numbers. If the necessary 18 volunteers are not enrolled, the 1 bite cohort will be dropped in favour of randomization between the 3 and 5 bite cohorts. This will require at least 12 volunteers to be enrolled. The statistical power will not change as the 1 bite cohort was added to establish the lower bound of efficiency of transmission of malaria but was never intended to operate as a legitimate and reliable bite quantity for a malaria challenge model. The statistics generated rely upon the sample size of the specific cohort ID_100_. (See **Section** 11.3)

## Statistical Considerations

These are largely descriptive and proof of concept studies. Statistical consideration has, however, been given to the proposed studies. Each subject’s exposure to the randomized number of bites (1*, 3, or 5) will be considered a separate Bernoulli trial with a ‘success’ defined as a positive malaria smear prior within 56 days of exposure. The results for each of the three cohorts in Part A will be reported as a point estimate and 95% exact-binomial confidence interval for the probability of successful transmission. If all 6 volunteers of a single cohort become infected, the 95% confidence interval will be [0.54, 1.0]. All 6 volunteers of a single cohort (e.g. All members of the 5 bite cohort) must become infected for consideration of progressing to Part B. The number of bites to be assessed in Part B will depend upon the minimum number of bites required to confer 100% infectivity (1*, 3 or 5 bites). Twenty volunteers will be enrolled for Part B. If all 20 become infected, the 95% CI will be [0.83, 1.0], with a maximum half-width of 0.23 if the incidence is less than 100%. Combining cohorts (6 plus 20 for a total of 26 volunteers) will provide a maximum 95% confidence interval half-width of approximately 0.2. Allowing for a loss to follow-up of 20%, and if 5 subjects in a cohort are ‘successes’ we will have a 95% CI of [0.48, 1.0] for a single cohort (n = 5), and maximum half-widths of 0.25 (n=16) and 0.23 (n=21) respectively for Part B and Parts A and B combined. *The 1 bite cohort will proceed if study recruitment numbers allow.

Confidence intervals for incidence of SAEs, severe adverse events or reactogenicities will have half-widths as the same as described above for cohorts in Part A, Part B or the combination. In a given cohort in Part A (n=6), a 95% confidence interval for unobserved adverse events or reactogenicities will not exclude events with an incidence rate as high as 0.46. Similarly, in Part B (n=20), unobserved events with a true incidence rate as high as 0.16 will not be ruled out. In combining a cohort from Part A and results from Part B (n=26), unobserved events with a true incidence with a true incidence rate of up to 0.13 will not be ruled out.

The median time to infection (pre-patent period duration) (i.e.: study-day of the malaria-positive smear) will be calculated. In part B confidence intervals for this time will be estimated using either an inversion of a sign-test, or the variance-stabilized method of Brookmeyer and Crowley, depending on the distribution of the infection times (Brookmeyer R, Crowley J. A confidence interval for the median survival time. *Biometrics* 1982;38:29-41).

Additionally, some preliminary assessment of the agreement of real-time PCR to the smear analysis will be conducted and presented using a standard Kappa statistic (adjusted for chance). McNemar’s test for equivalent distribution of concordance will also be used to investigate the false-positive/negative rates of the real-time PCR technique. These analysis will be conducted on matched, paired smear and RTQ-PCR test results as sets of independent tests.

## Planned Interim Analyses

This malaria challenge study is sub-divided into two sequential studies, Malaria Challenge, Part A and Part B. The results of Part A will inform the specific design of Part B. Interim 56-day safety analyses will be reviewed by the SMC. Inducing a malaria episode via the infected bite of female, aseptic anopheles mosquitoes is the primary goal of this study. Having stated this, providing for the safety of the human volunteers is paramount. Our group and others have used the *P. falciparum* challenge isolate, NF54, in prior studies without complication. This particular strain is highly sensitive to chloroquine sulfate base and to other licensed antimalarials. No drug resistance has been documented with this challenge isolate. Interim analysis will provide a safety checkpoint in which to review all data from Part A and determine if it is advisable to commence with Part B (See **Section 11.5.1** **Safety Review** below for more details).

### Safety Review

Refer to **Table 4** for the tabular estimate of the safety review timeline. One of the primary safety endpoints of this study is the occurrence of unsolicited symptoms/adverse events after the malaria challenge during a 56-day surveillance period (day of challenge and 55 subsequent days). Local and systemic reactions will be analyzed. The incidence, intensity and relationship of individual solicited symptoms over the 56-day surveillance period will be calculated for both Part A and Part B. The SMC will review all available 56-day safety data on ~Day 60 (Day 56-91) of Part A. Based on this review (as outlined in **Section 11.4**), the SMC will provide a recommendation as to whether it is safe and appropriate to proceed to Part B in an effort to refine the malaria challenge model. Malaria challenge Part B will commence no sooner than 7 days after SMC approval and will enroll 20 individuals for malaria challenge B (and may be split into two groups of 10 individuals, if performed at the GCRC, due to bed limitations). The SMC will meet within five weeks after completion of malaria challenge B ~Day 60 (Day 56-91) safety data (note: ~Day 60 of group 2 if performed at the GCRC). Residual data from Part A can be presented at this time if pertinent. The SMC will refer to **Section 9.4** as a guideline for stopping criteria. In addition to the scheduled SMC meetings described, the study team may ask for an ad hoc SMC meeting if study results warrant.

### Efficacy Review

Efficacy analysis in this study refers to the efficiency at which aseptic mosquitoes are able to transmit malaria sporozoites into the human volunteers via a bite. While prior studies performed at the University of Maryland and other institutions have developed a model in which 5 bites are necessary and sufficient to induce malaria in 100% of volunteers, this model is performed utilizing mosquitoes that have been raised in the traditional non-sterile environment of the insectary. As such, it is the goal of this study to determine whether aseptic mosquitoes are as effective, less effective or ineffective at transmitting malaria to the human host. Furthermore, close attention will be paid to the prepatent period duration between cohorts. The results of Part A will dictate the number of mosquito bites will be tested as a means of refining the number required for 100% infection. Thus, if all volunteers in the 3 bite cohort develop malaria but all volunteers in the 1 bite cohort do not, the malaria challenge design of Part B will involve testing 20 volunteers with 3 bites and determining whether this new bite threshold meets the requirement of an ID_100_. Concerns remain as to the efficacy of a 1 bite model being able to reliably confirm protective efficacy of a malaria vaccine. For the purposes of establishing a lower bound for the number of mosquito bites required for an ID_100_, we will test the one bite challenge model (if study recruitment numbers allow). In reality, we do not intend on reducing the number of mosquito bites to below 3 for future aseptic challenge protocols as the ramifications of suffering an error using a one bite challenge model are too great (i.e., falsely assuming vaccine protective efficacy exists due to an improperly administered challenge event).

## Final Analysis Plan

The interim analyses will be conducted on Day 56 data from Malaria Challenge, Part A. Based on these results, a confirmation as to the ID_100_ of the malaria challenge model can done with a larger cohort of volunteers (Part B, n = 20). Similarly, final analyses will be conducted on Day 56 data from Malaria Challenge, Part B. The study will continue in passive follow-up for additional safety surveillance. Other than the estimation of malaria infection rates, event incidence rates and time to positive smear described above, data analysis will be primarily descriptive in nature. No formal hypothesis testing or comparisons between groups will be performed. Adverse events, reactogenicity events, and any other clinical findings will be presented by group.

It is anticipated that the results of this study will be presented to the scientific community via oral presentations at meetings and written publications in scientific journals. The data to be presented and the authorship will be discussed between investigators and sponsors, and approved by the sponsors, prior to any official communication.

The official report of the primary analysis will be submitted through appropriate channels and after review by DMID. This report will contain detailed information about the participants, their tolerance of the malaria challenge, their side effects and laboratory abnormalities, as well as their overall response to malaria challenge.

# Source Documents and Access to Source Data/Documents

In compliance with ICH E6, Section 4.9, complete source documentation (which include but are not limited to outpatient and inpatient records, laboratory test reports, malaria smears and PCR results) will be maintained on every subject throughout the duration of the study. Source documents derived from the electronic CRFs will be the central record for recording data on volunteers enrolled in the study. All clinical surveillance reports will be recorded into the CRF and results from source documentation will be recorded into the electronic CRF. The accuracy of this transfer of data will be the responsibility of the investigator. Only authorized study personnel will have direct access to these documents and the management of these documents will be the joint responsibility of the principal investigator and nurse study coordinator. As part of participating in a DMID-sponsored, DMID-affiliated, or manufacturer-sponsored study, authorized representatives of the sponsor(s), DMID, and regulatory agencies will be allowed to examine (and when required by applicable law, to copy) clinical records for the purposes of quality assurance reviews, audits, and evaluation of the study safety and progress.

# Quality Control and Quality Assurance

SOPs for quality management will be developed, used to train appropriate personnel, and kept on file with documentation of training. Data will be evaluated for compliance with protocol and accuracy in relation to source documents. The study will be conducted in accordance with procedures identified in the protocol. The types of materials to be reviewed, the personnel responsible, and the schedule for reviews will be referenced in the SOPs. Study-specific training will be provided for all staff prior to the commencement of the trial.

The clinical portion of the study will be conducted at a single center, the Center for Vaccine Development at the University of Maryland medical campus. All challenge mosquitoes and parasites will be reared and cultured at the Sanaria, Inc. facilities in Rockville, MD and will be transported to the University of Maryland within 24 hours of malaria challenge. All laboratories will be performed at the University of Maryland with the exception of a small aliquot assigned for research purposes (See **Section 8.2.2**).

SOPs will be used at all clinical and laboratory sites. Regular monitoring and an independent audit will be performed according to GCP/ICH (e.g., data monitoring). Following written standard operating procedures, the monitors will verify that the clinical trial is conducted and data are generated, documented (recorded), and reported in compliance with the protocol, GCP, and the applicable regulatory requirements. Reports will be submitted to DMID on monitoring activities.

The investigational site will provide direct access to all trial related sites, source data/documents, and reports for the purpose of monitoring and auditing by the sponsor, and inspection by local and regulatory authorities.

The Data Coordinating Center, EMMES, will implement quality control procedures beginning with the data entry system and generate data quality control checks that will be run on the database. Any missing data or data anomalies will be communicated to the site(s) for clarification/resolution.

# Ethics/Protection of Human Subjects

## Ethical Standard

The investigator will ensure that this study is conducted in full conformity with the principles set forth in The Belmont Report: Ethical Principles and Guidelines for the Protection of Human Subjects of Research of the US National Commission for the Protection of Human Subjects of Biomedical and Behavioral Research (April 18, 1979) and codified in 45 CFR Part 46 and/or the ICH E6; 62 Federal Regulations 25691 (1997).

## Institutional Review Board

Before commencing with and enrolling volunteers for this proposed study, the appropriate documents (including the Protocol, Investigator’s Brochure, Informed Consent Form, information sheets, CRF’s and advertisements) will be submitted to the IRB. The Principal Investigator will be responsible for obtaining IRB approval for the study. Protocol deviations will also be reported to the IRB according to the policy of the board. The investigators will inform all the IRBs and DMID of the following:

- All subsequent protocol amendments, informed consent changes or revisions of other documents originally submitted for review
- Serious and/or unexpected adverse events occurring during the study, where required
- New information that may affect adversely the safety of the participants or the conduct of the study
- An annual update and/or request for re-approval, where required (barring unforeseen circumstances, this study is anticipated to be completed within 10 months although passive follow-up continues for 12 months after malaria challenge with a 6 and 12 month telephone follow-up proposed).
- When the study has been completed.

## Informed Consent Process

The investigator will choose participants in accordance with the eligibility criteria put forth in **Section 5.1** and **5.2** without introducing selectivity or bias to the process. Extensive discussion of risks and possible benefits of this therapy will be provided to the subjects. Consent forms describing in detail the study interventions/products, study procedures, and risks are given to the subject and written documentation of informed consent is required prior to starting intervention/administering study product. Consent forms will be IRB-approved and the subject will be asked to read and review the document. Upon reviewing the document, the investigator and/or designee will explain the research study to the subject and answer any questions that may arise. The subjects will be given sufficient time to discuss the study with their surrogates or think about it prior to agreeing to participate. The subjects will sign the informed consent document prior to any procedures being done specifically for the study. The subjects should have the opportunity to discuss the study with their surrogates or think about it prior to agreeing to participate. The subjects may withdraw consent at any time throughout the course of the trial. A copy of the informed consent document will be given to the subjects for their records. The Investigator will comply with all applicable regulatory requirements, Good Clinical Practices, HIPAA and ethical principles. If the study participants choose to voluntarily withdraw from the study, close follow-up will be provided regardless of study participation in the event that the individuals have received a malaria challenge that could put them at risk for the development of malaria. Given the importance of participant safety, participants who choose to withdraw (prior to developing a malaria episode) will be contacted daily. They will be offered Malarone® malaria treatment doses to prevent erythrocytic-stage *P. falciparum* parasite development. Treatment doses are as follows: 4 tablets (250/100 mg) orally for three days, representing a total daily treatment dose of 1 gram/400 mg. Attempts will be made to convince them to return to the CVD for daily malaria smears. If they refuse, daily contact with maintenance of solicited and unsolicited symptoms will be recorded. If a volunteer refuses therapy and symptoms of malaria develop, he/she will be asked to notify study personnel immediately and treatment will be offered through the University of Maryland medical system. In the event that a volunteer withdraws after experiencing a malaria episode, outpatient follow-up in the form of telephone contact will occur weekly x 4 weeks as is proposed for ongoing study participation.

### Informed Consent/Assent Process (in Case of a Minor)

Not applicable

## Exclusion of Women, Minorities, and Children (Special Populations)

All healthy adults between the ages of 18 to 40 years of age, who meet the inclusion/exclusion criteria, regardless of religion, sex, or ethnic background, will be included in the study. Due to the nature of the study, and the fact that no benefit exists to the U.S. malaria-naïve volunteer, children will not be enrolled at this time.

## Subject Confidentiality

Subject confidentiality is held strictly in trust by the participating investigators, their staff, and the sponsor(s) and their agents. This confidentiality is extended to cover testing of biological samples and genetic tests (i.e., microarray analysis) in addition to the clinical information relating to participating subjects. Participants will be assigned a unique study number. All results will be keyed to this number. No information concerning the study or the data will be released to any unauthorized third party without prior written approval of the sponsor.

Study records will only be available to staff members and will be kept locked at the study site conforming to the investigators’ SOPs. All computer entry will be done by coded number only, and all local databases will be secured with password-protected access systems. The study monitor or other authorized representatives of the sponsor may inspect all documents and records required to be maintained by the investigator, including but not limited to, medical records (office, clinic, or hospital) and pharmacy records for the subjects in this study. The clinical study site will permit access to such records.

## Study Discontinuation

In the event that the study is discontinued prior to completion, all volunteers who received a malaria challenge and did not develop clinical symptoms of malaria, will be offered Malarone® malaria treatment doses to prevent erythrocytic-stage *P. falciparum* parasite development. Treatment doses are as follows: 4 tablets (250/100 mg) orally for three days, representing a total daily treatment dose of 1 gram/400 mg. The participants will be asked to follow up weekly with the study team until Surveillance Day 56 to assure that malaria therapy is taken reliably and that malaria does not develop. In the event that the study volunteer has developed malaria with eradication of the parasite after protocol-specified treatment with chloroquine or Malarone®, the participant will be asked to follow up with the study team at the Day 56 surveillance follow-up for debriefing. In the event that the study volunteer refuses drug treatment, attempts will be made to convince them to return to the CVD for daily malaria smears. If they refuse, daily contact with maintenance of solicited and unsolicited symptoms will be recorded. If a volunteer refuses therapy and symptoms of malaria develop, he/she will be asked to notify study personnel immediately and treatment will be offered through the University of Maryland medical system. Study team personnel will be available for questions or follow-up should evaluation be needed prior to Surveillance Day 56. Study team personnel will also be available for a time period of 365 days for passive follow-up should symptoms arise relating to malaria. Participants will be telephoned at 6 and 12 months.

## Future Use of Stored Specimens

If residual sera and PBMC are available following the assays described in this protocol, additional immunological or microbiological assays may be performed on those samples for which permission was expressly granted for preserving samples for future studies at the time of informed consent at study enrollment. Samples from participants who did not grant permission to preserve samples will be discarded after the analyses described in this protocol have been completed.

## Compensation

Subjects will be compensated for the participation in this study. Compensation will be in accordance with the local IRB’s policies and procedures, and subject to IRB approval. Below is a scale of compensation with an example of reimbursement totals for a 3 day and 11 day inpatient stay in a participant who acquires malaria.

**Table 10:** Compensation schedule

| **Payment schedule:** | Screening | Challenge | Diary | Outpt Visit | Inpatient Treatment Days | Partial Inpatient Days | Bonus |
| --- | --- | --- | --- | --- | --- | --- | --- |
| Cost | $50 | $250 | $50/week | $50 | $150 | $75 | $250 |
| Unit Quantity | 1 | 1 | 1 | 8-18 | 0-3 | 0-11 | 1 |
|  |  |  |  |  |  |  |  |
| sub-totals (max) | $50 | $250 | $50 | $900 | $450 | $825 | $250 |
|  |  |  |  |  |  |  |  |
| Total (11 Inpatient Days)* = |  |  | $2,050 |  |  |  |  |
| Total (3 Inpatient Days)** = |  |  | $1,450 |  |  |  |  |
| Total (malaria not transmitted) = |  |  | $2,325 |  |  |  |  |

*In rare instances, it is conceivable that a participant may require more than 11 inpatient days (e.g., if the participant were to develop smear positivity on Day 18 and required two additional days of observation/therapy and/or additional days until documentation of smear negativity x 2). This should be an uncommon event given that the mean time to presentation with smear positivity after challenge was 10.5 days (range 9-14) and the maximum time to presentation was 20 days in a single individual (out of 527 evaluated).^18^

**We have depicted a typical reimbursement schedule for a three day inpatient stay as it represents the projected minimum reimbursement for a participant who acquires a malaria episode (at the earliest possible time). This assumes that the volunteer acquires malaria on Day 8 and is cleared of malaria within 48 hours.

# Data Handling and Record Keeping

Data-handling, record-keeping, reporting, study record retention and protocol deviations will be managed in accordance with GCP and DMID guidelines will be described in detail in study-specific SOPs.

The investigator is responsible for ensuring the accuracy, completeness, legibility, and timeliness of the data reported. All source documents will be legible to ensure accurate interpretation of data. Black ink is required to ensure clarity of reproduced copies. Corrections will be addressed by crossing out the original entry with a single line, initializing and dating the change. No erasure, overwriting or use of correction fluid or tape on the original document will be permitted.

Source documents derived from the electronic CRF (eCRF) will be provided and maintained for recording data for each subject enrolled in the study. Data reported in the eCRF derived from source documents will be consistent with the source documents. All discrepancies will be evaluated and explained.

The sponsor, DMID, and/or it designee (EMMES) will provide guidance to investigators on making corrections to the source documents and eCRF.

## Data Management Responsibilities

All source documents and laboratory reports will be reviewed by the clinical team and data entry staff, who will ensure that they are accurate and complete. Adverse events must be graded, assessed for severity and causality, and reviewed by the site PI or designee.

Data collection will be the responsibility of the clinical trial staff at the site under the supervision of the site principal investigator. During the study, the investigator must maintain complete and accurate documentation for the study.

The EMMES Corporation will serve as the Statistical and Data Coordinating Center for this study, and will be responsible for data management, quality review, analysis, and reporting of the study data.

## Data Capture Methods

Clinical data (including AEs, concomitant medications, and solicited symptoms to the malaria challenge event) and clinical laboratory data will be entered into a 21 CFR Part 11-compliant Internet Data Entry System (IDES). The data system includes password protection and internal quality checks, such as automatic range checks, to identify data that appear inconsistent, incomplete, or inaccurate. Clinical data will be entered directly from the source documents.

## Types of Data

Data for this study will include safety (clinical signs and symptoms and clinical laboratory tests), laboratory (immunologic), and outcome measures (e.g., reactogenicity and immunogenicity).

## Timing/Reports

Safety data for Malaria Challenge A will be reviewed by the SMC after 56 days of surveillance. The SMC will make a recommendation at that time as to the advisability of proceeding to the next challenge cohort (or to Malaria Challenge B after all cohorts have been challenged and it is deemed to be safe to proceed to the second stage of the study). Interim safety reports will be generated after all of the Day 56 safety and outcome data have been analyzed.

## Study Records Retention

Study documents should be retained for a minimum of 2 years after the last approval of a marketing application in an ICH region and until there are no pending or contemplated marketing applications in an ICH region or at least 2 years have elapsed since the formal discontinuation of clinical development of the investigational product. These documents should be retained for a longer period, however, if required by local regulations. No records will be destroyed without the written consent of the sponsor, if applicable. It is the responsibility of the sponsor to inform the investigator when these documents no longer need to be retained.

## Protocol Deviations

A protocol deviation is any noncompliance with the clinical trial protocol, Good Clinical Practice (GCP), or Manual of Procedures requirements. The noncompliance may be either on the part of the subject, the investigator, or the study site staff. As a result of deviations, corrective actions are to be developed by the site and implemented promptly.

These practices are consistent with ICH E6:

4.5 Compliance with Protocol, sections 4.5.1, 4.5.2, and 4.5.3

5.1 Quality Assurance and Quality Control, section 5.1.1

5.20 Noncompliance, sections 5.20.1, and 5.20.2.

It is the responsibility of the site to use continuous vigilance to identify and report deviations within 5 working days of identification of the protocol deviation, or within 5 working days of the scheduled protocol-required activity. If a protocol deviation occurs, it must be reported to DMID, via entry in AdvantageEDC, within 5 days of site awareness and to the site's IRB in accordance with IRB's policy.

All deviations from the protocol must be addressed in study subject source documents. A completed copy of the IDES form will be maintained in the regulatory file, as well as in the subject’s source document. IDES will automatically notify DMID of the reported protocol deviation. Protocol deviations will be sent to the study site IRB/IEC on an annual basis. The site PI/study staff is responsible for knowing and adhering to their IRB/IEC requirements.

# Publication Policy

Following completion of the study, the investigator is expected to publish the results of this research in a scientific journal. The International Committee of Medical Journal Editors (ICMJE) member journals have adopted a trials-registration policy as a condition for publication. This policy requires that all clinical trials be registered in a public trials registry such as [ClinicalTrials.gov](http://www.clinicaltrials.gov/)*, which is sponsored by the National Library of Medicine. Other biomedical journals are considering adopting similar policies. It is the responsibility of DMID to register this trial in an acceptable registry. Any clinical trial starting enrollment after 01 July 2005 must be registered on or before patient enrollment. For trials that began enrollment prior to this date, the ICMJE member journals will require registration by 13 September 2005, before considering the results of the trial for publication.

The ICMJE defines a clinical trial as any research project that prospectively assigns human subjects to intervention or comparison groups to study the cause-and-effect relationship between a medical intervention and a health outcome. Studies designed for other purposes, such as to study pharmacokinetics or major toxicity (e.g., Phase I trials), would be exempt from this policy.

**Literature References**

1. Migliani R, Josse R, Hovette P et al. [Malaria in military personnel: the case of the Ivory Coast in 2002-2003]. Med Trop (Mars ) 2003; 63(3):282-286.

2. Tuck J, Green A, Roberts K. Falciparum malaria: an outbreak in a military population on an operational deployment. Mil Med 2003; 168(8):639-642.

3. World Bank. Health. World Bank Report . 1993. World Bank, Geneva.
Ref Type: Report

4. Breman JG. The ears of the hippopotamus: manifestations, determinants, and estimates of the malaria burden. Am J Trop Med Hyg 2001; 64(1-2 Suppl):1-11.

5. Boyd MF, Kitchen SF. Observations on induced falciparum malaria. Am J Trop Med Hyg 1937; 17:213-235.

6. James SP, Nicol WD, Shute PG. A study of induced malignant tertian malaria. Proc Natl Acad Sci U S A 1932; 25:1153-1181.

7. Church LW, Le TP, Bryan JP et al. Clinical manifestations of Plasmodium falciparum malaria experimentally induced by mosquito challenge. J Infect Dis 1997; 175(4):915-920.

8. Hoffman SL, Goh LM, Luke TC et al. Protection of humans against malaria by immunization with radiation-attenuated Plasmodium falciparum sporozoites. J Infect Dis 2002; 185(8):1155-1164.

9. Coggeshall LT, Maier J, Best CA. The effectiveness of two new types of chemotherapeutic agents in malari. JAMA 1941; 117:1077-1081.

10. Fairley NH. Sidelights on malaria in man obtained by subinoculation experiments. Trans R Soc Trop Med Hyg 1947; 40:621-676.

11. Cooper WC, Coatney GR. Studies in human malaria iii. the therapeutic effect of a phenanthrene amino alcohol, nih-204 (sn-1796), in vivax, falciparum, and quartan malaria. Am J Hyg 1947; 46:119-130.

12. MILLER MJ. Suppression of malaria by monthly drug administration. Am J Trop Med Hyg 1955; 4(5):790-799.

13. DAVEY DG, ROBERTSON GI. Experiments with antimalarial drugs in man. IV. An experiment to investigate the prophylactic value of proguanil against a strain of Plasmodium falciparum known to be resistant to therapeutic treatment. Trans R Soc Trop Med Hyg 1957; 51(5):463-466.

14. JEFFERY GM. Infectivity to mosquitoes of Plasmodium vivax following treatment with chloroquine and other antimalarials. Am J Trop Med Hyg 1958; 7(2):207-211.

15. JEFFERY GM, YOUNG MD, BURGESS RW, EYLES DE. Early activity in sporozoite-induced Plasmodium falciparum infections. Ann Trop Med Parasitol 1959; 53(1):51-58.

16. Laing AB. Antimalarial effects of sulphormethoxine, diaphenylsulphone and separate combinations of these with pyrimethamine: a review of preliminary investigations carried out in Tanzania. J Trop Med Hyg 1968; 71(2):27-35.

17. Powell RD, McNamara JV. Infection with chloroquine-resistant Plasmodium falciparum in man: prepatent periods, incubation periods, and relationships between parasitemia and the onset of fever in nonimmune persons. Ann N Y Acad Sci 1970; 174(2):1027-1041.

18. Epstein JE, Rao S, Williams F et al. Safety and clinical outcome of experimental challenge of human volunteers with Plasmodium falciparum-infected mosquitoes: an update. J Infect Dis 2007; 196(1):145-154.

19. Chulay JD, Schneider I, Cosgriff TM et al. Malaria transmitted to humans by mosquitoes infected from cultured Plasmodium falciparum. Am J Trop Med Hyg 1986; 35(1):66-68.

20. Herrington DA, Clyde DF, Losonsky G et al. Safety and immunogenicity in man of a synthetic peptide malaria vaccine against Plasmodium falciparum sporozoites. Nature 1987; 328(6127):257-259.

21. Ballou WR, Hoffman SL, Sherwood JA et al. Safety and efficacy of a recombinant DNA Plasmodium falciparum sporozoite vaccine. Lancet 1987; 329(8545):1277-1281.

22. Herrington DA, Clyde DF, Murphy JR et al. A model for Plasmodium falciparum sporozoite challenge and very early therapy of parasitaemia for efficacy studies of sporozoite vaccines. Trop Geogr Med 1988; 40(2):124-127.

23. Herrington D, Davis J, Nardin E et al. Successful immunization of humans with irradiated malaria sporozoites: humoral and cellular responses of the protected individuals. Am J Trop Med Hyg 1991; 45(5):539-547.

24. Edelman R, Hoffman SL, Davis JR et al. Long-term persistence of sterile immunity in a volunteer immunized with X-irradiated Plasmodium falciparum sporozoites. J Infect Dis 1993; 168(4):1066-1070.

25. Kester KE, McKinney DA, Tornieporth N et al. Efficacy of recombinant circumsporozoite protein vaccine regimens against experimental Plasmodium falciparum malaria. J Infect Dis 2001; 183(4):640-647.

26. McConkey SJ, Reece WH, Moorthy VS et al. Enhanced T-cell immunogenicity of plasmid DNA vaccines boosted by recombinant modified vaccinia virus Ankara in humans. Nat Med 2003; 9(6):729-735.

27. Hermsen CC, de Vlas SJ, van Gemert GJ, Telgt DS, Verhage DF, Sauerwein RW. Testing vaccines in human experimental malaria: statistical analysis of parasitemia measured by a quantitative real-time polymerase chain reaction. Am J Trop Med Hyg 2004; 71(2):196-201.

28. Hoffman SL, Edelman R, Bryan JP et al. Safety, immunogenicity, and efficacy of a malaria sporozoite vaccine administered with monophosphoryl lipid A, cell wall skeleton of mycobacteria, and squalane as adjuvant. Am J Trop Med Hyg 1994; 51(5):603-612.

29. Verhage DF, Telgt DS, Bousema JT et al. Clinical outcome of experimental human malaria induced by Plasmodium falciparum-infected mosquitoes. Neth J Med 2005; 63(2):52-58.

30. Sun P, Schwenk R, White K et al. Protective immunity induced with malaria vaccine, RTS,S, is linked to Plasmodium falciparum circumsporozoite protein-specific CD4+ and CD8+ T cells producing IFN-gamma. J Immunol 2003; 171(12):6961-6967.

31. Reece WH, Pinder M, Gothard PK et al. A CD4(+) T-cell immune response to a conserved epitope in the circumsporozoite protein correlates with protection from natural Plasmodium falciparum infection and disease. Nat Med 2004; 10(4):406-410.

32. Schwenk R, Asher LV, Chalom I et al. Opsonization by antigen-specific antibodies as a mechanism of protective immunity induced by Plasmodium falciparum circumsporozoite protein-based vaccine. Parasite Immunol 2003; 25(1):17-25.

33. Doolan DL, Southwood S, Freilich DA et al. Identification of Plasmodium falciparum antigens by antigenic analysis of genomic and proteomic data. Proc Natl Acad Sci U S A 2003; 100(17):9952-9957.

34. Alonso PL, Sacarlal J, Aponte JJ et al. Efficacy of the RTS,S/AS02A vaccine against Plasmodium falciparum infection and disease in young African children: randomised controlled trial. Lancet 2004; 364(9443):1411-1420.

35. Clyde DF, McCarthy VC, Miller RM, Hornick RB. Specificity of protection of man immunized against sporozoite-induced falciparum malaria. Am J Med Sci 1973; 266(6):398-403.

36. Nussenzweig RS, Vanderberg J, Most H, Orton C. Protective immunity produced by the injection of x-irradiated sporozoites of plasmodium berghei. Nature 1967; 216(111):160-162.

37. Clyde DF. Immunization of man against falciparum and vivax malaria by use of attenuated sporozoites. Am J Trop Med Hyg 1975; 24(3):397-401.

38. Rieckmann KH, Beaudoin RL, Cassells JS, Sell KW. Use of attenuated sporozoites in the immunization of human volunteers against falciparum malaria. Bull World Health Organ 1979; 57 Suppl 1:261-265.

39. Hoffman SL, Isenbarger D, Long GW et al. Sporozoite vaccine induces genetically restricted T cell elimination of malaria from hepatocytes. Science 1989; 244(4908):1078-1081.

40. Wang R, Doolan DL, Le TP et al. Induction of antigen-specific cytotoxic T lymphocytes in humans by a malaria DNA vaccine. Science 1998; 282(5388):476-480.

41. Murphy JR, Baqar S, Davis JR, Herrington DA, Clyde DF. Evidence for a 6.5-day minimum exoerythrocytic cycle for Plasmodium falciparum in humans and confirmation that immunization with a synthetic peptide representative of a region of the circumsporozoite protein retards infection. J Clin Microbiol 1989; 27(7):1434-1437.

42. Rickman LS, Jones TR, Long GW et al. Plasmodium falciparum-infected Anopheles stephensi inconsistently transmit malaria to humans. Am J Trop Med Hyg 1990; 43(5):441-445.

43. Davis JR, Murphy JR, Baqar S, Clyde DF, Herrington DA, Levine MM. Estimate of anti-Plasmodium falciparum sporozoite activity in humans vaccinated with synthetic circumsporozoite protein (NANP)3. Trans R Soc Trop Med Hyg 1989; 83(6):748-750.

44. Beier JC, Davis JR, Vaughan JA, Noden BH, Beier MS. Quantitation of Plasmodium falciparum sporozoites transmitted in vitro by experimentally infected Anopheles gambiae and Anopheles stephensi. Am J Trop Med Hyg 1991; 44(5):564-570.

45. Githeko AK, Brandling-Bennett AD, Beier M, Atieli F, Owaga M, Collins FH. The reservoir of Plasmodium falciparum malaria in a holoendemic area of western Kenya. Trans R Soc Trop Med Hyg 1992; 86(4):355-358.

46. Beier JC, Beier MS, Vaughan JA, Pumpuni CB, Davis JR, Noden BH. Sporozoite transmission by Anopheles freeborni and Anopheles gambiae experimentally infected with Plasmodium falciparum. J Am Mosq Control Assoc 1992; 8(4):404-408.

47. Murphy JR, Clyde DF, Herrington DA et al. Continuation of chloroquine-susceptible Plasmodium falciparum parasitemia in volunteers receiving chloroquine therapy. Antimicrob Agents Chemother 1990; 34(4):676-679.

48. Hermsen CC, Telgt DS, Linders EH et al. Detection of Plasmodium falciparum malaria parasites in vivo by real-time quantitative PCR. Mol Biochem Parasitol 2001; 118(2):247-251.

49. Witney AA, Doolan DL, Anthony RM, Weiss WR, Hoffman SL, Carucci DJ. Determining liver stage parasite burden by real time quantitative PCR as a method for evaluating pre-erythrocytic malaria vaccine efficacy. Mol Biochem Parasitol 2001; 118(2):233-245.

50. Bejon P, Andrews L, Andersen RF et al. Calculation of liver-to-blood inocula, parasite growth rates, and preerythrocytic vaccine efficacy, from serial quantitative polymerase chain reaction studies of volunteers challenged with malaria sporozoites. J Infect Dis 2005; 191(4):619-626.

51. McCutchan TF, de lC, V, Lal AA, Gunderson JH, Elwood HJ, Sogin ML. Primary sequences of two small subunit ribosomal RNA genes from Plasmodium falciparum. Mol Biochem Parasitol 1988; 28(1):63-68.

52. Gunderson JH, Sogin ML, Wollett G et al. Structurally distinct, stage-specific ribosomes occur in Plasmodium. Science 1987; 238(4829):933-937.

53. McCutchan TF. Ribosomal RNA for sporozoite detection. Parasitol Today 1988; 4(4):106.

54. James AA, Rossignol PA. Mosquito salivary glands: Parasitological and molecular aspects. Parasitol Today 1991; 7(10):267-271.

55. Demeure CE, Brahimi K, Hacini F et al. Anopheles mosquito bites activate cutaneous mast cells leading to a local inflammatory response and lymph node hyperplasia. J Immunol 2005; 174(7):3932-3940.

56. Severe falciparum malaria. World Health Organization, Communicable Diseases Cluster. Trans R Soc Trop Med Hyg 2000; 94 Suppl 1:S1-90.

57. Standards for Blood Banks and Transfusion Services. 23 ed. Bethesda, MD: 2004.

58. Gaziano TA, Young CR, Fitzmaurice G, Atwood S, Gaziano JM. Laboratory-based versus non-laboratory-based method for assessment of cardiovascular disease risk: the NHANES I Follow-up Study cohort. Lancet 2008; 371(9616):923-931.

59. Sardelis MR, Edelman R, Klein TA et al. Limited potential for transmission of live dengue virus vaccine candidates by Aedes aegypti and Aedes albopictus. Am J Trop Med Hyg 2000; 62(6):698-701.

60. Davis JR. Laboratory methods for the conduct of experimental malaria challenge of volunteers. Vaccine 1994; 12(4):321-327.

61. Jin Y, Kebaier C, Vanderberg J. Direct microscopic quantification of dynamics of Plasmodium berghei sporozoite transmission from mosquitoes to mice. Infect Immun 2007; 75(11):5532-5539.

62. Rosenberg R, Wirtz RA, Schneider I, Burge R. An estimation of the number of malaria sporozoites ejected by a feeding mosquito. Trans R Soc Trop Med Hyg 1990; 84(2):209-212.

63. Price R, Nosten F, Simpson JA et al. Risk factors for gametocyte carriage in uncomplicated falciparum malaria. Am J Trop Med Hyg 1999; 60(6):1019-1023.

64. Hanscheid T. Diagnosis of malaria: a review of alternatives to conventional microscopy. Clin Lab Haematol 1999; 21(4):235-245.
